# Evaluation of Photophysical Properties and Biological Applications of Diarylmethanes

**DOI:** 10.1002/open.202500601

**Published:** 2026-05-25

**Authors:** H. Ranjini Jenifer, Fateh V. Singh, M. M. Balamurali

**Affiliations:** ^1^ Department of Chemistry School of Advanced Sciences, Vellore Institute of Technology Chennai India; ^2^ Centre for Healthcare Advancement, Innovation and Research Vellore Institute of Technology Chennai India

**Keywords:** anticancer activity, diarylmethane, PARP inhibitor, tamoxifen

## Abstract

Diarylmethane derivatives represent a versatile class of organic molecules with promising therapeutic relevance owing to their structural diversity and favorable drug‐like attributes. Although their synthesis has been widely studied, their biomedical potential remains comparatively underexplored. In this work, a panel of biaryl‐ and teraryl‐cored diarylmethanes was examined for their electronic and photophysical characteristics, alongside an assessment of their biological activities through integrated experimental and computational approaches. The compounds displayed significant binding affinity toward biomacromolecules (BSA as 216.12 M^−1^, while for ct‐DNA, it is 5.47 × 10^5^ M^−1^), including proteins and nucleic acids. To further probe their anticancer potential, molecular docking was performed against the active site of PARP1 (PDB ID: 5HA9), a key enzyme implicated in cancer progression, followed by molecular dynamic simulations to elucidate binding mechanisms. Computational findings indicated a favorable docking pose and robust interaction profile within the PARP1 binding pocket, with minimal perturbation to protein stability and conformation. The anti‐inflammatory properties of the synthesized derivatives were evaluated via protein‐denaturation inhibition assays. Moreover, AI‐driven in silico tools were employed to predict the anticancer activity. Additionally, pharmacokinetic and pharmacodynamic analyses were conducted to assess their drug‐likeness.

## Graphical abstract

The wide applications of diarylmethanes were investigated through various experimental and computational approaches. The solvent environment influences the electronic properties of diarylmethanes and hence their drug‐like properties. Interactions with PARP1 revealed stable binding supporting anticancer potential.

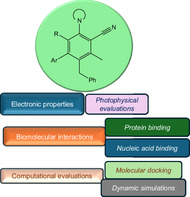

## 1 Introduction

Cancer is a multifaceted and prevalent disease that has a major global impact on morbidity and mortality rates, endangering human health. WHO foresees that by 2035, the number of cancer deaths would increase dramatically to over 14.5 million out of an expected 24 million new cases, suggesting a doubling of the number of cancer‐related deaths in the next two decades [1]. Despite being the most common cancer, affecting women, breast cancer hides a dark secret due to its complex nature, diverse characteristics, treatment hurdles, and influence on public health, leaving a devastating legacy that can ripple through future generations. In the oncological paradigm, managing cellular migration is paramount for achieving tumor control [2, 3, 4]. This is particularly crucial for the aggressive subtypes like triple‐negative breast cancer (TNBC), which exhibit poor visceral metastatic outcomes and limited progression‐free survival. Deciphering the migratory behavior of these recurrent and metastatic TNBC phenotypes, it holds the key toward developing novel therapeutic strategies [5].

Till date, tamoxifen is more commonly prescribed to fight against TNBC [6, 7, 8, 9, 10]. Unlike traditional treatments that target every cell in their path, tamoxifen acts with precision and is an estrogen receptor selective ligand that is being used as common breast cancer therapeutic for many years. It inhibits cell proliferation and blocks the effects of estrogen by attaching itself to the estrogen receptors in the cells. It is less toxic to healthy cells, more effective at lower dosages, and more selective and displays low side effects [11].

**FIGURE 1:**
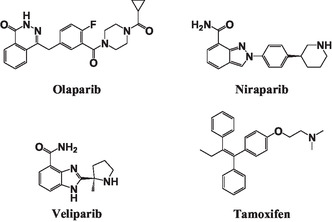
Small‐molecule inhibitors for PARP.

Poly(ADP‐ribose) polymerases (PARPs) belong to a family of proteins that have key roles including their catalyzing ability to transfer ADP‐ribose to target proteins. Moreover, inhibiting this enzyme could potentially block the repair work in cancer cells and ultimately kills them. Among the PARP family, PARP1 and PARP2 are the most studied members and hold the most promising druggable targets for developing new therapeutic strategies [12, 13]. In particular, the PARP1 (poly(ADP‐ribose) polymerase enzyme plays a critical role in fixing single‐strand DNA breaks [14, 15, 16, 17, 18]. Researchers are developing PARP inhibitor (PARPi) to target and kill cancer cells with weak DNA repair mechanisms. These small‐molecule inhibitors, like olaparib, niraparib, and veliparib, have been green lit by the US FDA for treating ovarian cancer, overcoming the challenge of precise targeting of the PARP1/2 enzyme (Figure 1) [19, 20, 21].

Diarylmethanes (DAMs) are small organic molecules with a central methylene group and 1,1‐diaryl units are known for their therapeutic, pharmaceutical, and medicinal applications [22, 23, 24]. They combat infections, bacteria, fungi, inflammation, and cancer and are building blocks for numerous drugs and natural products [25, 26, 27]. A novel diarylmethane skeleton was synthesized to bind the vitamin D receptor (VDR), a key target for breast cancer. This nonsecosteroidal VDR agonist suppresses tumor cell proliferation without side effects [28, 29]. This study paved the way for designing promising cancer chemotherapeutics. Similarly, the pyridylated bis(aryl)methanes have also shown their potential as effective anticancer agents [30, 31, 32]. A study evaluated the in vitro inhibition potential of diarylmethane derivatives on carbohydrate‐metabolizing enzymes. The study found that hindering α‐glycosidase and amylolytic enzymes can delay gastrointestinal absorption and digestion, lowering postprandial glucose levels [32]. Novel inhibitors with diarylmethane backbones effectively inhibited uric acid transporter 1, with IC_50_ values several orders of magnitude lower than known inhibitors [33]. Recently, Qi et al. have reported the significance and scope of bis(indolyl)methane derivatives as potent anticancer agents [34]. Ashish et al. have revealed the anticancer potential of bisindolemethane derivatives via various experimental and chemoinformatic approaches. These derivatives were proven to induce apoptotic cell death of cancer cells [35].

In general, the electronic properties of small molecules significantly influence their biological activities, like photodynamic therapy, wherein the light activated photosensitizers kill cancer cells. On the other hand, luminescent molecules are useful for biomedical imaging due to their high specificity and selectivity, allowing for monitoring drug interactions and disease progression [36]. Therefore, developing efficient luminescent molecules is crucial for various theranostic applications [37].

Our group has been involved in synthesizing libraries of small molecules, and their photophysical and biological properties including anticancer, antibacterial, anti‐ inflammatory, etc*.* were evaluated for their potential applications as diagnostic and therapeutic probes. Herein, a series of 10 different biaryl/teraryl‐cored diarylmethane derivatives were synthesized and evaluated for the electronic properties and bioactivities via various computational and experimental approaches further to envisage their biomedical and theranostic application potentials. Further, the anticancer potentials were assessed through molecular docking and dynamic simulation approaches along with their ability to bind to biomacromolecules (BSA and calf thymus DNA). The present investigations can open new leads by further tuning the structure–activity relationships to enhance their effective biological application potentials.

## 2 Methods

### 2.1 Synthesis

**SCHEME 1:**
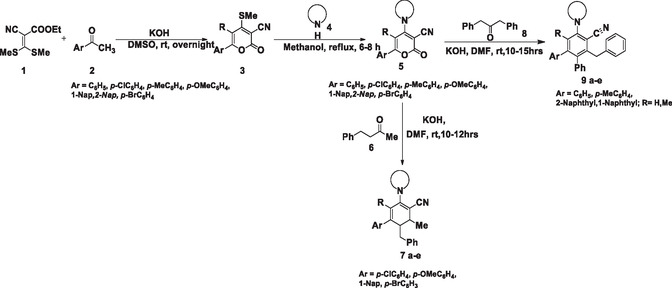
Synthesis of 2‐benzyl‐4′‐chloro‐3‐methyl‐5‐(piperidin‐1‐yl)[1,1′‐biphenyl]‐4 carbonitrile (**7a**) and 3′‐benzyl‐5′‐(piperidin‐1‐yl)‐[1,1′, 2′,1″‐terphenyl]‐4′‐carbonitrile (**9a)** via ring transformation of 6‐phenyl‐2‐oxo‐4‐(piperidin‐1‐yl)‐2*H*‐pyran‐3‐carbonitrile **5** with methyl 2‐phenylethyl ketone (**6**)/1,3‐diphenylpropan‐2‐one (8).

Though the synthetic scheme for the herein proposed molecules is reported in the literature, no further characterizations on their electronic properties and biophysical evaluations are available. Therefore, the previously reported synthetic scheme was adopted with slight modifications [38]. A two‐step synthetic route for the preparation of parent precursor (**5)** is shown in Scheme 1. Initially, a base‐catalyzed reaction of ethyl‐2‐cyano‐3,3‐bis(methylthio)‐2‐propenoate (1 equiv.) (**1)** was combined with functionalized acetophenone (1.2 equiv.) (**2)** in DMSO (10 mL) at room temperature under basic conditions provided by potassium hydroxide (KOH) (1.5 equiv.), yielding the cyclized substrate (**3)**. This cyclized substrate (**3)** was then reacted with various cyclic secondary amines (1.2 equiv.) under refluxing methanol conditions to yield 6‐phenyl‐2‐oxo‐4‐(piperidin‐1‐yl)‐2H‐pyran‐3‐carbonitrile **(5)**.

**FIGURE 2:**
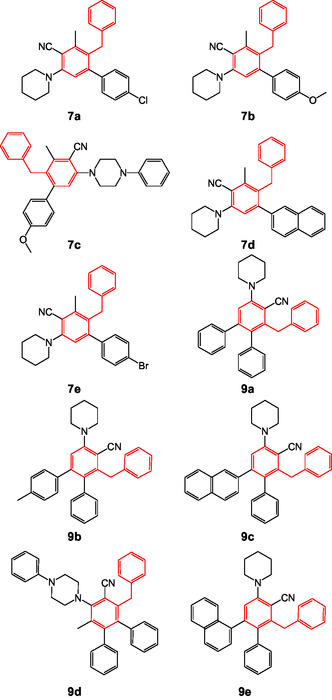
Structures of diarylmethanes.

Herein, we have reported the synthesis of biaryl/teraryl‐cored diarylmethanes (Figure 2) via ring transformation of 6‐phenyl‐2‐oxo‐4‐(piperidin‐1‐yl)‐2H‐pyran‐3‐carbonitrile (**5)** following the literature reported procedure as depicted in Scheme 1, and the relevant data are compiled in Table 1 [38, 39]. A carbanion‐induced ring transformation reaction was followed to synthesize the target compound 2‐benzyl‐4′‐chloro‐3‐methyl‐5‐(piperidin‐1‐yl)[1,1′‐biphenyl]‐4 carbonitrile **7a**. 6‐Phenyl‐2‐oxo‐4‐(piperidin‐1‐yl)‐2H‐pyran‐3‐carbonitrile (1 equiv.) (**5)** is reacted with a nucleophilic source methyl 2‐phenylethyl ketone (1.2 equiv.) (**6)**. The above reaction was carried out in 5 mL of alkaline DMF at room temperature to afford the product in 85% yield. Similarly, the same synthetic procedure was employed for the synthesis of teraryl‐cored diarylmethanes **(9a–e)**, and the same substrate 6‐phenyl‐2‐oxo‐4‐(piperidin‐1‐yl)‐2H‐pyran‐3‐carbonitrile (**5)** was treated with 1,3‐diphenylpropan‐2‐one **(8)** in alkaline DMF at room temperature and obtained 3′‐benzyl‐5′‐(piperidin‐1‐yl)‐[1,1′,2′,1″‐terphenyl]‐4′‐carbonitrile **9a** in 88% yield. The versatile precursor starting material (**5)** (6‐aryl‐4‐amino‐2H‐pyran‐2‐one) thrived in the ring transformation reaction, by accommodating a range of electron‐donating and withdrawing substituents at C‐6 aryl ring displaying enhanced reactivity with various cyclic secondary amines at C‐4. The presence of thiomethyl (‐SMe) moiety potentially increases the susceptibility of C‐4 position for nucleophilic attack that further can lead to the formation of an off‐target product. To minimize this off‐target product, the C‐4 position was substituted with various cyclic amines. The substrate and parent precursor were characterized using techniques such as ^1^H‐NMR, ^13^C‐NMR, and HR‐MS [38] (Supporting Information). The method employed for synthesizing the herein reported biaryl/teraryl‐cored diarylmethanes follows a metal‐free approach that utilizes a carbanion‐induced ring transformation reaction of 2H‐pyran‐2‐ones [38].

**TABLE 1:** Synthesis of biaryl‐cored‐diarylmethane (7a–e) and teraryl‐cored diarylmethane (9a–e) via ring transformation reaction of 6‐phenyl‐2‐oxo‐4‐(piperidin‐1‐yl)‐2*H*‐pyran‐3‐carbonitrile 5 with methyl 2‐phenylethyl ketone (6)/1,3‐diphenylpropan‐2‐one (8).

Entry	Ar	R	Amine	Time, h	% Yield
**7a**	4‐ClC_6_H_4_	H	Piperidin‐1‐yl	14	78
**7b**	4‐MeOC_6_H_4_	H	Piperidin‐1‐yl	10	90
**7c**	4‐MeOC_6_H_4_	H	4‐Phenylpiperazin‐1‐y	10	92
**7d**	2‐Naphthyl	H	Piperidin‐1‐yl	10	88
**7e**	4‐BrC_6_H_4_	H	Piperidin‐1‐yl	10	86
**9a**	Ph	H	Piperidin‐1‐yl	10	88
**9b**	4‐MeC_6_H_4_	H	Piperidin‐1‐yl	10	95
**9c**	2‐Naphthyl	H	Piperidin‐1‐yl	10	90
**9d**	Ph	CH_3_	4‐Phenylpiperazin‐1yl	12	74
**9e**	1‐Naphthyl	H	Piperidin‐1‐yl	10	90

#### 2.1.1 2‐Benzyl‐4′‐Chloro‐3‐Methyl‐5‐(Piperidin‐1‐yl)[1,1′‐Biphenyl]‐4‐Carbonitrile (7a)

White solid; yield: 312 mg (0.77 mmol, 78%); mp 141°C–143°C; *R*_f_ = 0.5 (EtOAc–hexane 1:49). IR (ATR): 2218 cm^–1^ (C≡N). ^1^H NMR (400 MHz, CDCl_3_): *δ* = 1.46–1.57 (m, 2H, CH_2_), 1.66–1.75 (m, 4H, 2 × CH_2_), 2.30 (s, 3H, CH_3_), 3.06 (t, *J* = 5.6 Hz, 4H, 2 × NCH_2_), 3.81 (s, 2H, CH_2_), 6.65 (s, 1H, ArH), 6.80 (d, *J* = 7.2 Hz, 2H, ArH), 7.01 (d, *J* = 8.4 Hz, 2H, ArH), 7.04–7.21 (m, 5H, ArH). ^13^C NMR (100 MHz, CDCl_3_): *δ* = 18.9, 24.1, 26.2, 35.6, 53.5, 107.7, 117.9, 118.2, 126.0, 127.7, 128.4, 128.6, 129.4, 129.9, 133.7, 139.8, 140.0, 143.5, 146.9, 155.7. HRMS (ESI‐TOF): *m*/*z* = 401.1789 [M + 1]^+^, 403.1759 [M + 2]^+^. Anal. Calcd for C_26_H_25_ClN_2_: C, 77.89; H, 6.28; N, 6.99. Found: C, 77.39; H, 6.41; N, 6.43.

#### 2.1.2 2‐Benzyl‐4′‐Methoxy‐3‐Methyl‐5‐(Piperidin‐1‐yl)[1,1′‐Biphenyl]‐4‐Carbonitrile (7b)

White solid; yield: 356 mg (0.90 mmol, 90%); mp 172°C–174°C; *R*_f_ = 0.5 (EtOAc–hexane 1:49). IR (ATR): 2213 cm^–1^ (C≡N). ^1^H NMR (400 MHz, CDCl_3_): *δ* = 1.45–1.58 (m, 2H, CH_2_), 1.66–1.75 (m, 4H, 2 × CH_2_), 2.28 (s, 3H, CH_3_), 3.05 (t, *J* = 5.2 Hz, 4H, 2 × NCH_2_), 3.71 (s, 3H, OCH_3_), 3.86 (s, 2H, CH_2_), 6.70 (s, 1H, ArH), 6.75 (d, *J* = 8.0 Hz, 2H, ArH), 6.83 (d, *J* = 7.2 Hz, 2H, ArH), 7.02 (d, *J* = 8.0 Hz, 2H, ArH), 7.04–7.10 (m, 1H, ArH), 7.14 (t, *J* = 7.2 Hz, 2H, ArH). ^13^C NMR (100 MHz, CDCl_3_): *δ* = 18.9, 24.2, 26.3, 35.7, 53.6, 55.3, 107.2, 113.6, 118.2, 118.5, 125.9, 127.8, 128.5, 129.7, 133.8, 140.4, 143.3, 147.9, 155.6, 159.1. HRMS (ESI‐TOF): *m*/*z* = 397.2282 [M + 1]^+^. Anal. Calcd for C_27_H_28_N_2_O: C, 81.78; H, 7.12; N, 7.06. Found: C, 81.11; H, 7.14; N, 6.92.

#### 2.1.3 2‐Benzyl‐4′‐Methoxy‐3‐Methyl‐5‐(4‐Phenylpiperazin‐1‐yl)[1,1′‐Biphenyl]‐4‐Carbonitrile (7c)

White solid; yield: 435 mg (0.92 mmol, 92%); mp 174°C–176°C; *R*_f_ = 0.5 (EtOAc–hexane 1:49). IR (ATR): 2214 cm^–1^ (C≡N). ^1^H NMR (400 MHz, CDCl_3_): *δ* = 2.31 (s, 3H, CH_3_), 3.25–3.36 (m, 8H, 4 × NCH_2_), 3.72 (s, 3H, OCH_3_), 3.89 (s, 2H, CH_2_), 6.75–6.77 (m, 2H, ArH), 6.78 (s, 1H, ArH), 6.81–6.87 (m, 2H, ArH), 6.88–6.95 (m, 2H, ArH), 7.04 (d, *J* = 8.8 Hz, 2H, ArH), 7.06–7.12 (m, 1H, ArH), 7.13–7.24 (m, 5H, ArH). ^13^C NMR (100 MHz, CDCl_3_): *δ* = 19.0, 35.7, 49.6, 51.9, 55.3, 107.3, 113.7, 116.4, 118.5, 120.1, 126.0, 127.8, 128.5, 128.7, 129.2, 129.7, 130.8, 133.5, 140.2, 143.7, 148.1, 151.2, 154.1, 159.2. HRMS (ESI‐TOF): *m*/*z* = 474.2545 [M + 1]+. Anal. Calcd for C_3__2_H_31_N_3_O: C, 81.15; H, 6.60; N, 8.87. Found: C, 81.02; H, 7.12; N, 8.71.

#### 2.1.4 3‐Benzyl‐2‐Methyl‐4‐(Naphthalen‐2‐yl)‐6‐(Piperidin‐1‐yl)Benzonitrile (7d)

White solid; yield: 366 mg (0.88 mmol, 88%); mp 162°C–164°C; *R*_f_ = 0.5 (EtOAc–hexane 1:49). IR (ATR): 2213 cm^–1^ (C≡N). ^1^H NMR (400 MHz, CDCl_3_): *δ* = 1.44–1.55 (m, 2H, CH_2_), 1.66–1.76 (m, 4H, 2 × CH_2_), 2.33 (s, 3H, CH_3_), 3.07 (t, *J* = 5.2 Hz, 4H, 2 × NCH_2_), 3.87 (s, 2H, CH_2_), 6.79 (s, 1H, ArH), 6.82 (d, *J* = 6.8 Hz, 2H, ArH), 7.07 (d, *J* = 6.8 Hz, 1H, ArH), 7.13 (t, *J* = 7.6 Hz, 2H, ArH), 7.21 (dd, *J*_1_ = 8.4 Hz, *J*_2_ = 1.6 Hz, 1H, ArH), 7.35–7.41 (m, 2H, ArH), 7.54 (s, 1H, ArH), 7.58–7.65 (m, 1H, ArH), 7.68 (d, *J* = 8.8 Hz, 1H, ArH), 7.70–7.78 (m, 1H, ArH). ^13^C NMR (100 MHz, CDCl_3_): *δ* = 19.0, 24.2, 26.3, 35.8, 53.6, 107.6, 118.1, 118.6, 125.9, 126.3, 126.4, 126.8, 127.5, 127.6, 127.7, 127.8, 128.1, 128.5, 129.8, 132.5, 133.0, 138.9, 140.3, 143.5, 148.1, 155.7. HRMS (ESI‐TOF): *m*/*z* = 417 [M + 1]^+^. Anal. Calcd for C_30_H_28_N_2_: C, 86.50; H, 6.78; N, 6.72. Found: C, 86.17; H, 6.77; N, 6.60.

#### 2.1.5 2‐Benzyl‐4′‐Bromo‐3‐Methyl‐5‐(Piperidin‐1‐yl)[1,1′‐Biphenyl]‐4‐Carbonitrile (7e)

White solid; yield: 364 mg (0.82 mmol, 82%); mp 116°C–118°C; *R*_f_ = 0.5 (EtOAc–hexane 1:49). IR (ATR): 2217 cm^–1^ (C≡N). ^1^H NMR (400 MHz, CDCl_3_): *δ* = 1.47–1.56 (m, 2H, CH_2_), 1.66–1.73 (m, 4H, 2 × CH_2_), 2.30 (s, 3H, CH_3_), 3.06 (t, *J* = 4.8 Hz, 4H, 2 × NCH_2_), 3.81 (s, 2H, CH_2_), 6.65 (s, 1H, ArH), 6.80 (d, *J* = 7.6 Hz, 2H, ArH), 6.95 (d, *J* = 8.0 Hz, 2H, ArH), 7.03–7.19 (m, 3H, ArH), 7.34 (d, *J* = 8.0 Hz, 2H, ArH). ^13^C NMR (100 MHz, CDCl_3_): *δ* = 18.9, 24.1, 26.2, 35.6, 53.5, 107.7, 117.9, 118.1, 121.9, 126.1, 127.7, 128.6, 129.4, 130.2, 131.3, 140.0, 140.2, 143.6, 146.8, 155.7. HRMS (ESI‐TOF): *m*/*z* = 445.1283 [M + 1]^+^, 447.1269 [M + 2]^+^. Anal. Calcd for C_26_H_25_BrN_2_: C, 70.11; H, 5.66; N, 6.29. Found: C, 70.03; H, 5.66; N, 6.11.

#### 2.1.6 3′‐Benzyl‐5′‐(Piperidin‐1‐yl)[1,1′:2′,1″‐Terphenyl]‐4′‐Carbonitrile (9a)

White solid; yield: 376 mg (0.88 mmol, 88%); mp 145°C–148°C; *R*_f_ = 0.5 (EtOAc–hexane 1:49). IR (ATR): 2214 cm^–1^ (C≡N). ^1^H NMR (400 MHz, CDCl_3_): *δ* = 1.49–1.58 (m, 2H, CH_2_), 1.69–1.78 (m, 4H, 2 × CH_2_), 3.13 (t, *J* = 5.2 Hz, 4H, 2 × NCH_2_), 4.06 (s, 2H, CH_2_), 6.71–6.81 (m, 3H, ArH), 6.86 (s, 1H, ArH), 6.90–6.95 (m, 2H, ArH), 6.96–7.10 (m, 9H, ArH), 7.17–7.26 (m, 1H, ArH). ^13^C NMR (100 MHz, CDCl_3_): *δ* = 24.1, 26.2, 38.4, 53.6, 107.2, 118.9, 125.9, 126.8, 126.9, 127.6, 127.7, 128.1, 128.5, 129.4, 130.9, 135.0, 138.1, 138.2, 139.5, 141.1, 144.3, 147.1, 156.8. HRMS (ESI‐TOF): *m*/*z* = 429.2332 [M + 1]^+^. Anal. Calcd for C_31_H_28_N_2_: C, 86.88; H, 6.59; N, 6.54. Found: C, 86.82; H, 6.69; N, 5.97.

#### 2.1.7 3′‐Benzyl‐4‐Methyl‐5′‐(Piperidin‐1‐yl)[1,1′:2′,1″‐Terphenyl]‐4′‐Carbonitrile (9b)

White solid; yield: 419 mg (0.95 mmol, 95%); mp 156°C–158°C; *R*_f_ = 0.5 (EtOAc–hexane 1:49). IR (ATR): 2210 cm^–1^ (C≡N). ^1^H NMR (400 MHz, CDCl_3_): *δ* = 1.47–1.57 (m, 2H, CH_2_), 1.68–1.77 (m, 4H, 2 × CH_2_), 2.15 (s, 3H, CH_3_), 3.12 (t, *J* = 5.2 Hz, 4H, 2 × NCH_2_), 4.04 (s, 2H, CH_2_), 6.76 (t, *J* = 8.4 Hz, 4H, ArH), 6.80–6.87 (m, 5H, ArH), 6.97–7.10 (m, 6H, ArH). ^13^C NMR (100 MHz, CDCl_3_): *δ* = 21.1, 24.1, 26.2, 38.4, 53.6, 106.9, 118.2, 119.0, 125.9, 126.7, 127.7, 128.1, 128.4, 128.5, 129.3, 130.9, 135.0, 136.6, 138.1, 138.4, 139.5, 144.3, 147.1, 156.8. HRMS (ESI‐TOF): *m*/*z* = 443.2488 [M + 1]^+^. Anal. Calcd for C_32_H_30_N_2_: C, 86.84; H, 6.83; N, 6.33. Found: C, 86.77; H, 6.82; N, 6.24.

#### 2.1.8 2‐Benzyl‐6‐(Naphthalen‐2‐yl)‐4‐(Piperidin‐1‐yl)[1,1′‐Biphenyl]‐3‐Carbonitrile (9c)

White solid; yield: 430 mg (0.90 mmol, 90%); mp 182°C–184°C; *R*_f_ = 0.5 (EtOAc–hexane 1:49). IR (ATR): 2214 cm^–1^ (C≡N). ^1^H NMR (400 MHz, CDCl_3_): *δ* = 1.63–1.71 (m, 2H, CH_2_), 1.82–1.90 (m, 4H, 2 × CH_2_), 3.28 (t, *J* = 5.2 Hz, 4H, 2 × NCH_2_), 4.22 (s, 2H, CH_2_), 6.90–6.96 (m, 4H, ArH), 7.08–7.13 (m, 5H, ArH), 7.14–7.23 (m, 3H, ArH), 7.42–7.50 (m, 2H, ArH), 7.56 (d, *J* = 8.4 Hz, 1H, ArH), 7.64 (s, 1H, ArH), 7.71–7.78 (m, 2H, ArH). ^13^C NMR (100 MHz, CDCl_3_): *δ* = 24.2, 26.2, 38.4, 53.6, 107.3, 118.2, 119.3, 125.9, 126.1, 126.2, 126.8, 126.9, 127.4, 127.6, 127.8, 127.9, 128.1, 128.4, 128.5, 130.9, 132.1, 132.9, 135.1, 138.2, 138.8, 139.5, 144.5, 147.0, 156.9. HRMS (ESI‐TOF): *m*/*z* = 479.2490 [M + 1]^+^. Anal. Calcd for C_35_H_30_N_2_: C, 87.83; H, 6.32; N, 5.85. Found: C, 83.69; H, 6.36; N, 5.58.

#### 2.1.9 3′‐Benzyl‐6′‐Methyl‐5′‐(4‐Phenylpiperazin‐1‐yl)[1,1′:2′,1″‐Terphenyl]‐4′‐Carbonitrile (9d)

White solid; yield: 384 mg (0.74 mmol, 74%); mp 178°C–180°C; *R*_f_ = 0.5 (EtOAc–hexane 1:49). IR (ATR): 2217 cm^–1^ (C≡N). ^1^H NMR (400 MHz, CDCl_3_): *δ* = 1.99 (s, 3H, CH_3_), 3.22–3.60 (m, 8H, 4 × NCH_2_), 3.98 (s, 2H, CH_2_), 6.64 (dd, *J*_1_ = 7.2 Hz, *J*_2_ = 1.6 Hz, 2H, ArH), 6.78 (dt, *J*_1_ = 7.2 Hz, *J*_2_ = 1.6 Hz, 5H, ArH), 6.89–6.96 (m, 5H, ArH), 6.95–7.09 (m, 6H, ArH), 7.20 (t, *J* = 8.4 Hz, 2H, ArH). ^13^C NMR (100 MHz, CDCl_3_): *δ* = 17.2, 38.2, 50.5, 50.7, 111.4, 116.6, 118.5, 120.0, 126.0, 126.6, 127.4, 127.7, 128.1, 128.5, 129.1, 129.3, 130.3, 133.9, 138.6, 139.5, 139.6, 139.9, 141.4, 148.2, 151.8, 152.8. HRMS (ESI‐TOF): *m*/*z* = 520.2852 [M + 1]^+^. Anal. Calcd for C_37_H_33_N_3_: C, 85.51; H, 6.40; N, 8.09. Found: C, 85.38; H, 6.70; N, 8.07.

#### 2.1.10 2‐Benzyl‐6‐(Naphthalen‐1‐yl)‐4‐(Piperidin‐1‐yl)‐[1,1′Biphenyl]‐3‐Carbonitrile (9e)

White solid; yield: 430 mg (0.90 mmol, 90%); mp 178°C–180°C; *R*_f_ = 0.5 (EtOAc–hexane 1:49). IR (ATR): 2217 cm^–1^ (C≡N). ^1^H NMR (400 MHz, CDCl_3_): *δ* = 7.62–7.58 (m, 2H, Ar‐H), 7.46–7.42 (m, 1H, Ar‐H), 7.33–7.31 (m, 1H, Ar‐H), 7.18–7.14 (m, 3H, Ar‐H), 7.08–7.02 (m, 3H, Ar‐H), 6.99–6.96 (m, 5H, Ar‐H), 6.80 (s, 1H, Ar‐H), 6.79 (d, *J* = 7.2 Hz, 2H, Ar‐H), 4.09 (s, 2H, Ar‐CH_2_‐Ar), 3.16–3.14 (m, 4H, piperidine‐H), 1.74–1.72 (m, 4H, piperidine‐H), 1.56–1.52 (m, 2H, piperidine‐H). ^13^C NMR (100 MHz, CDCl_3_): *δ* = 155.9, 146.9, 143.5, 139.8, 137.4, 133.7, 132.1, 131.0, 129.9, 128.5, 128.4, 127.5, 127.4, 127.1, 126.8, 126.6, 125.9, 125.1, 124.9, 118.2, 117.9, 107.7, 52.6, 37.4 (Ar‐CH_2_‐Ar), 25.2, 23.1. HRMS (ESI‐TOF): *m/z* [M+H]^+^ Calcd for C_35_H_31_N_2_ 479.2487. Found: 479.2490.

**SCHEME 2:**
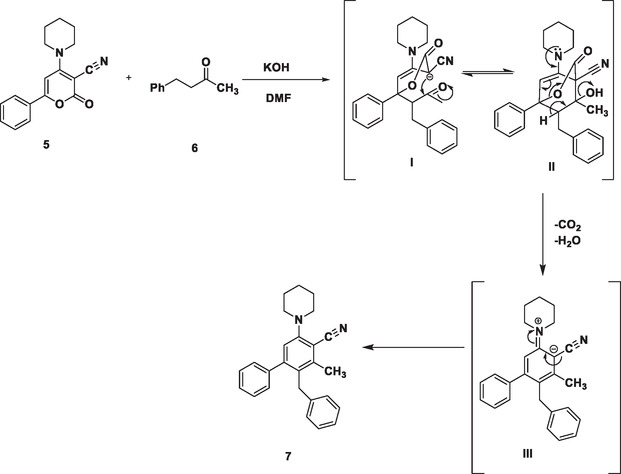
Plausible mechanism toward the synthesis of biaryl‐cored diarylmethane via ring transformation reaction of 2‐*H*‐pyranone (**5)** with methyl 2‐phenylethyl ketone (**6)**.

The mechanism reported for the synthesis of functionalized diarylmethane is depicted in Scheme 2. The unique structure of lactones offers three potential sites (carbon‐2, ‐4, and ‐6) for nucleophilic attack. The carbon‐6 position is more prone to nucleophilic attack as the presence of extended conjugation plays a key role in dictating reactivity; in this case, it creates an electron‐deficient environment at the C‐6 position making it a prime target for nucleophiles seeking electron density. Moreover, the electron density away from C‐6 can be pulled by the presence of electron‐withdrawing group at C‐3 position of pyranone ring to make it more electrophilic.

As shown in Scheme 2, a nucleophilic enolate intermediate is formed in situ via the deprotonation of active methylene group. The later then undergoes a Michael addition reaction at C‐6 position of 6‐phenyl‐2‐oxo‐4‐(piperidin‐1‐yl)‐2H‐pyran‐3‐carbonitrile molecule (**5**) with the formation of a new carbon–carbon bond to yield the intermediate [I] which is further followed by a rapid intramolecular cyclization reaction. The carbanion at C‐3 position of the pyranone ring of intermediate [I] then attacks the carbonyl group within the same molecule to form another carbon–carbon bond further to yield bicyclic intermediate [II] which then undergoes a cascade of reactions including decarboxylation and dehydration to yield intermediate [III]. The final product which is a highly functionalized diarylmethane (**7**) is formed by the aromatization of [III].

## 3 Results

### 3.1 Photophysical Investigation—Solvatochromism

The photophysical properties of the herein reported molecules were assessed by following the changes in electronic behavior in their respective electronic ground and excited states under different environmental (polar‐protic and aprotic) conditions. The ground‐state electronic properties of di(aryl)methane derivatives (DAM) (**7a–e**, **9a–e**) were investigated through UV–visible absorption spectra and the excited‐state properties through fluorescence emission and excitation spectra in different solvents of varying polarity and protic nature.

**FIGURE 3:**
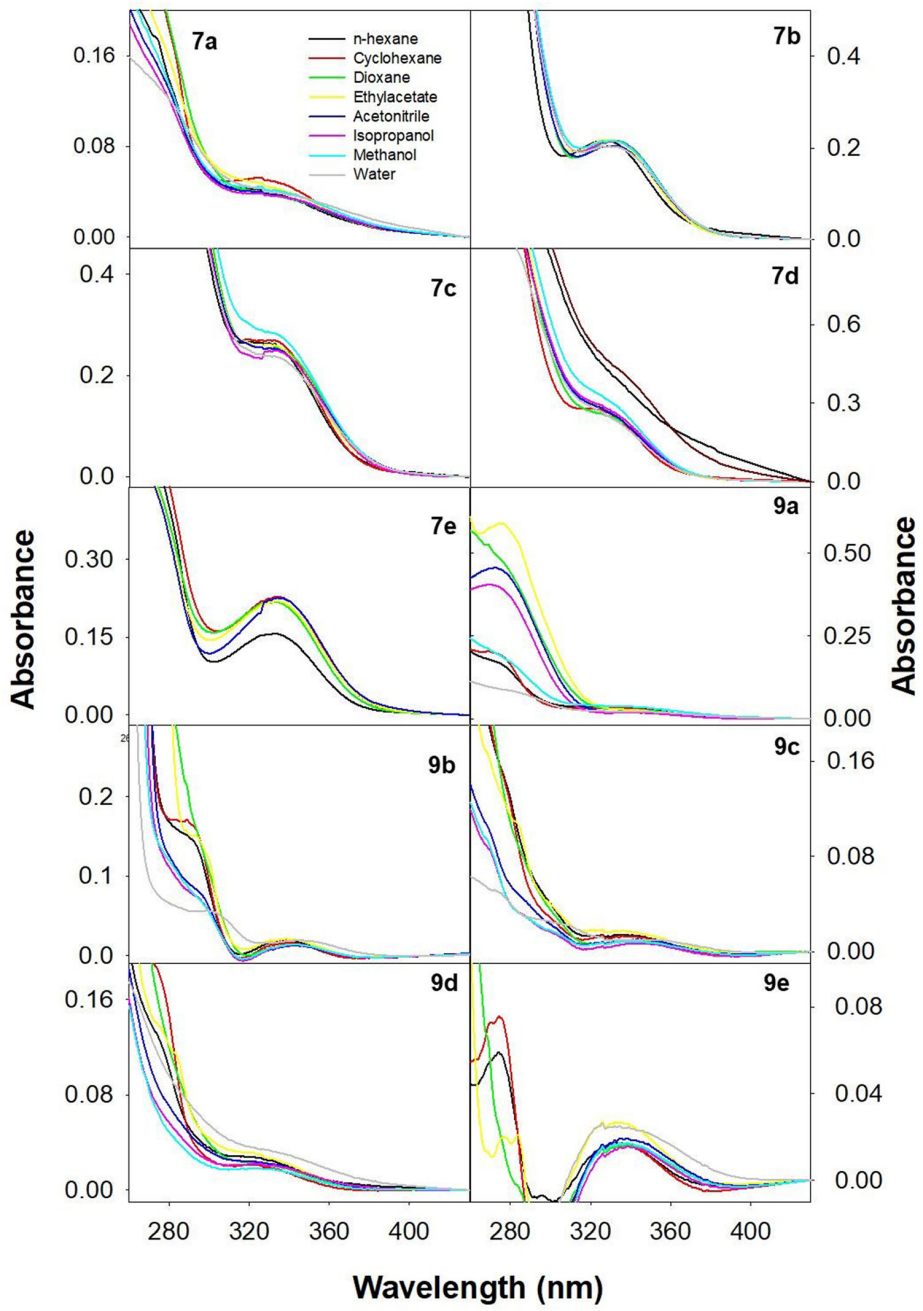
The absorption spectra depicting the solvatochromic effect of various **7a–e** and **9a–e**.

The absorption spectra of all the derivatives were recorded in the range of 250–500 nm, in different solvents as shown in Figure 3. The absorption spectra revealed two peaks ∼265 and ∼330 nm. The shoulder band ∼265 nm attributes to aromatic transitions within the molecular backbone, and the pronounced peak near ∼330 nm corresponds to transitions arising from the various functional groups and their interactions with the surrounding solvent environment. Notably, the absorption maxima at ∼330 nm remain largely unchanged across nonpolar solvents including n‐hexane and cyclohexane, indicating no significant alterations in the fundamental electronic transition energies. However, in polar protic solvents like methanol and water, a noticeable shift in the absorption maxima along with elevated molar extinction values (*ε*) was observed. This phenomenon is particularly prominent in **7b, 7d, 9b**, and **9c**, where extended conjugation and enhanced molecular planarity are expected to induce stronger dipolar and hydrogen‐bonding interactions with heteroatoms. In contrast, **7a** displayed minimal solvent‐dependent variation, suggesting weaker interaction with the surrounding solvent molecules. All these interpretations were made based on the long wavelength absorption band at ∼330 nm, and the pertinent data are compiled in Table 2.

**TABLE 2:** Absorption spectral characteristics of various DAM in different solvents.

	7a	7b	7c	7d	7e
n‐Hexane	264 (sh), 326 (3.37)	264 (4.36), 269 (sh), 329 (3.67)	279 (sh), 331 (4.46)	282 (sh), 321 (4.79)	272 (sh), 332 (4.54)
Cyclohexane	264 (sh), 326 (3.35)	267 (4.39), 330 (3.63)	279 (sh), 331 (4.44)	282 (sh), 321 (4.47)	272 (sh), 333 (4.21)
Dioxane	264 (sh), 326 (3.43)	266 (4.41), 331 (3.64)	279 (sh), 331 (4.42)	282 (sh), 321 (4.44)	272 (sh), 334 (4.37)
Ethylacetate	264 (sh), 326 (3.45)	265 (4.38), 330 (3.65)	279 (sh), 331 (4.42)	282 (sh), 321 (4.48)	272 (sh), 334 (4.35)
Acetonitrile	264 (sh), 326 (3.46)	267 (4.33), 332 (3.63)	279 (sh), 331 (4.41)	282 (sh), 321 (4.48)	272 (sh), 334 (4.35)
Isopropanol	264 (sh), 326 (3.34)	269 (4.34), 332 (3.64)	279 (sh), 331 (4.42)	282 (sh), 321 (4.48)	272 (sh), 334 (4.38)
Ethanol	264 (sh), 326 (3.39)	268 (4.34), 331 (3.64)	279 (sh), 331 (4.46)	282 (sh), 321 (4.55)	272 (sh), 334 (4.38)
Methanol	264 (sh), 326 (3.39)	268 (4.31), 330 (3.62)	279 (sh), 331 (4.49)	283 (sh), 321 (4.46)	272 (sh), 334 (4.39)
Water (pH 7.0)	264 (sh), 326 (3.40)	279 (4.34), 332 (3.91)	269 (5.28), 333 (4.67)	289 (sh), 328 (4.76)	272 (sh), 334 (4.07)
	**9a**	**9b**	**9c**	**9d**	**9e**
n‐Hexane	270 (sh), 326 (4.57)	265 (sh), 334 (4.27)	277 (sh), 336 (4.16)	273 (sh), 319 (4.45)	278 (sh), 331 (4.22)
Cyclohexane	269 (sh), 328 (4.50)	265 (sh), 337 (4.21)	277 (sh), 337 (4.11)	275 (sh), 320 (4.32)	277 (sh), 338 (4.22)
Dioxane	270 (sh), 333 (4.45)	267 (sh), 341 (4.16)	276 (sh), 341 (3.95)	274 (sh), 321 (4.37)	275 (sh), 338 (4.21)
Ethylacetate	275 (sh), 335 (4.46)	267 (sh), 336 (4.34)	277 (sh), 335 (4.24)	273 (sh), 321 (4.50)	275 (sh), 334 (4.42)
Acetonitrile	271 (sh), 337 (4.32)	267 (sh), 342 (4.10)	275 (sh), 342 (4.00)	274 (sh), 321 (4.38)	276 (sh), 336 (4.28)
Isopropanol	269 (sh), 338 (4.27)	267 (sh), 342 (4.16)	276 (sh), 342 (3.84)	275 (sh), 323 (4.34)	277 (sh), 336 (4.17)
Ethanol	270 (sh), 325 (4.61)	267 (sh), 343 (4.16)	275 (sh), 343 (3.94)	276 (sh), 324 (4.27)	275 (sh), 336 (4.22)
Water (pH 7.0)	270 (sh), 330 (4.65)	265 (sh), 346 (4.39)	277 (sh), 342 (4.35)	275 (sh), 325 (4.54)	277 (sh), 336 (4.38)

**FIGURE 4:**
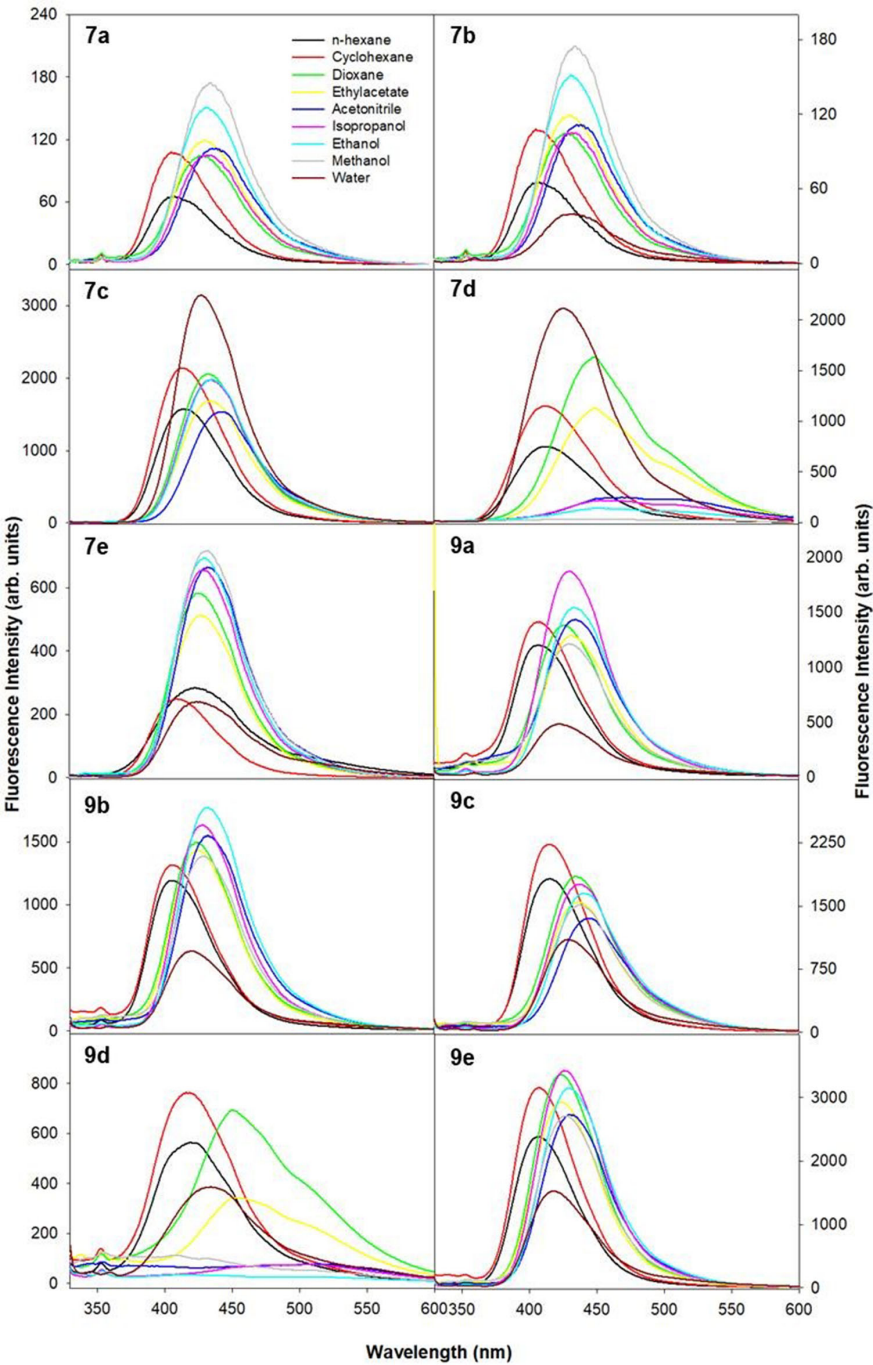
The fluorescence emission spectra depicting the solvatochromic effect of various **7a–e** and **9a–e**. *λ*_ex_ = 320 nm and *λ*_em_ = 330–600 nm.

Similarly, the excited‐state electronic properties of **7a–e** and **9a–e** under different solvent environments were evaluated by following the variations observed in the fluorescence emission spectra (Figure 4) at different excitation wavelengths from the long wavelength absorption bands of the respective molecules. Identical emission spectra across different excitation wavelengths indicate the presence of single species in the ground states, and the same is responsible for the observed fluorescence emission bands. The observed values of full width at half maxima (FWHM) ∼3300 cm^−1^ also ascertain the same. Further, identical excitation spectral pattern at varying emission wavelengths also suggests that the emission has originated from the lowest excited state.

It could be noticed from Figure 4 and Table 3 that a redshift of ∼26, ∼25, ∼21, ∼39, ∼8, ∼25, ∼24, ∼26, ∼81, and ∼21 nm was observed upon changing the polarity and hydrogen‐bonding capacity of the solvents, respectively, for **7a–e** and **9a–e**. This indicates that significant charge redistribution is induced upon excitation which further promotes dipolar interaction with the solvents and induce stabilization (indicative of pronounced intramolecular charge transfer [ICT] involving electron density transfer). Moreover, an increase in quantum yield values with increase in solvent polarity indicates the decrease in the extent of nonradiative transitions. In polar protic solvents, this could be particularly driven by the formation of intermolecular hydrogen bonds with the surrounding solvent molecules. Compounds **7d** and **9d** exhibited prominent redshifts (∼39 and ∼81 nm) compared to other derivatives, along with the observation of structured fluorescence spectra. This suggests an increase in molecular planarity as solvent polarity increases, and this phenomenon was not observed for other derivatives. Their fluorescence quantum yields also showed a highly nonlinear dependence on solvent polarity, reflecting complex excited‐state interactions with the solvent environment. Compound **7e** showed a much smaller redshift of about ∼8 nm, indicating a more rigid molecular structure that limits solvent reorientation in the excited state.

**TABLE 3:** Fluorescence emission spectral characteristics and quantum yield of various DAM in different solvents.

	7a	7b	7c	7d	7e
n‐Hexane	407 (0.05)	398 (0.10)	414 (0.178)	413 (0.013)	423 (0.04)
Cyclohexane	408 (0.06)	399 (0.15)	414 (0.239)	413 (0.0724)	409 (0.05)
Dioxane	430 (0.09)	416 (0.22)	432 (0.246)	449 (0.24), 496 (sh)	425 (0.09)
Ethylacetate	431 (0.08)	414 (0.20)	434 (0.208)	450 (0.16), 496 (so)	428 (0.10)
Acetonitrile	437 (0.09)	423 (0.25)	443 (0.200)	453 (shy), 471 (0.05), 508	433 (0.12)
Isopropanol	431 (0.09)	421 (0.27)	435 (0.240)	451 (0.04), 467 (so), 504	428 (0.12)
Ethanol	432 (‐)	422 (0.27)	435 (0.215)	451 (0.03), 467 (shy), 503	430 (0.19)
Methanol	433 (0.13)	423 (0.29)	435 (0.233)	437 (so), 452 (0.018), 468 (sh), 506	431 (0.22)
Water (pH 7.0)	433 (0.03)	412 (0.10)	428 (0.146)	409 (sh), 449 (0.100), 507	425 (0.05), 452 (sh)
	**9a**	**9b**	**9c**	**9d**	**9e**
n‐Hexane	408 (0.154)	406 (0.139)	414 (0.149)	406 (sh), 418 (0.119)	406 (0.159)
Cyclohexane	408 (0.171)	407 (0.144)	415 (0.155)	418 (0.125)	406 (0.167)
Dioxane	427 (0.176)	423 (0.157)	434 (0.151)	450 (0.129), 496 (sh)	423 (0.169)
Ethylacetate	432 (0.169)	425 (0.152)	436 (0.134)	454 (0.088), 493 (sh)	424 (0.157)
Acetonitrile	434 (0.143)	432 (0.139)	445 (0.124)	386 (sh), 451 (sh), 477 (sh), 512 (0.009)	430 (0.134)
Isopropanol	430 (0.147)	429 (0.154)	438 (0.149)	453 (sh), 481 (sh), 509 (0.008)	427 (0.163)
Ethanol	433 (0.151)	430 (0.161)	440 (0.153)	411 (0.007), 499 (sh)	429 (0.161)
Water (pH 7.0)	430 (0.075)	428 (0.149)	437 (0.129)	407 (0.010), 432, 499 (sh)	427 (0.151)

In polar protic solvents such as IPA, methanol, and ethanol, the excited state is strongly perturbed, with the enhancement of nonradiative decay processes. This could likely be due to specific hydrogen‐bonding interactions that may restrict the excited state's ability to fully relax or twist, further to result in the formation of different conformers or excited states. The broadened spectra along with appearance of multiple shoulder peaks suggest the formation of different conformations, possibly due to more pronounced solvent‐excited‐state interactions, leading to distributed emissive states. This corresponds to a significant drop in quantum yield, indicating favored nonradiative decay mechanisms in highly polar environments.

In aqueous solutions, most compounds showed reduced fluorescence intensity and significant deviations in fluorescence band maxima compared to other solvents, which could likely be due to the formation of ionic species around neutral pH (∼7).

Further to get more insights into the electronic properties of the reported **7a–e** and **9a–e** derivatives, computational density functional theory evaluations were performed with B3LYP functional and 6‐311++G** basis set. All the pertinent data are given in Table S1. The values of dihedral angle indicate the planarity between the aromatic rings of the second aryl substitution of DAMs which is ∼65 deg. For efficient charge distribution, planarity should increase upon excitation as discussed above for the case of **7d**. The most intense electronic transitions with the percentage contributions from various molecular orbitals are also given in Table S1. The electronic distribution pattern in the highest occupied and lowest unoccupied molecular orbitals along with the energy gap is shown in Figure S10. It was revealed that the charge density is mostly localized on the piperidinyl benzonitrile and phenyl piperazinyl benzonitrile moieties in their HOMO, while migration of charges to the aromatic rings happens in their LUMO (Figure S10). For this kind of charge distribution to happen, it is necessary that the planarity of aromatic rings increases upon excitation.

### 3.2 Biological Significance of the Proposed Biaryl/Teraryl‐Cored Diarylmethanes

#### 3.2.1 Biomacromolecular Interactions

##### 3.2.1.1 Protein Binding

The ability of **7a–e** and **9a–e** to interact with BSA protein was assessed by monitoring the changes observed in intrinsic fluorescence emission upon titrating with different concentrations of the DAMs (0–24 µM) against 5 µM BSA in 10 mM Tris‐HCl buffer (pH = 7.2). The quenching interactions were investigated by monitoring the emission intensity at 345 nm at a fixed excitation wavelength of 280 nm. Parameters including the slit widths and emission and excitation scan rates were maintained constant throughout the study.

**FIGURE 5:**
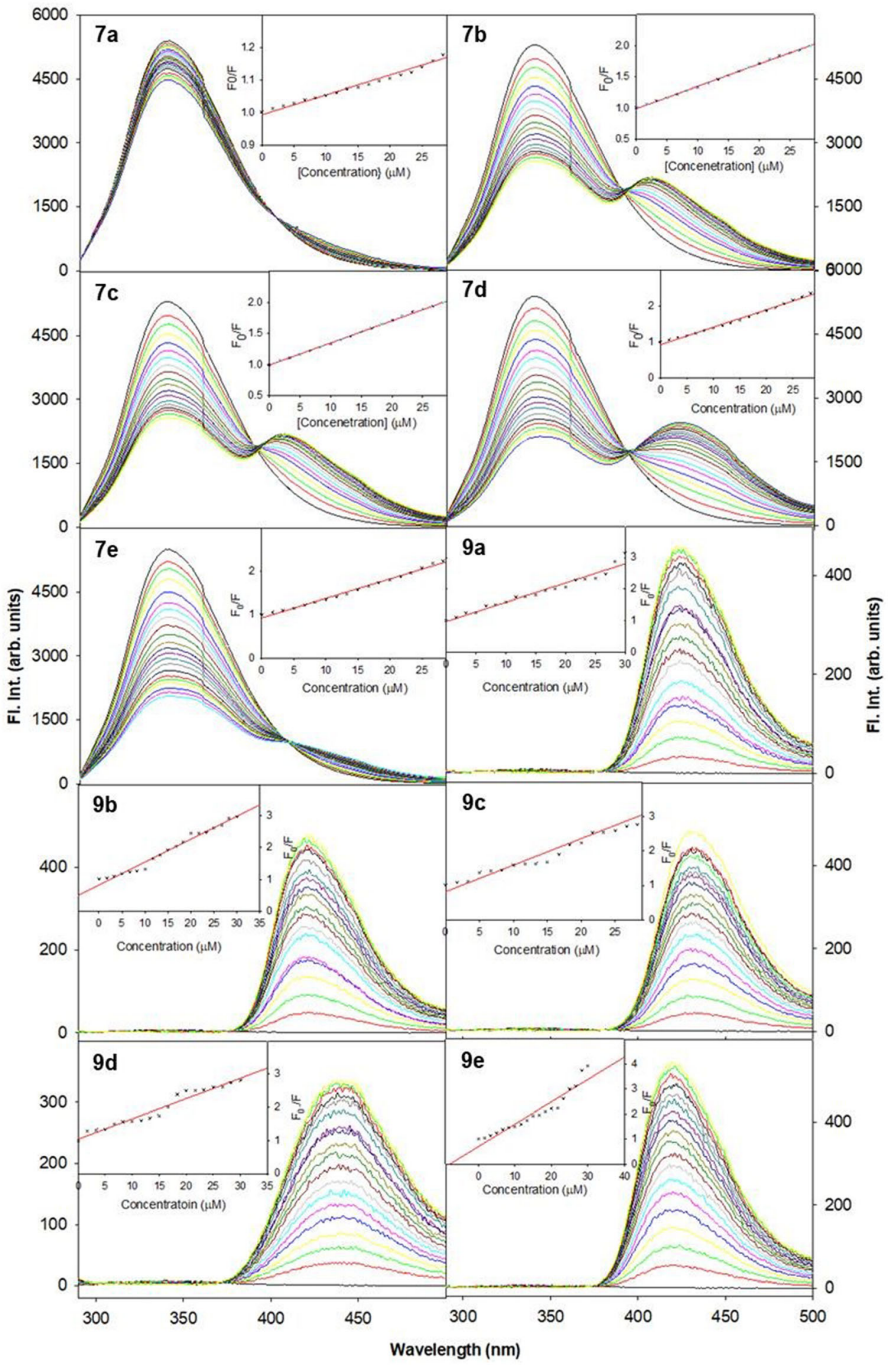
Emission spectra of BSA (0–24 µM) in the presence of increasing concentrations of **7a–e** and **9a–e**. Inset: Stern–Volmer plot of *F*_0_/*F* versus [*Q*] determining the fluorescence quenching constant (*K*_sv_) of various **7a–e** and **9a–e** with BSA interaction.

It could be noticed that upon increasing the concentration of **7a–e** and **9a–e** (0–24 µM), a significant quenching of fluorescence intensity was observed which is further accompanied by the appearance of a new peak ∼425 nm. The appearance of an isoemissive point indicates an equilibrium between the bound and unbound proteins, which further confirms the interaction of **7a–e** and **9a–e** with the BSA protein (Figure 5).

The Stern–Volmer equation was employed to obtain the fluorescence quenching constant.

$$F_{0}/F=1+K_{\mathrm{sv}}[Q]$$

where *F*_0_ and *F* are the observed fluorescence intensities in the absence and in the presence of different concentrations of **7a–e** and **9a–e** (quenchers), respectively. *K*_sv_ is the Stern–Volmer quenching constant which was determined by plotting *I*_0_/*I* vs. [*Q*] which is the concentration of DAMs (Table 4).

**TABLE 4:** Evaluation of the number of binding sites, equilibrium binding constant, and Stern–Volmer quenching constant for the interaction of 7a–e and 9a–e with BSA protein.

Entry	***K***_**SV**_ **, µM** ^**−1**^	***K***_**b**_ **× 10** ^**4**^ **,** **M** ^**−1**^	***n***
**7a**	5655.45	0.35	0.96
**7b**	35,237.51	3.56	1.00
**7c**	52,974.61	26.90	1.15
**7d**	45,150.17	19.38	1.14
**7e**	41,174.83	21.93	1.16
**9a**	59,854.34	4.16	0.97
**9b**	63,696.32	0.79	1.45
**9c**	68,338.33	27.52	1.14
**9d**	63,187.02	0.44	0.76
**9e**	75,082.69	216.12	1.32

**FIGURE 6:**
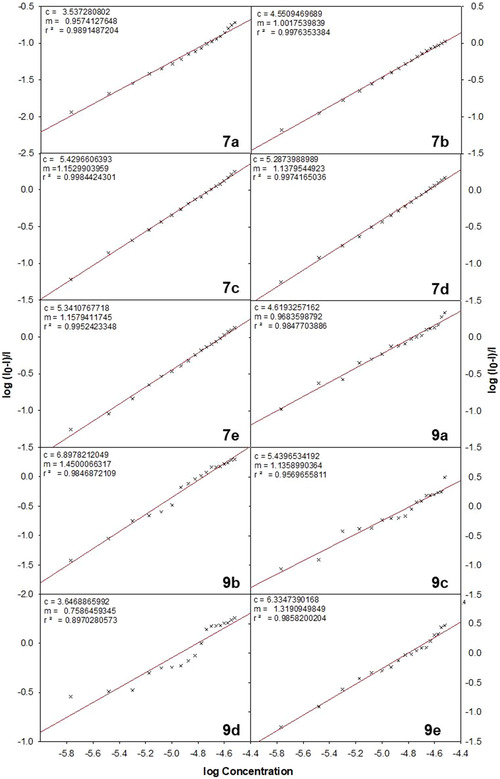
Scatchard plot to depict the equilibrium constant for the interaction of various diarylmethane derivatives **7a–e** and **9a–e** with BSA protein.

Also, with the help of Scatchard equation as given below, the equilibrium binding constants were evaluated by assessing their ability to bind to more than one equivalent sites on the protein.

$$\log[(I_{0}‐I)/I]=\log K_{b}+n\log[\mathrm{DAM}]$$

where *K*_b_ is the equilibrium binding constant for **7a–e** and **9a–e** with BSA and *n* is the number of equivalent binding sites on the protein. In Figure 6, the number of binding sites (*n*) and the binding constant (*K*_b_) values were calculated from the plot of log [(*I*_0_− *I*)/*I*] versus log [DAM]. The binding constant and number of binding sites are given in Table 5. The data revealed that compound **9e** exhibits the highest binding affinity to BSA, with a *K*_b_ of 2.16 × 10^6^ M^−1^, markedly exceeding typical small‐molecule binders. Compounds **7c, 7d, 7e**, and **9c** also showed moderate to strong binding, which is likely due to favorable structural features such as naphthyl and methoxy groups. In contrast, **7a** display weak binding, indicating less effective interaction with BSA, while **9b** and **9d**, despite high quenching constants, exhibited poor binding affinity, suggesting the involvement of dynamic quenching mechanisms rather than static binding.

**TABLE 5:** Evaluation of Stern–Volmer quenching constant and equilibrium binding constant for 7a–e and 9a–e on ct‐DNA.

Entry	***K***_**SV**_ **, M** ^**−1**^	***K***_**b**_ ** × 10** ^**4**^ **, M** ^**−1**^
**7a**	216.53	8.49
**7b**	170.00	49.06
**7c**	1538.64	—
**7d**	1298.75	1.96
**7e**	420.95	81.11
**9a**	949.99	118.56
**9b**	821.50	288.70
**9c**	1183.44	40.9
**9d**	510.93	547.11
**9e**	184.96	29.26

Binding analysis suggests that most compounds interact with a single site on BSA. Notably, **9e** is the most efficient quencher, consistent with its highest binding affinity. A clear correlation emerges between higher binding constants (*K*_b_) and increased Stern–Volmer quenching constants (*K*_sv_), indicating that quenching primarily occurs via static mechanisms. This involves complex formation and/or Förster resonance energy transfer (FRET), where stronger binding enhances energy transfer and alters the fluorophore environment.

The proximity of the bound compounds to the tryptophan residue in BSA facilitates FRET, leading to energy transfer from BSA to the compounds and subsequent emission from the latter. This confirms that stronger binding affinities promote more effective quenching through static interactions and energy transfer processes.

##### 3.2.1.2 DNA Binding

The ability of **7a–e** and **9a–e** to interact with nucleic acids was evaluated from their absorption spectral titration with ct‐DNA in 10 mM Tris‐HCl buffer solution (at pH 7.2). The purity of ct‐DNA was ensured by monitoring the absorbance ratio at 260 and 280 nm (*A*_260_/*A*_280_), and the absence of any protein contamination was ascertained by the above value of ∼1.8. With the known value of molar extinction coefficient (6600 M^−1^) at 260 nm for ct‐DNA, the concentration of DNA per nucleotide was calculated. Methanol was used to prepare the stock solutions of **7a–e** and **9a–e**, and 10 mM Tris‐HCl buffer solution (pH 7.2) was used for further dilutions. The binding evaluations of **7a–e** and **9a–e** were performed at a fixed concentration (∼5 µM) along with increasing amounts of ct‐DNA (0–30 µM). The binding constants (*K*_b_) were calculated by using the following equation:

$$\left[\mathrm{DNA}]/(\varepsilon_{a}‐\varepsilon_{f})=[\mathrm{DNA}]/(\varepsilon_{b}‐\varepsilon_{f})+1/K_{b}(\varepsilon_{b}‐\varepsilon_{f}\right)$$

where [DNA] represents the concentration of the DNA in the base pairs, *ε*_a_ is the apparent absorption coefficients as obtained by calculating *A*_obsd_/[complex], and *ε*_f_ and *ε*_b_ correspond to the extinction coefficient of the complex in free form and the extinction coefficient of fully DNA‐bound forms of the complex, respectively. The ratio of slope to *y*‐intercept provides the value of intrinsic binding constant (*K*_b_).

Further experiments were carried out to assess the competitive binding ability of DAMs and ethidium bromide (EtBr) for ct‐DNA. EtBr is a well‐known cationic dye possessing a phenanthridine ring and is widely employed as a responsive fluorescence probe for the detection of nucleic acids. The intense fluorescence originating from EtBr is due to the interaction between EtBr and adjacent DNA base pairs.

**FIGURE 7:**
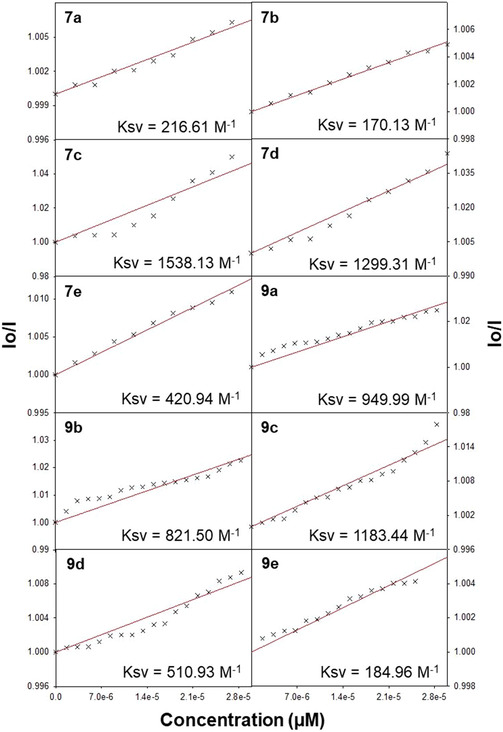
The graph depicting the Stern–Volmer plot for the competitive binding of various diarylmethane derivatives (**7a–e** and **9a–e**) with ct‐DNA.

Five micromolar each of EtBr and ct‐DNA was taken in 10 mM Tris‐HCl buffer solution (pH = 7.2) and incubated at ambient temperature for about an hour in the presence and absence of various concentrations of DAMs (0–30 µM). The excitation and emission wavelengths were chosen as 515 and 597 nm, respectively. None of the DAMs showed any intrinsic fluorescence emission at ∼597 nm in presence or absence of ct‐DNA at room temperature. But the EtBr bound to ct‐DNA showed intense fluorescence emission, which is expected to decrease upon its displacement with various DAMs (quencher). The Stern–Volmer equation was used to investigate the quenching constants of DAMs. The quenching plots of EtBr bound to ct‐DNA upon titrating with different concentrations of DAMs are depicted in Figure 7.

**FIGURE 8:**
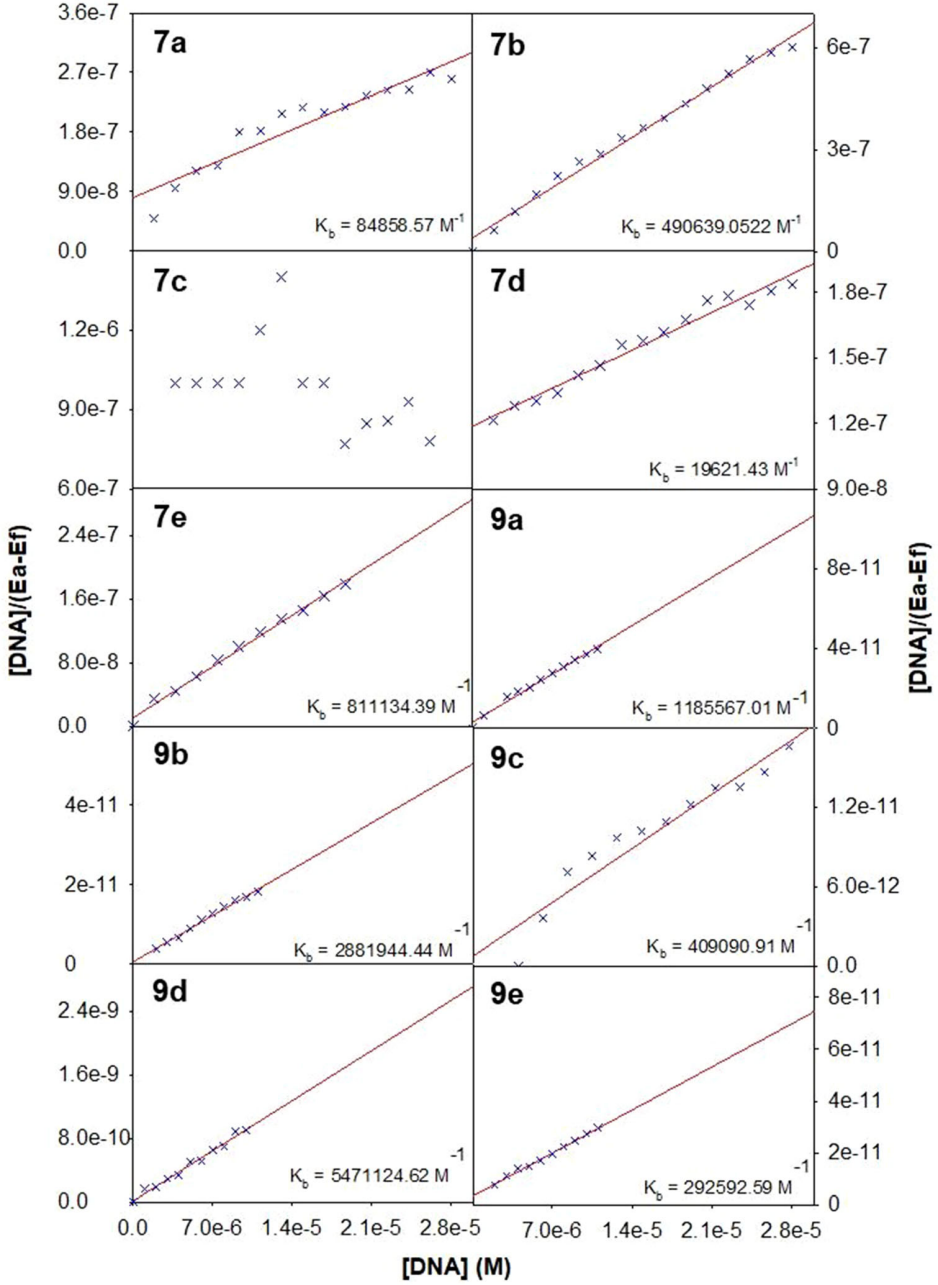
Plots of [DNA]/(*ε*_a_ – *ε*_f_) versus [DNA] for the titration of the **7a–e** and **9a–e** compounds with ct‐DNA.

The binding constant for the interaction of various DAMs and ct‐DNA was further determined by following the spectrophotometric method. The data obtained was then analyzed using the Scatchard equation, and the resulting plots are presented in Figure 8 and the relevant data points are compiled in Table 5. Compound **9d** displays the highest equilibrium binding constant (*K*_b_ = 5.47 × 10^5^ M^−1^), indicating strong binding with ct‐DNA, while moderate values were observed for **9a** and **9b** and a poor value was displayed for **7d**, while compound **7c** shows high fluorescence quenching but lacks a measurable binding constant, suggesting possible nonspecific interactions or dynamic quenching without the formation of a stable complex.

Interestingly, for **7d** and **9c**, high *K*_sv_ but a low *K*_b_ were observed, and similarly, **7b** and **9e** revealed a moderate *K*_b_ but low *K*_sv_ values, suggesting that these compounds bind to DNA following specific binding modes but differ from typical EtBr intercalation, or they might be binding to different sites.

### 3.3 Ability to Inhibit PARP1—Evaluation of Anticancer Potential

To understand the nature and mechanism of interactions between various **7a–e and 9a–e** derivatives with PARP1 (PDB Id. 5HA9) protein, docking investigations were carried out. The binding affinities for each of the derivatives were calculated using the GLIDE module of Schrödinger. The aforementioned data was utilized to precisely predict the binding configurations and interactions between the protein and ligands using the ligand docking technique based on the grid assignment [40] that was predicted by selecting the protein target's (PARP1) binding site residues [41]. The proposed **7a–e** and **9a–e** derivatives were assessed for their ability to inhibit PARP1 using the above strategic approaches, considering both docking scores and binding energies of each compound. The binding interactions pertaining to electrostatic forces, van der Waals contacts, hydrogen bonds, and lipophilic forces were displayed in their docked conformations by utilizing the BIOVIA DSV tool.

Further refinement of the above docked structures was carried out following the MM‐GBSA refinement processes with a flexible residue distance of 5 Å to evaluate the overall binding free energy. Subsequently, the previously docked outcomes were then rescored and validated after eliminating the false positives. The ranking and grading of each small molecule's binding postures were determined using the standard precision (SP) G‐score. The Schrödinger suite MM‐GBSA module was utilized to calculate the compounds’ total binding free energy. To evaluate the binding free energies, the pose viewer files of the reference compound as well as a variety of hit molecules from glide docking assessments were utilized. The calculation of binding free energy was performed using the following equation which incorporates factors such as van der Waals interactions, electrostatic interactions, entropy terms, and polar and nonpolar contributions of ligands.

$$\Delta G=\Sigma (\Delta G_{\mathrm{Bind}\;\mathrm{coulomb}}+\Delta G_{\mathrm{Bind}\;\mathrm{covalent}}+\Delta G_{\mathrm{Bind}\;H‐\mathrm{bond}}+\Delta G_{\mathrm{Bind}\;\mathrm{Lipo}}+\Delta G_{\mathrm{Bindpacking}}+\Delta G_{\mathrm{Bind}\;\mathrm{Self}\mathrm{cont}}+\Delta G_{\mathrm{Bind}\;\mathrm{Solv}\;\mathrm{GB}}+\Delta G_{\mathrm{Bind}\;\mathrm{vdw}})$$

Thus, calculated binding free energies were used to assess the interactions between the biological macromolecules like proteins that approach small‐molecule ligands. The following equation was estimated for the MM/GBSA computations. To gain further insight, the binding free energies were calculated using MM/GBSA method, as described by the following equation.

(1) $$\Delta G_{\mathrm{bind}}=\Delta E_{\mathrm{MM}}+\Delta G_{\mathrm{solv}}+\Delta G_{\mathrm{SA}}$$

where Δ*E*_MM_ represents the energy difference between the PARP1‐ligand complex and the combined energies of PARP1 alone (without any ligand) and liganded PARP1. The computation of the change in free solvation energy is based on the variation in energy difference between the complex of PARP1 and the ligand and the total energy of both the PARP1 protein unbound to ligand and PARP1 complex bound to ligand. This is represented as Δ*G*_solv_, whereas Δ*G*_SA_ indicates the changes in free energy caused by surface area variations in the PARP1‐ligand complex and the total energies of the PARP1 protein alone (without any ligand) and ligand‐bound PARP1 complexes.

### 3.4 In Silico Investigation of DAM Derivatives Binding to the Biomolecular Target PARP1

DAM is emerging as a high‐value candidate in drug discovery with demonstrated anticancer, anti‐inflammatory, and anti‐infectious activities. They also serve as important agents in numerous therapeutic applications. Tamoxifen is a well‐known DAM that has proven its therapeutic potential for hormone‐dependent breast cancer. Moreover, these compounds have also displayed low cytotoxicity projecting them as promising candidates for numerous pharmaceutical applications. Although the mechanisms of action of many of the reported compounds are known, there are several drugs with excellent potentials whose functional mechanisms are yet to be investigated. Herein, the potential of DAM derivatives as anticancer agents was screened with the help of various computational methods against the target PARP1 (5HA9), a known target for TNBC. The key residues that are present in the binding pocket of 5HA9 include hydrophobic residues like *Y28*, *L108*, *A219*, *Y228*, *M229*, *Y235*, *A237*, and *Y246*, polar residues like *E102*, *N106*, and *S203* and electrically charged residues like *D105*, *D109*, *H201*, *R217*, and *H248*. The above residues were considered for all the investigations and evaluations carried out in this study. The DAM derivatives presented here were examined for interactions with the binding pocket of PARP1 using docking calculations with Schrodinger suite. The docking scores and interacting residues which are compiled in Tables 6 and 7, respectively, were used to determine the binding efficiency. The best docking scores of −8.487 and −7.445 kcal mol^−1^ were obtained for **7d** and **7b** respectively, while other derivatives also revealed a significant value. It can be observed that in all the cases, the contribution toward hydrophobic interactions from the aliphatic ring substitutions are almost similar and the observed differences arise from the aromatic substitutions. In the case of **7d**, the naphthyl moiety stabilizes the interaction through pi–pi stacking. The methoxy phenyl substitution stabilizes the interaction in the case of **7b** and **7c**, while in the latter, the steric factor arising from the benzyl substitution destabilizes.

**TABLE 6:** Energy parameters (kcal mol^−1^) as evaluated from docking interactions for various diarylmethanes.

Compounds	G‐score	**Δ*G***_**Bind**_	**Δ*G***_**Bind**_ **Coulomb**	**Δ*G***_**Bind**_ **covalent**	**Δ*G***_**Bind**_ **Lipo**	**Δ*G***_**Bind**_ **Solv GB**	**Δ*G***_**Bind**_ **vdW**	Lig strain energy
**7a**	−6.793	−24.55	−78.24	15.34	−31.71	107.77	−36.55	21.568
**7b**	−7.445	−38.09	−50.67	13.09	−33.54	77.19	−42.71	22.386
**7c**	−6.907	−22.82	−78.74	16.43	−36.98	123.69	−45.99	23.113
**7d**	−8.487	−27.64	−55.08	13.25	−38.19	86.79	−33.45	22.334
**7e**	−5.834	−9.71	−72.19	14.17	−38.35	102.76	−15.60	27.367
**9a**	−6.050	−17.06	2.77	2.40	−35.70	65.38	−45.37	12.105
**9b**	−5.210	4.72	−2.58	15.15	−36.39	61.88	−28.02	25.313
**9c**	−5.714	−7.42	−2.65	14.13	−39.23	67.25	−40.83	22.309
**9d**	−4.277	20.8	−31.27	11.7	−21.56	98.24	−36.24	13.064
**9e**	−6.328	−4.04	1.10	10.59	−38.79	70.96	−39.86	20.719
Tamoxifen	−6.194	−41.97	−53.58	2.98	−32.91	93.54	−51.12	3.901

**TABLE 7:** Interacting with amino acids in the binding pocket of PARP1 (5HA9).

Entry	Interacting amino acids
**7a**	*E102*, *D105*, *H201*, *Y235*, *Y246*
**7b**	*Y28*, *D105*, *N106*, *H201*, *A219*, *Y228*, *G233*, *Y246*, *H248*
**7c**	*E102*, *D105*, *L108*, *D109*, *S203*, *R217*, *M229*, *Y235*, *A237*, *Y246*
**7d**	*D105*, *N106*, *D109*, *H201*, *S203*, *A219*, *Y228*, *Y246*, *H248*
**7e**	*D105*, *D109*, *H201*, *A219*, *Y228*, *Y235*, *T246*
**9a**	*E102*, *H201*, *Y228*, *M229*, *A237*, *Y235*, *Y246*, *E327*
**9b**	*E102*, *D105*, *H201*, *A219*, *Y228*, *Y235*, *Y246*
**9c**	*E102*, *D105*, *H201*, *A219*, *Y228*, *M229*, *Y235*, *Y246*, *E327*
**9d**	*E102*, *H201*, *M229*, *Y235*
**9e**	*D105*, *Y228*, *M229*, *Y235*, *A237*, *Y246*

Based on the docking score, compounds **7d** and **7b** were identified as best candidates to bind to the pocket residues of PARP1. But based on the binding free energy, **7b** was identified as the best compound and has displayed effective interactions with PARP1. The binding free energies of **7a–e** and **9a–e** were −24.55, −38.09, −22.82, −27.64, −9.71, −17.06, −4.72, −7.42, −20.8, and −4.04 kcal mol^−1^, respectively, while that for tamoxifen was −41.97 kcal mol^−1^. It could be observed that **7b** and **7d** display good docking score and the lower positive values of generalized Born electrostatic solvation energy stabilize the overall binding interactions. The interacting residues for each of the derivatives are compiled in Table 7. The binding postures for all the compounds with the binding cavity of PARP1, as deduced from docking results, are depicted in Figure S11. The percentage contributions of various stabilizing interactions are given in Figure S12.

### 3.5 Molecular Dynamic Simulations

#### 3.5.1 Evaluation of RMSD Upon Interaction With PARP1

**FIGURE 9:**
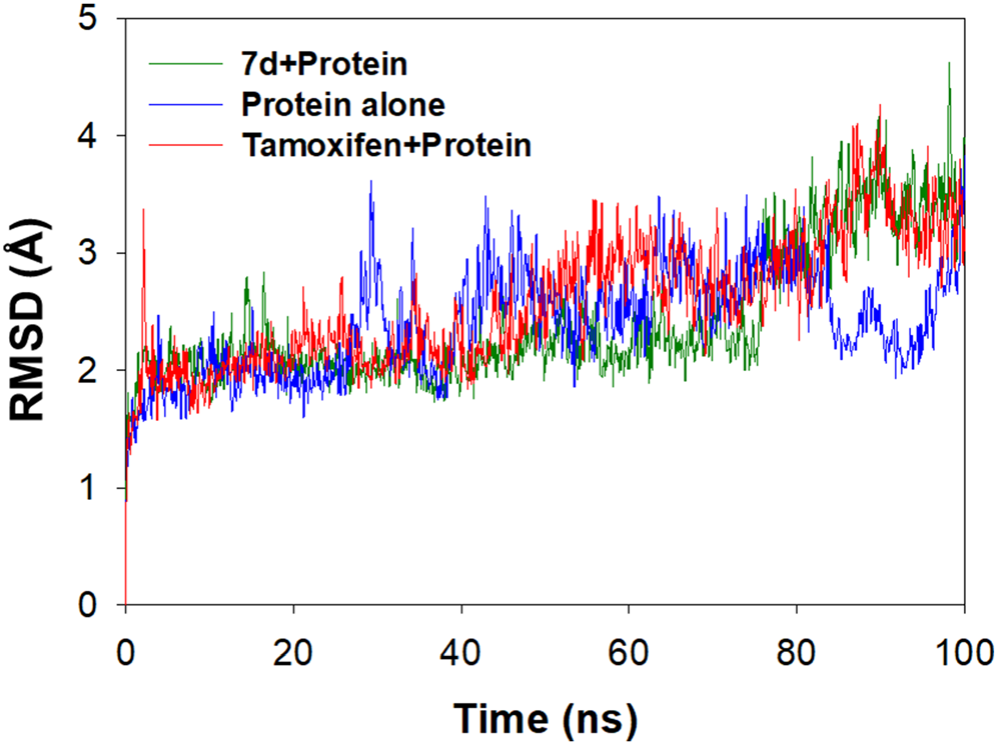
The plot of RMSD trajectories for PARP1 in the absence (blue) and presence (green) of **7d** and tamoxifen.

Root mean square deviation (RMSD) is a parameter that measures the changes induced in the structural conformations of protein backbone during the simulation period of 100 ns. The root mean square deviation will be assessed to quantify the induced alterations in the structural conformations of the protein backbone during the 100 ns simulation period. It can be inferred that the system under study is more stable and that the interactions do not significantly alter the protein's structure as observed from its negligible deviation in RMSD trajectory. Based on the above docking results, **7d** was chosen for further investigation through molecular dynamic interactions. The RMSD trajectories of PARP1 with and without ligand **7d** are shown in Figure 9, and they are further compared with those of the well‐known inhibitor tamoxifen.

Figure 9 reveals a remarkably stable trajectory for **7d** with RMSD of 2.42 ± 1.59 Å when examined during the simulation period of 100 ns though an insignificant fluctuation was noticed in after 80 ns. This indicates that the interaction of **7d** with the binding cavity of PARP1 does not have significant impact on its stability or structure. Moreover, a negligible deviation of ∼2.40 ± 1.32 Å for the protein in the absence of any ligand. Tamoxifen also showed a minimal variation up to 100 ns, measuring RMSD of ∼2.57 ± 1.49 Å.

**FIGURE 10:**
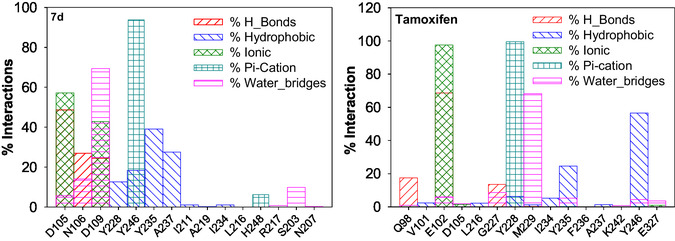
Bar graph depicting the extent (%) of various interactions of binding pocket residues with **7d** (left) and tamoxifen (right).

The observed stabilization may result from the formation multiple interactions such as hydrogen bonding, hydrophobic, π‐cation, water bridges, etc. Overall, the average value of RMSD for **7d** indicates stable interactions with the binding pocket of PARP1. The following active site residues *D105*, *N106*, and *D109* were involved in hydrogen‐bonding interactions, while residues *D105*, *N106*, *D109*, *S203*, *N207*, *I211*, *L216*, *R217*, *A219*, *Y228*, *I234*, *Y235*, *A237*, *Y246*, and *H248* contribute toward hydrophobic interactions. In addition to the above, residues like *Y246* and *H248* were also involved in π‐ cation interactions, while *D105*, *N106*, *D109*, *S203*, *N207*, and *R217* residues were also involved in the formation of water bridges (Figure 10). In the case of tamoxifen, though hydrophobic and hydrogen‐bonding interactions stabilize its binding to the cavity, a significant contribution toward stabilization was also obtained from water bridges that connects the binding site residues and the ligand atoms through hydrogen bonds.

#### 3.5.2 Principal Component Analysis

**FIGURE 11:**
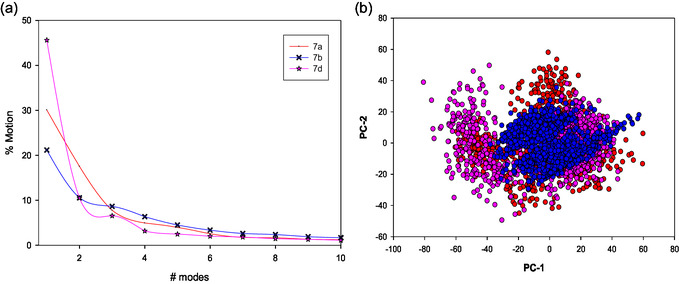
Plot of (a) Mode vs. % Motion (b) PC1 vs. PC2 as evaluated by postmolecular dynamic conformational and quantitative analysis of **7a** (red), **7b** (blue), and **7d** (magenta) upon its interaction with PARP1.

Further to reduce the dimensionality of the above trajectories of the protein–ligand complexes and also to identify the predominant motions that contribute to the structural variations, principal component analyses were carried out. As seen in Figure 11a, the major attributions pertaining to the motions in the protein upon its interaction with **7a**, **7b**, and **7d** are contained in the first two modes. Moreover, the overall movement of PARP1 in the complex during its interaction with **7a**, **7b**, and **7d** is depicted in Figure 11b as the plot of principal component 1 (PC1) vs. 2 (PC2) where the symbols in the plot represent the correlation motion of the C alpha atoms in the amino acid residues. A PCA scatter plot showing that the points are broadly distributed but overlap significantly indicates they can occupy similar conformational states. The significant overlap suggests that the ligands do not fundamentally restrict the protein's overall essential dynamics but rather stabilize a functionally relevant ensemble of conformations.

Specifically, the density of the points for each compound, particularly the large, central cluster, indicates that each complex consistently occupies a major structural state during the simulation time. In the case of **7b**, the clusters are more centered near (0, 0) of the plot as compared to **7a** and **7d**, indicating that the former has a more rigid, well‐defined conformational state to interact strongly with the protein. Also, the distribution confirms that **7a**, **7b**, and **7d** are all stable binders that effectively engage the protein, leading to robust conformational sampling along the most significant degrees of freedom.

**FIGURE 12:**
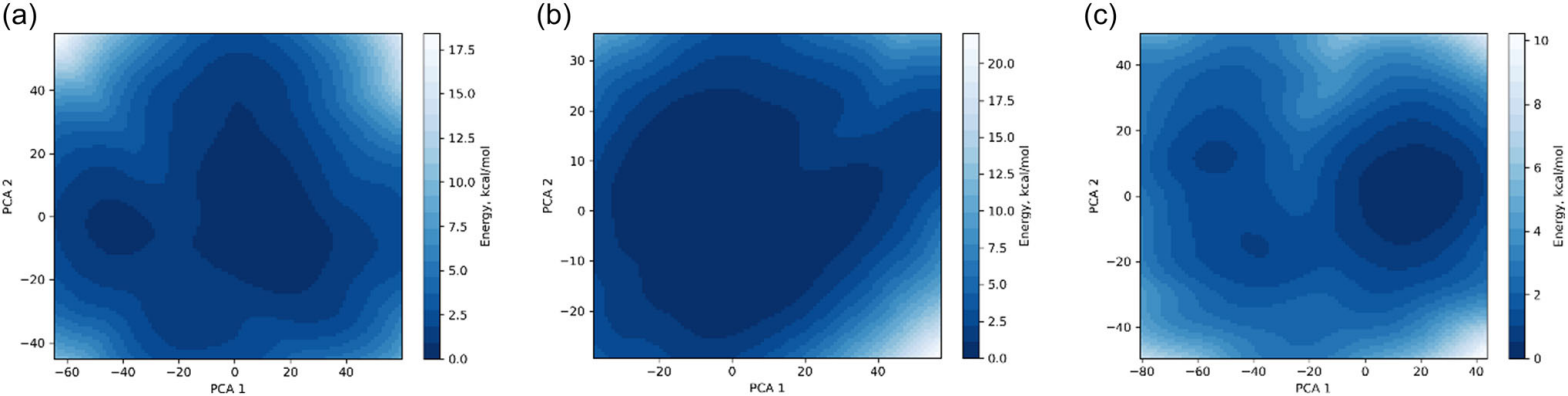
The contour plot depicting the free energy landscape projection for **7a** (a), **7b** (b), and **7d** (c) upon its interaction with 5HA9.

In general, the free energy landscape represents the relative stability of different conformational states and function of any biological macromolecule like proteins and nucleic acid and provides qualitative information on the number of free energy minima. Also, it gives information about the interactions between a complex target molecule (5HA9) and other small molecular drugs (**7a**, **7b**, and **7d**) on their binding affinities. As can be seen in Figure 12, **7b** displays a large, deep, single basin centered around (PC1=−40 to 20 and PC2=−20 to 20) with relative energies less than 2.5 kcal/mol, suggesting a single dominant stable conformation. However, compound **7a** shows that two distinct basins suggest moderate flexibility and the adoption of two well‐separated structural groups during the simulation. For **7d**, the three distinct free energy minima exhibit the highest conformational heterogeneity, sampling at least three well‐separated metastable states. This indicates **7d** is accommodated by the protein in multiple, distinct, and thermodynamically favorable binding modes. **7b** displays more localized energy minima as compared to **7a** and **7d**, while **7d** engages the binding site via multiple thermodynamically favorable binding poses. This further indicates a stronger interaction between **7b** and **7d** with 5HA9.

**FIGURE 13:**
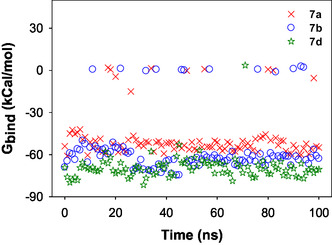
The plot depicting the variation in binding free energy for **7a**, **7b**, and **7d** upon its interaction with PARP1 as evaluated for a simulation period of 100 ns.

Figure 13 shows the variation of free energies with respect to time. The plot helps to validate the results obtained from molecular dynamic simulations performed over the interaction of **7a**, **7b**, and **7d** with 5HA9. The average binding free energy for **7a**, **7b**, and **7d** are −48.17 ± 24, −55.33 ± 28, and −69.26 ± 7 kcal mol^−1^, respectively, with consistent clustering around −60 and −80 kcal mol^−1^. The results revealed that these derivatives displayed the highest degree of temporal fluctuations.

#### 3.5.3 Evaluation of RMSF Upon Interaction of 4 With PARP1

**FIGURE 14:**
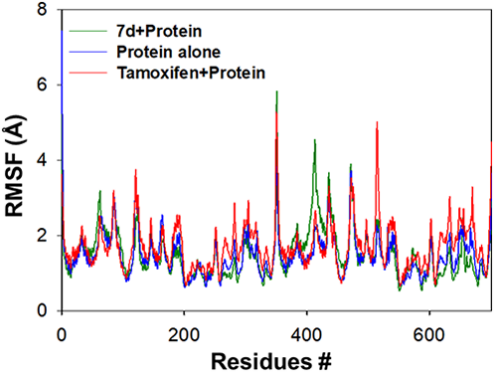
RMSF trajectories of PARP1 in the absence (blue) and presence (green) of **7d** and tamoxifen (red).

By following the RMSF trajectories, it is possible to monitor the variations induced in the amino acid residues of the protein as it interacts with various ligands. In this case, the positional variations of each PARP1 residue during the interaction with **7d** and tamoxifen were monitored. The RMSF trajectories of **7d** and tamoxifen are displayed in Figure 14.

During the analysis period of 100 ns, the following residues were found to be in contact with the ligands and showed deviations >4 Å—*M351*, *K352*, *G413*, and *S414*. The above residues were confined to the binding cavity, and as the ligand approaches, the induced fluctuations were enhanced for its accommodation. In the absence of any ligand, insignificant fluctuations with an average RMSF of 1.71 ± 2.25 Å were observed for all the residues. The average fluctuation for **7d**, observed during the interaction period, was found to be 1.49 ± 2.66 Å. In the case of tamoxifen, the residues *T350*, *M351*, *K352*, *T514*, *T515*, and *T701* revealed fluctuations >4 Å, and most of the ligand‐interacting residues were localized inside or close to the binding pocket. The average fluctuation observed for tamoxifen was found to be 1.72 ± 2.25 Å.

#### 3.5.4 Evaluation of Rg Upon Interaction of 4 With PARP1

**FIGURE 15:**
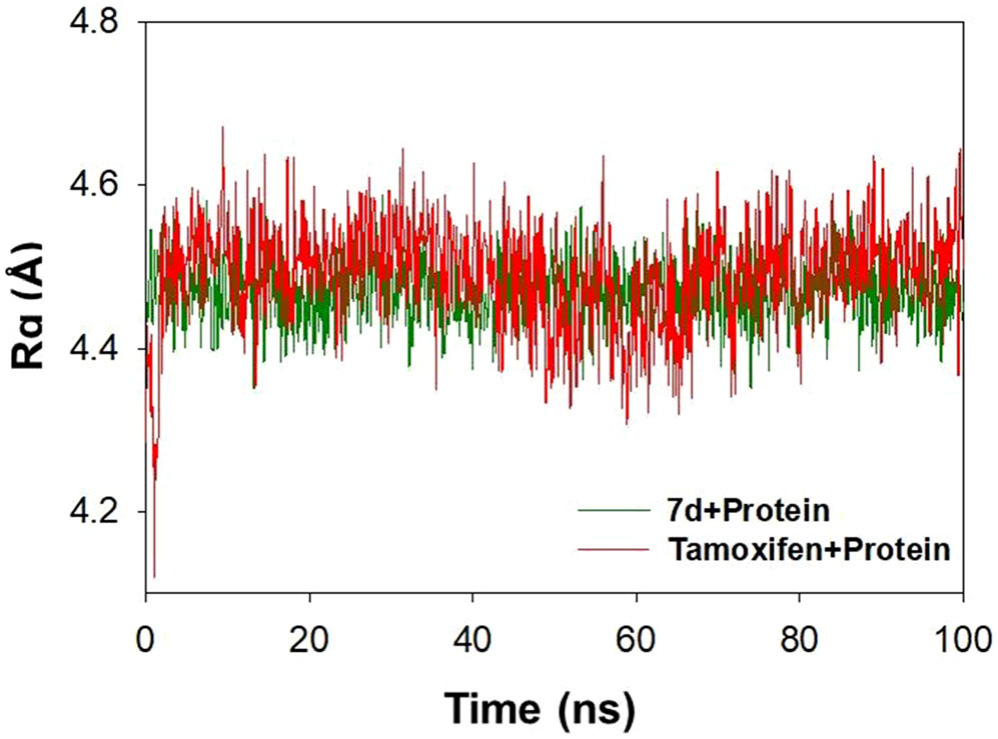
The trajectory showing the PARP1's radius of gyration in relation to **7d** (red) and tamoxifen (green).

The radius of gyration was observed over a 100 ns interaction time to evaluate the overall structural compactness and rigidity of the protein as it interacts with various ligands. Rg was determined by measuring the distance between the ligand's central axis and the atom's rotational position with the maximal energy. It is also known that changes in Rg values result from ligand‐induced conformational adjustments as they approach the target site. In this study, we analyzed the Rg of **7d** and tamoxifen, examining how ligands impact the protein's compactness. Figure 15 illustrates the variation in the radius of gyration (Rg) overtime, potentially influenced by the size and structure of the ligand molecules compared to the reference drug tamoxifen.

It could be noted that deviations in the trajectory of **7d** and tamoxifen were comparable with insignificant deviations. This indicates a stable binding interaction exists between 4 and the target protein. The average Rg observed for **7d** is 4.47 ± 0.12, while that of tamoxifen is 4.49 ± 0.28 Å.

#### 3.5.5 Evaluation of SASA Upon Interaction of 4 With PARP1

**FIGURE 16:**
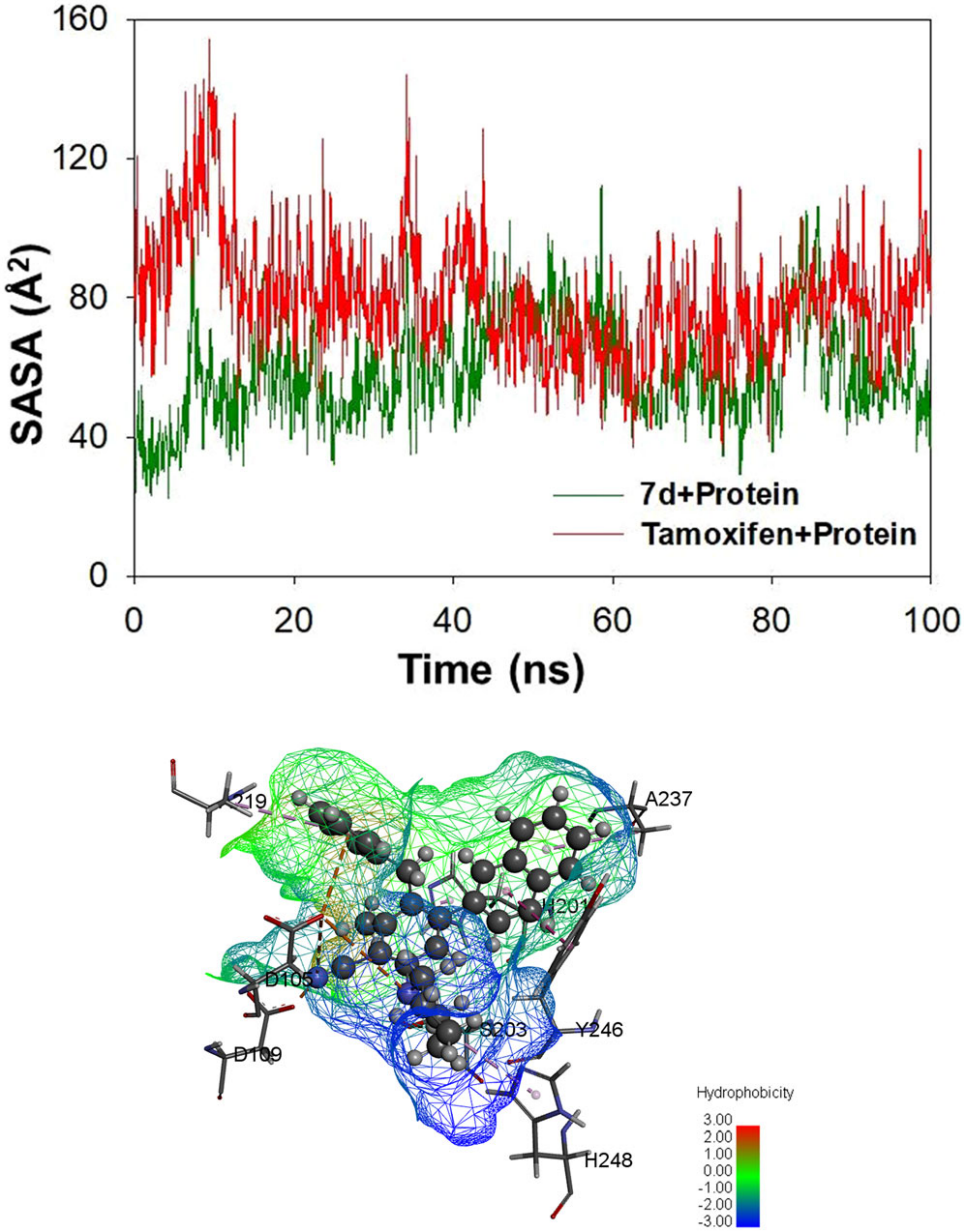
Upper panel: the trajectory depicting the variation in solvent accessible surface area during 100 ns simulation period for **7d** and tamoxifen. Lower panel: contour depicting the hydrophobic surface of **7d** within the binding cavity.

The nature of polar and nonpolar surface of the ligand is greatly influenced by the interaction of ligands with PARP1. The above investigation will help to identify the key binding pocket residues on the protein target and their ease of access by the solvent molecules. The trajectory depicting the variation in SASA for **7d** and tamoxifen is shown in Figure 16. During a 100 ns simulation period, **7d** displayed negligible fluctuations, indicating that its surface accessibility remains mostly unchanged when interacting with PARP1. The contour depicting the solvent accessible surface (SAS) when the **7d** is localized within the binding cavity of PARP1 is shown in the lower panel of Figure 16. It is evident that there is a good correlation between the SAS and water bridges discussed earlier. In the case of tamoxifen, being relatively smaller than **7d**, the SAS is expected to be relatively smaller. Therefore, it is expected that **7d** should have been more stabilized through solvent‐induced hydrogen‐bonding interactions through the formation of water bridges. The data obtained from SASA evaluations indicate that solvent plays crucial role in stabilizing the ligands toward binding to the cavity.

#### 3.5.6 Hydrogen Bonds

**FIGURE 17:**
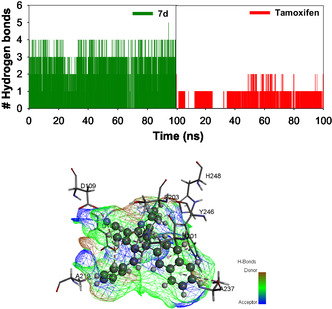
Top panel: bar graph showing the number of hydrogen bonds formed by **7d** and tamoxifen made with PARP1 protein over the course of the 100 ns simulation. Bottom panel: the contour surface illustrating the hydrogen‐bonding capability of **7d**.

It is well known that biomolecules use a variety of interactions to preserve their structural integrity and stability. In this analysis, we have explored the impact of ligand interactions on the stability of PARP1 through hydrogen bonds during a 100 ns time frame, recognizing the significance of hydrogen bonds as one of the key components in stabilizing protein–ligand interactions. The results of the MD simulation are depicted in Figure 17 (top panel).

**FIGURE 18:**
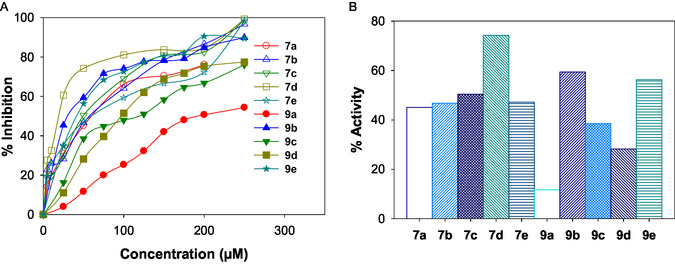
(A) The plot depicting the % inhibition of protein denaturation at different concentrations of various **7a–e** and **9a–e**. (B) Bar graph depicting the % activity at 50 μM concentration of various DAMs.

It could be seen from Figure 18 that unlike tamoxifen, the influence of hydrogen bonds toward stabilizing the interactions for **7d** was significantly high. A maximum of five hydrogen bonds were observed with *D105*, *N106*, and *D109*, and the same residues were involved in hydrogen bond formation at multiple time points during simulation period. Similarly, in the case of tamoxifen, residues including *M229*, *G227*, *E102*, and *Q980* were involved in the formation of two hydrogen bonds during the simulation period.

### 3.6 Anti‐Inflammatory Activity

A slightly modified version of the Chandra et al. (2012) approach was used to examine the compounds’ in vitro anti‐inflammatory efficacy. A solution of 10 μM bovine serum albumin in 1× PBS, pH 7.2, was incubated at 37°C for 15 min with increasing concentrations (0–200 μM) of **7a–e** and **9a–e**. Subsequently, these mixtures were incubated in a water bath at 70°C for 5 min. The absorbance at 660 nm was then measured after cooling. Percentage inhibition of protein denaturation was calculated by assuming 100% denaturation in the control group. Figure 18A illustrates the variation in % inhibition at various concentrations of **7a–e** and **9a–e**, while the bar graph in Figure 18B displays the % inhibitory activity at 50 µM.

%inhibition=100*((Absorbance of test sample/Absorbance of control sample)−1)

Inflammation results from the denaturation of intracellular materials and protein components in cells, which is further connected to tissue damage. Consequently, it is inferred that a compounds ability to prevent protein denaturation indicates anti‐inflammatory potential. The denatured proteins play important roles as autoantigens and are the primary cause for numerous auto‐immune diseases. The assay results revealed that all the compounds exhibited anti‐inflammatory activity by significantly inhibiting protein denaturation. In Figure 18A, all derivatives exhibited a progressive increase in inhibition with rising concentration, indicating a dose‐dependent response. Among the tested compounds, **7d** demonstrated the most pronounced inhibitory effect across the entire concentration range, followed by **9b** and **9e**. Figure 18B depicts a clear comparison at 50 μM, where **7d** recorded the highest percentage inhibition, establishing it as the most potent anti‐inflammatory agent at this concentration. The remaining derivatives showed relatively lower inhibitory effects, suggesting limited anti‐inflammatory potential at this concentration.

## 4 Prediction of Anticancer Activity—Prediction of Activity Spectra for Substance (PASS) Algorithm

The antineoplastic effects of **7a–e** and **9a–e** were predicted using the online PASS method. The program divides the molecule into actives (Pa) and inactives (Pi) based on their biological activity profile. If the compound's Pa value is higher than its Pi, there's a good chance it will show signs of experimental activity. Conversely, when Pi exceeds Pa, the compounds are thought to be less active. A thorough explanation of every computational technique, including the PASS and QikProp algorithms, has been documented elsewhere [42].

The anticancer efficacies of **7a–e** and **9a–e** were assessed further by employing the above prediction algorithm. It could be noted that most of the compounds have greater active scores, further suggesting the likelihood of them to exhibit greater experimental activity. Also, all the compounds exhibited strong inhibitory properties based on their antineoplastic activity against different types of cancer. As shown in Table 8, it is found that all the compounds have a good chance of acting as effective anticancer drug. Based on the data, there is a higher likelihood that these compounds will demonstrate experimental activity.

**TABLE 8:** Anticancer activity as predicted by PASS online algorithm.

Compounds	Pa	Pi	Activity
**7a**	0.259	0.023	Antineoplastic (bone cancer)
	0.308	0.213	Antineoplastic (non‐Hodgkin's lymphoma)
**7b**	0.460	0.058	Antineoplastic (non‐Hodgkin's lymphoma)
	0.228	0.069	Antineoplastic alkaloid
	0.192	0.039	Antineoplastic (thyroid cancer)
**7c**	0.480	0.046	Antineoplastic (non‐Hodgkin's lymphoma)
	0.190	0.042	Antineoplastic (thyroid cancer)
	0.204	0.090	Antineoplastic alkaloid
**7d**	0.412	0.097	Antineoplastic (non‐Hodgkin's lymphoma)
	0.167	0.147	Antineoplastic alkaloid
**7e**	0.249	0.034	Antineoplastic (bone cancer)
**9a**	0.178	0.126	Antineoplastic alkaloid
	0.149	0.102	Antineoplastic (melanoma)
**9b**	0.164	0.083	Antineoplastic (melanoma)
	0.186	0.113	Antineoplastic alkaloid
**9c**	0.200	0.095	Antineoplastic alkaloid
	0.161	0.087	Antineoplastic (melanoma)
**9d**	0.277	0.027	Antineoplastic (melanoma)
	0.209	0.085	Antineoplastic alkaloid
**9e**	0.178	0.126	Antineoplastic alkaloid
	0.149	0.102	Antineoplastic (melanoma)
Tamoxifen	0.173	0.026	Antineoplastic, alkylator
	0.173	0.067	Antineoplastic (thyroid cancer)
	0.213	0.114	Antineoplastic (bone cancer)
	0.304	0.218	Antineoplastic (non‐Hodgkin's lymphoma)
	0.207	0.175	Antineoplastic (small cell lung cancer)
	0.153	0.128	Antineoplastic (endocrine cancer)

## 5 *Pharmacokinetic Property Evaluation* (A*ADME*)

In the process of drug discovery, a large amount of information can be obtained from pharmacokinetic parameters like absorption, distribution, metabolism, and excretion (ADME). Absorption is the process by which a drug is transferred from the site of administration to the intended target, where its action is anticipated to be carried out. There are several routes to administer the drug—oral, arterial, intramuscular, intrapulmonary, subcutaneous, percutaneous, etc. The factors that influence the process of absorption are surface area, permeability, inhibition potential, etc. Thus, absorbed drug needs to be dispersed across different tissues and the process is governed by the factor such as permeability, lipophilicity, molecular size, ionization constant, and protein affinity, etc. If the metabolism rate is very high, optimization might be required to observe lower bioavailability and higher clearance to minimize various associated side effects. Many times, the drug molecules cross‐react among themselves to result in unexpected modifications in their structures. Therefore, it is very much necessary to investigate the process of drug metabolism before proceeding further in the process of drug discovery. Ultimately, the absorbed drug needs to be excreted from the body via the process known as total clearance.

The drug‐like potential of DAM derivatives was assessed using the QikProp module of the Schrodinger suite, which includes features such as ADME, as well as key descriptors like stars, HOA, and Lipinski's rule of five [43]. Any violation of the descriptors was obtained by considering their deviation below 95% of the values for known inhibitors were represented as star values. Moreover, for a molecule to act as a potent drug, the human oral absorption (HOA) value should be >2 [40]. Further, as per the Lipinski's rule of five, the molecular weight should be <500, hydrogen bond accepting capability should be ≤10, and the donating ability should be ≤5. The ratio of octanol/water coefficient (QPlogPo/W) is expected to be <5. Considering all the descriptions and their scopes, the ability of a molecule to function as a drug can be assessed. The outcomes derived from the ADME assessment for all DAM derivatives are presented in Table 9. The findings suggest that all derivatives exhibited a drug‐like nature ranging from very good to moderate.

**TABLE 9:** Evaluated ADMET parameters for various diarylmethanes.

Entry	Stars	CNS	HOA	Mol.Wt	HBD	HBA	QPlogS	QPlogPW	QPlogPo/W	QPPCaCo	QPlogKp	QPlogBB
**7a**	2	1	1	400.95	0	2.5	−8.888	5.446	6.79	3749.262	−0.854	0.05
**7b**	1	0	1	396.531	0	3.25	−8.273	5.922	6.332	3754.524	−0.782	−0.189
**7c**	4	0	1	473.616	0	4.25	−9.848	8.528	7.465	3354.263	−0.194	−0.285
**7d**	3	0	1	416.565	0	2.5	−9.499	6.436	7.28	3456.12	−0.471	−0.182
**7e**	3	0	1	445.401	0	2.5	−9.096	5.583	6.864	3317.789	−0.919	−0.002
**9a**	4	0	1	428.576	0	2.5	−8.893	6.161	7.406	3405.481	−0.189	−0.282
**9b**	3	0	1	442.602	0	2.5	−9.734	5.922	7.836	3872.833	−0.239	−0.248
**9c**	4	0	1	478.635	0	2.5	−10.423	6.924	8.521	4447.314	0.374	−0.192
**9d**	5	0	1	519.688	0	3.5	−10.666	8.561	8.81	4016.128	0.559	−0.244
**9e**	4	0	1	478.635	0	2.5	−10.108	6.778	8.395	3736.536	0.144	−0.261
**Range**	**0–5**	**−2 to +2**	**1 – 3**	** <500**	**0–6**	**2 –20**	**−6.5 to 0.5**	**4–45**	**−2 to −6.5**	** <25 >500**	**−8 to −1**	

Abbreviations: CNS, central nervous system activity predicted on a scale from −2 (inactive) to +2 (active); CIQPlogS, conformation‐independent predicted aqueous solubility; HBA, hydrogen bond acceptor; HBD, hydrogen bond donor; HOA, human oral absorption; QPlogBB, estimated brain/blood partition coefficient; QPlogKp, skin permeability prediction, log Kp; QPlogPo/w, estimated octanol/water partition coefficient; QPlogPw, estimated water/gas partition coefficient; QPlogS, predicted aqueous solubility (conformation dependent); QPPCaco, apparent Caco‐2 cell permeability predicted in nm/s; Starts, violations.

## 6 Pharmacophore Modeling for Various Biaryl/Teraryl‐Cored Diarylmethane Derivatives

By employing the phase module of Schrödinger suite 2023–4, the pharmacophore hypothesis was developed for the variously designed **7a–e** and **9a–e** inhibitors [40, 41, 42]. A dataset was generated by assigning a threshold maximum and minimum binding free energies as predicted from the computational docking analysis (Table 7). To facilitate noncovalent interaction of the inhibitors with the receptor site binding residues, the pharmacophoric sites with the features including hydrophobic group (H), hydrogen bond acceptor (A), hydrogen bond donor (D), negative ionizable (N), positive ionizable (P), and aromatic ring (R) defined in module phase were constrained within a three‐dimensional space. Out of the total 10 ligands, four were selected as training sets, while the remaining were considered as test samples further to permit statistical comparison among the projected models. The assessment of thus obtained pharmacophore hypotheses was carried out by their scoring function to yield the highest possible alignment of ligands with high affinity while also incorporating features from compounds with low affinity, thereby enhancing the model's versatility [43]. In this case, the pharmacophore hypothesis was generated using the two active compounds, with 2 Å as the minimum intersite distance and maintaining the pharmacophore‐matching tolerance between two pharmacophoric features as 1 Å.

**FIGURE 19:**
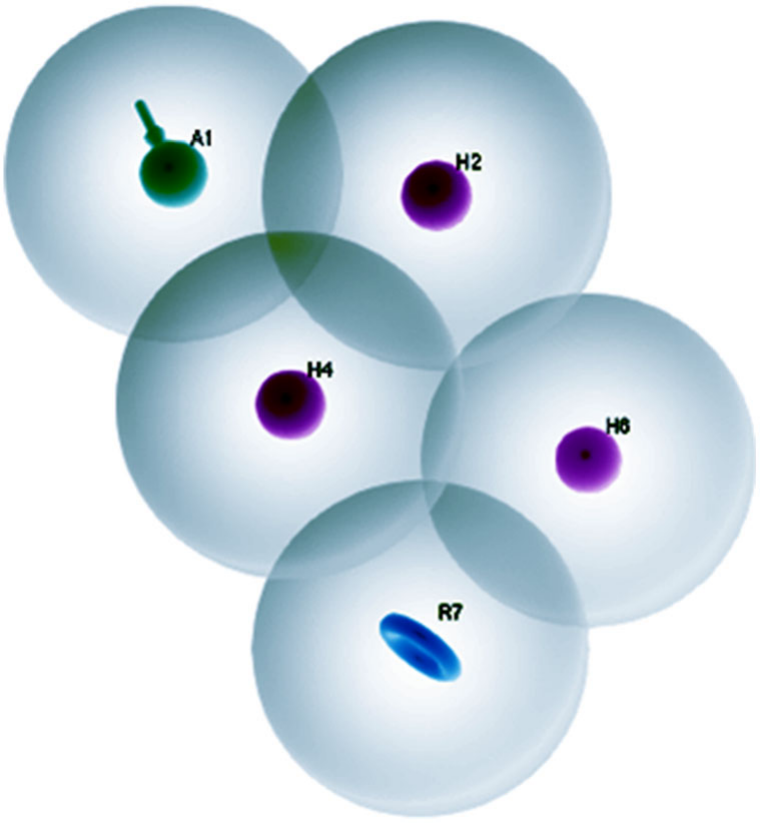
Pharmacophoric features obtained from the generated models.

**FIGURE 20:**
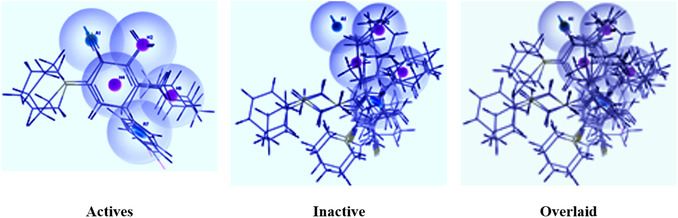
Pharmacophore model depicting the features predicted for various DAM ligands in the dataset.

A total of 20 variant hypotheses were generated by assigning two as the maximum number of sites and 50% as active. The pertinent data are given in Table 10. Among the above‐generated hypotheses, the best one was selected based on their survival score, survival inactive, site score, volume, vector, energy terms, and the number of matches (Table 11). Various pharmacophore hypotheses and the pharmacophoric scores obtained from the best hypothesis are depicted in Figures 19 and 20, respectively.

**TABLE 10:** Generated pharmacophore hypotheses for various DAM ligands based on their affinity toward the active site of PARP1 enzyme.

HypoID	Survival	Site	Vector	Volume	Select	Matches	Inactive	Adjusted
AHHHR_1	4.7484	0.7529	0.9861	0.7899	1.9184	2	1.2122	3.5362
AHHHR_2	4.7394	0.7897	0.9669	0.7788	1.9031	2	1.1549	3.5845
AHHHR_3	4.7340	0.7928	0.9593	0.783	1.8978	2	1.4131	3.3209
AHHHR_4	4.6856	0.7829	0.9818	0.716	1.904	2	1.4669	3.2188
AHHHR_5	4.6835	0.7077	0.9898	0.7058	1.9791	2	1.7313	2.9522
AHHHR_6	4.6667	0.705	0.9906	0.7136	1.9564	2	1.5311	3.1357
AHHHR_7	4.6461	0.7693	0.986	0.6968	1.893	2	1.1435	3.5026
AHHHR_8	4.6342	0.7228	0.9893	0.7157	1.9054	2	1.2622	3.372
AHHHR_9	4.6272	0.6537	0.9829	0.7841	1.9055	2	0.753	3.8742
AHHHR_10	4.6248	0.6519	0.9707	0.791	1.9101	2	1.0836	3.5412
HHHR_1	4.3654	0.8332	0.9788	0.7909	1.4615	2	1.1772	3.1882
HHHR_2	4.3522	0.8335	0.9772	0.792	1.4485	2	1.2565	3.0957
HHHR_3	4.3520	0.9263	0.9989	0.6638	1.462	2	0.9858	3.3662
HHHR_4	4.3175	0.7953	0.9846	0.7545	1.4821	2	0.8258	3.4917
HHHR_5	4.1561	0.756	0.8129	0.7046	1.4054	3	1.5357	2.6204
HHHR_6	4.0406	0.6239	0.9845	0.5197	1.4353	3	1.5287	2.5119
HHHR_7	4.0281	0.5595	0.9227	0.6157	1.4531	3	1.9279	2.1003
HHHR_8	4.0149	0.6385	0.8946	0.5394	1.4653	3	1.8577	2.1571
HHHR_9	4.0076	0.6653	0.942	0.4885	1.4346	3	1.5479	2.4596
AHHR_1	3.9379	0.5816	0.8979	0.5449	1.4364	3	1.2791	2.6588

**TABLE 11:** Different parametric scores of the generated hypothesis AHHHR_9.

AHHHR_9	Activity	Fitness	Site score	Vector score	Volume	Matched ligand site
1	Active	3	1	1	1	A(1) H(2) H(4) H(6) R(7)
2	Active	2.421	0.654	0.983	0.784	A(1) H(2) H(4) H(6) R(8)
7	Inactive	0.703	0.239	0.172	0.346	A(‐) H(2) H(3) H(5) R(7)
8	Inactive	0.904	0.511	0.186	0.344	A(‐) H(5) H(2) H(4) R(7)
9	Inactive	0.71	0.228	0.179	0.353	A(‐) H(3) H(2) H(5) R(8)
9	Inactive	0.695	0.228	0.179	0.339	A(‐) H(3) H(2) H(5) R(8)

## 7 Pan Assay Interference Compounds

Further to eliminate the false positives from the proposed library of compounds, an online tool PAINS was utilized [43]. This assay helps to exclude the compounds from further analysis. The results revealed that all the compounds **7a–e** and **9a–e** passed the PAINS filter and could be proposed as drug candidates.

## 8 Conclusions

Herein, a series biaryl/teraryl‐cored diarylmethanes were designed and investigated for their broad‐spectrum biological applications. The electronic properties of these derivatives in their ground and excited states were evaluated through experimental and computational approaches employing DFT methods. No significant changes were observed in the absorption spectra, while a significant redshift was observed in the fluorescence emission spectra when moving from nonpolar to polar aprotic/protic solvents, suggesting an increase in the dipolar interaction upon excitation. Moreover, a slight increase in the quantum yield was observed, indicating reduced nonradiative transitions. The interaction with biomacromolecules was ascertained by the appearance of an isoemissive point that further indicates the equilibrium between the bound and unbound states. Similar results were also observed with nucleic acid binding. Also, compounds **7d** (biaryl‐cored) and **9b** (teraryl‐cored) showed good potential toward inhibiting protein denaturation and hence possess excellent anti‐inflammatory character.

Compound **7d** has shown strong binding potential to the active site of PARP1. The same was supported by molecular docking and dynamic simulations. Based on the results of all the investigations, compound **7d** could be proposed as the most potent candidate with good druggable potential to inhibit PARP1, the well‐known cancer target.

## Funding

The authors have nothing to report.

## Conflicts of Interest

The authors declare no conflicts of interest.

## Supporting information

Supplementary Material

## Data Availability

The data that support the findings of this study are available in the Supporting Information of this article.
