# Supplementary material for: Evaluation of Photophysical Properties and Biological Applications of Diarylmethanes
Source: ChemistryOpen. 2026 May 25;15(6):e202500601. doi: 10.1002/open.202500601 (PMC13240489; doi:10.1002/open.202500601)
Supplement: Supplementary file 1 — Supplementary Material [file OPEN-15-e202500601-s001.pdf]

SUPPORTING INFORMATION

Evaluation of Photophysical Properties and Biological Applications of Diarylmethanes

Ranjini Jenifer H, Fateh V. Singh* and Balamurali MM*

[a] Department of Chemistry, School of Advanced Sciences, Vellore Institute of Technology, Chennai Campus, Vandalur Kelambakkam Road, Chennai 600 127.

[b] Centre for Healthcare Advancement, Innovation and Research, Vellore Institute of Technology, Chennai Campus, Vandalur Kelambakkam Road, Chennai 600 127.

Email: balamurali.mm@vit.ac.in

TABLE OF CONTENT

^{1}H and ^{13}C NMR spectral data of Diarylmethanes	**2-18**
HRMS data	**19-27**
**Table S1.** Computationally evaluated electronic structure properties in the ground and excited state of various diarylmethanes	**28-29**
**Figure S10.** Representation of charge density distribution in the highest occupied (HOMO) and lowest unoccupied (LUMO) molecular orbitals of various DAMs (**7a-e** and **9a-e**) along with their energy gap in eV.	**30-32**
**Figure S11.** Schematic representing the docked interactions of various diarylmethane derivatives **7a-e** and **9a-e** with PARP1 (a) 3D representation of protein – ligand complex in the binding pocket (b) 2-dimensional representation of the binding of interacting residues and various derivatives (c) 2-dimensional representation showing the type of interactions involved in the binding of PARP1 with various derivatives.	**33-35**
**Figure S12.** % Contribution of various stabilizing interactions between **7a-e** and **9a-e** and PARP1 binding pocket residues	**36**

^{1}H NMR (400 MHz, $CDCl_3$): 2-Benzyl-4′-chloro-3-methyl-5-(piperidin-1-yl)[1,1′-biphenyl]-4 carbonitrile **7a**:

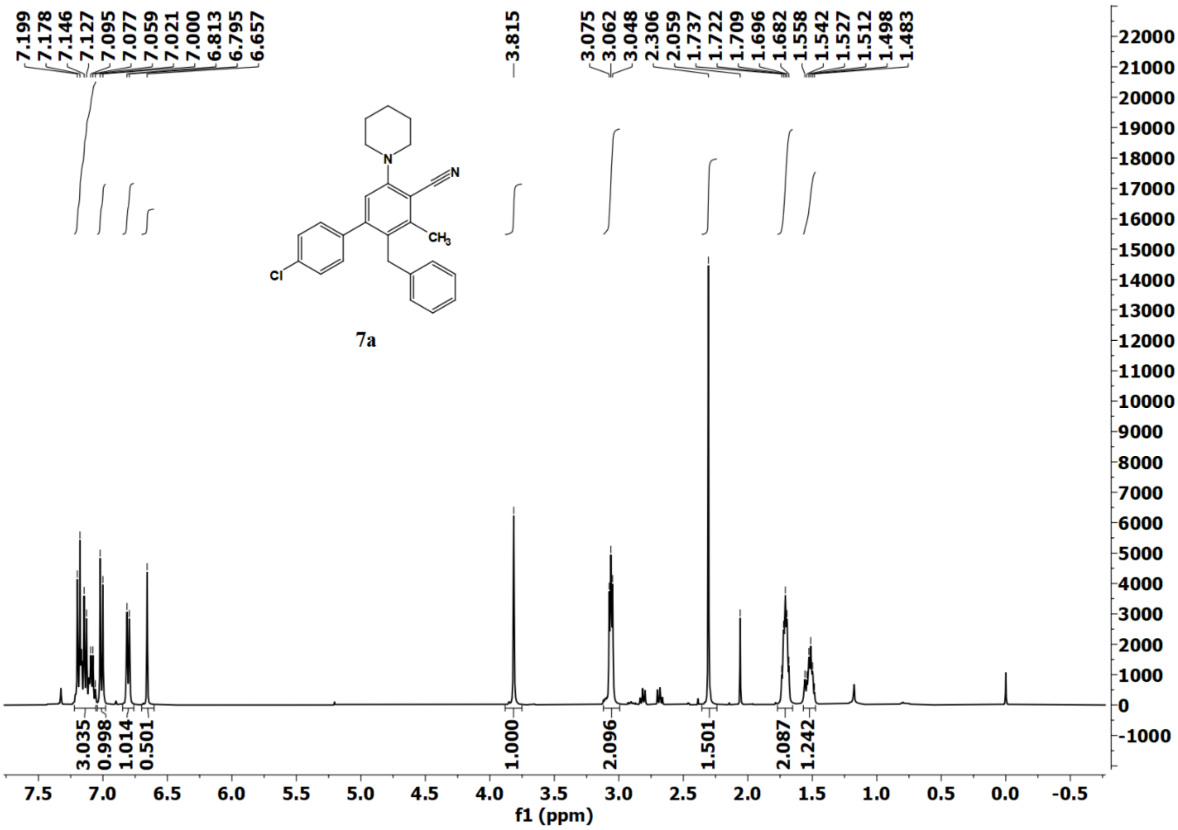

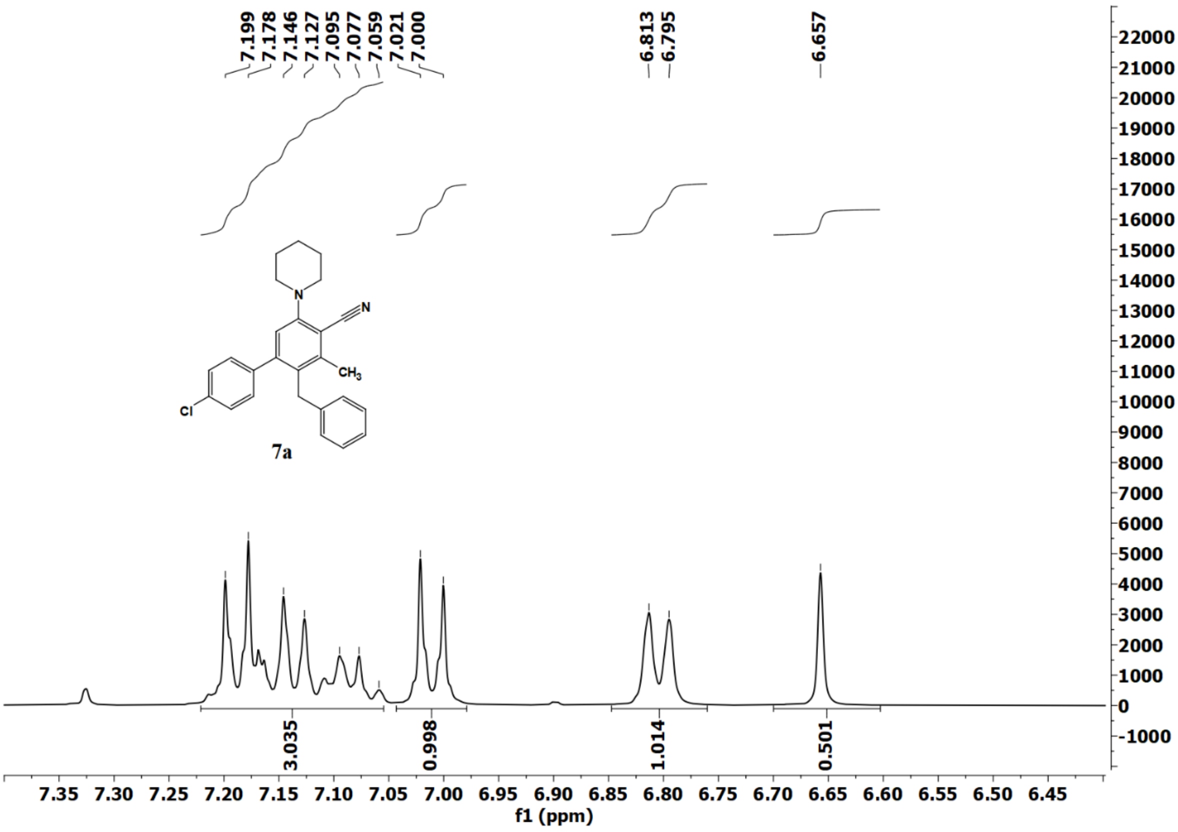

^{13}C NMR (400 MHz, CDCl3): 2-Benzyl-4′-chloro-3-methyl-5-(piperidin-1-yl)[1,1′-biphenyl]-4 carbonitrile **7a**:

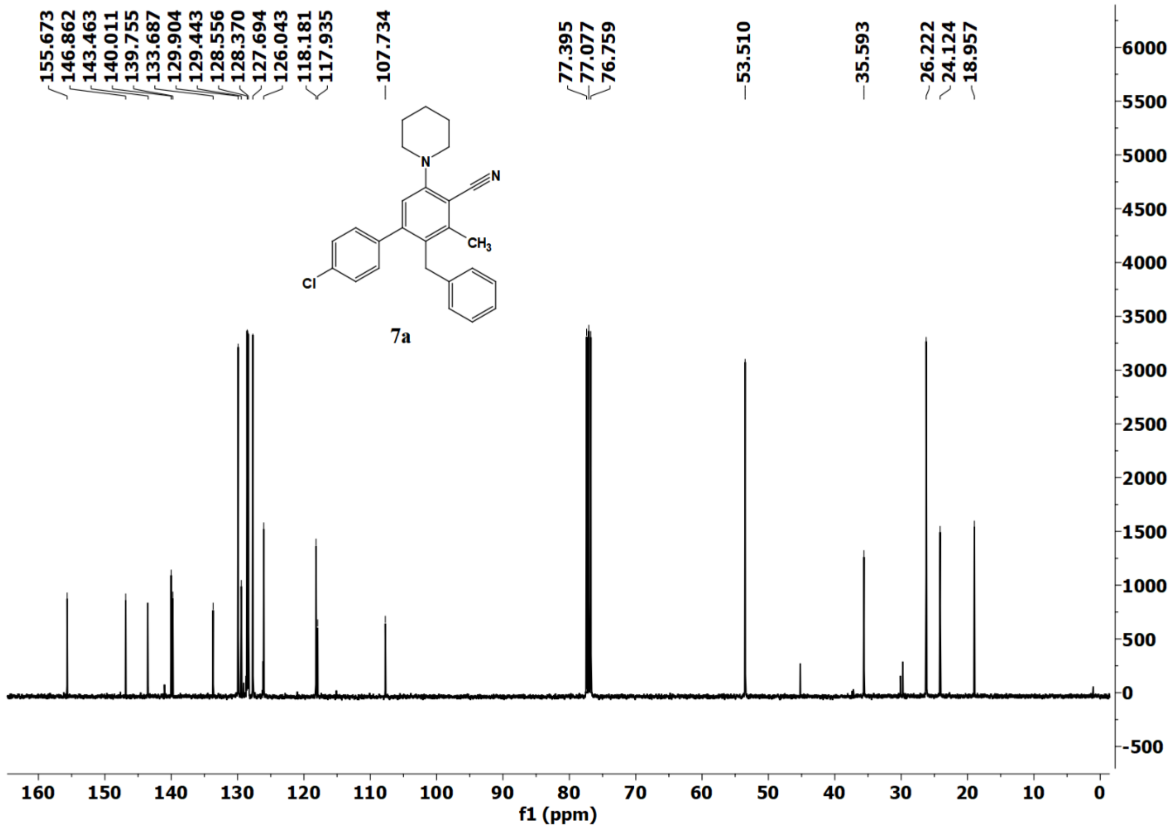

^{1}H NMR (400 MHz, CDCl3): 2-Benzyl-4′-methoxy-3-methyl-5-(piperidin-1-yl)[1,1′-biphenyl] 4-carbonitrile **7b**:

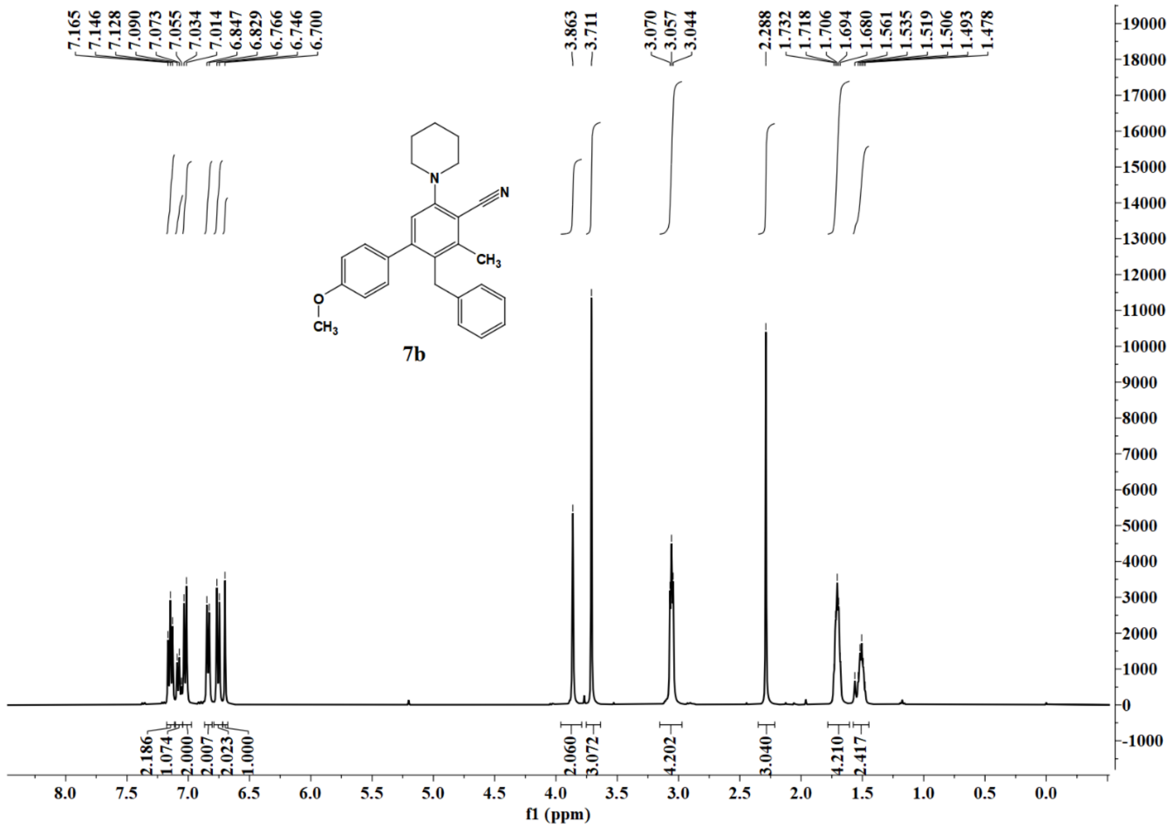

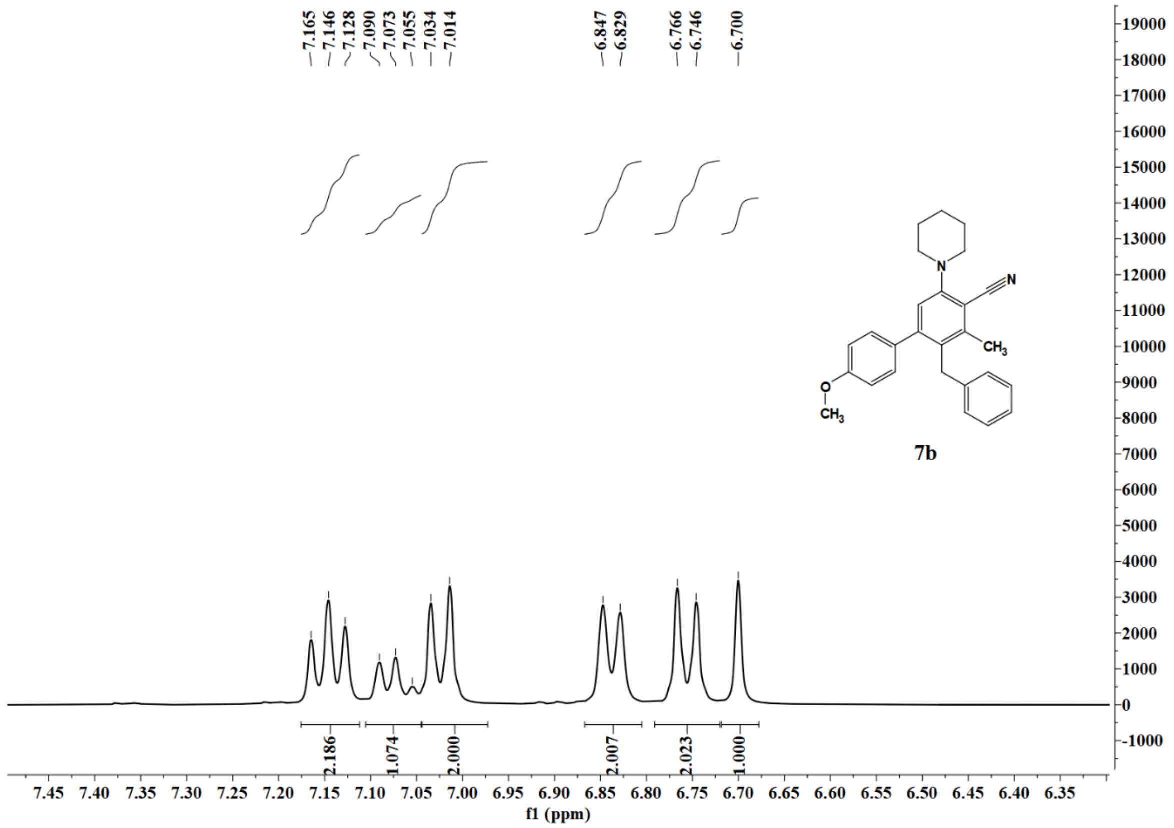

^{13}C NMR (400 MHz, $CDCl_3$): 2-Benzyl-4′-methoxy-3-methyl-5-(piperidin-1-yl)[1,1′-biphenyl] 4-carbonitrile **7b**:

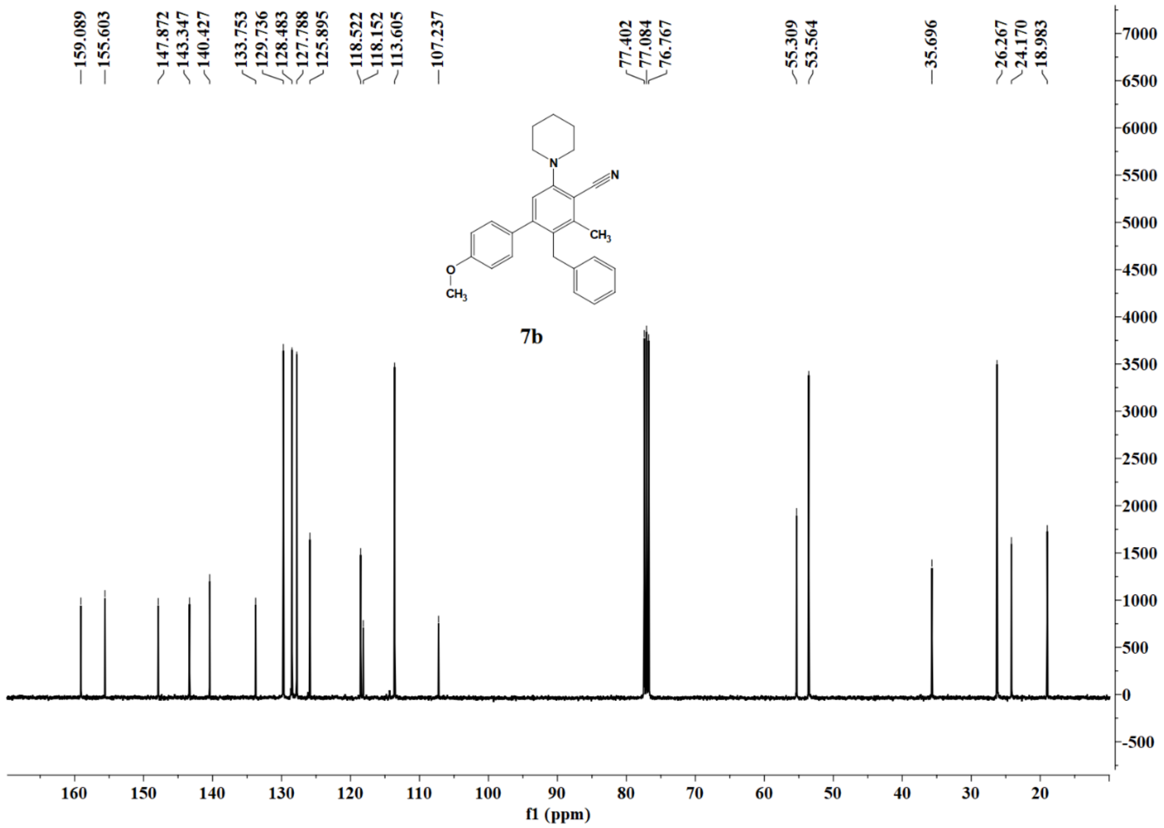

^{1}H NMR (400 MHz, $CDCl_3$): 2-Benzyl-4′-methoxy-3-methyl-5-(4-phenylpiperazin-1-yl)[1,1′biphenyl]-4-carbonitrile **7c**:

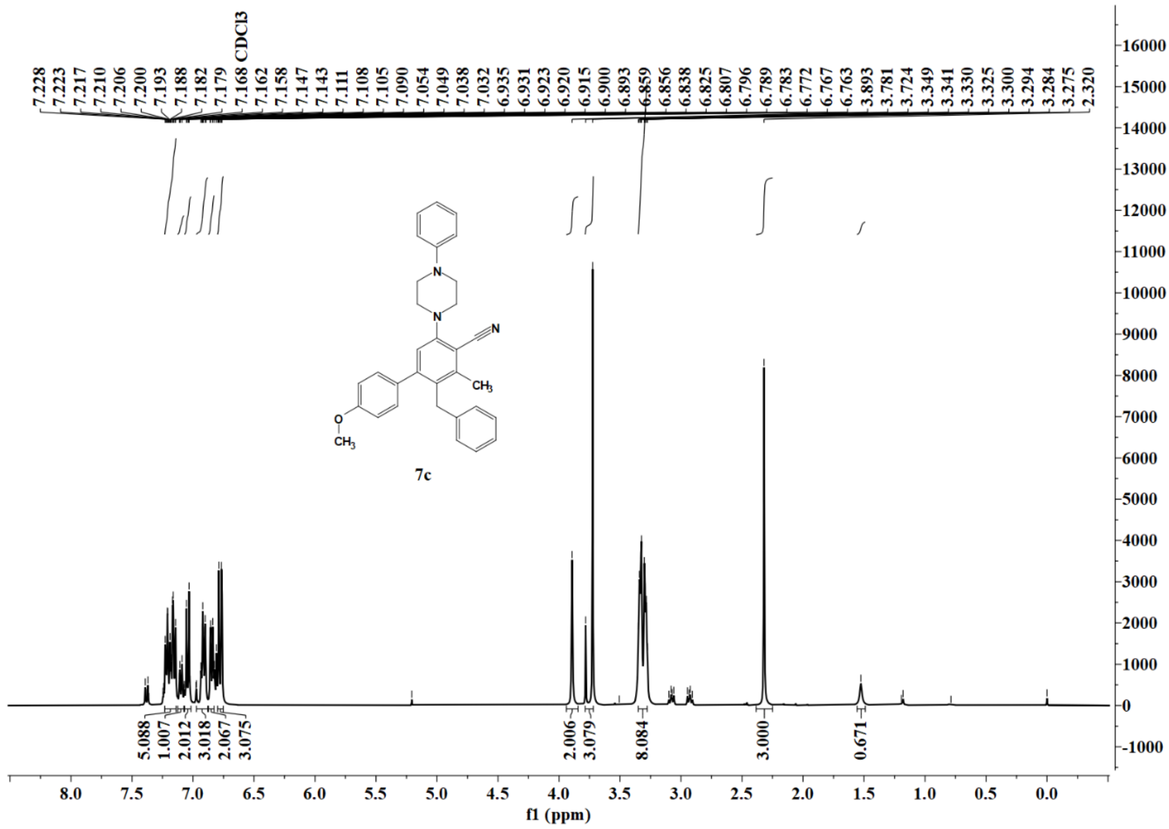
7.228 7.223 7.217 7.210 7.206 7.200 7.193 7.188 7.182 7.179 7.168 CDCl3 7.162 7.158 7.147 7.143 7.111 7.108 7.105 7.090 7.054 7.049 7.038 7.032 6.935 6.931 6.923 6.920 6.915 6.900 6.893 6.859 6.856 6.838 6.825 6.807 6.796 6.789 6.783 6.772 6.767 6.763 3.893 3.781 3.724 3.349 3.341 3.330 3.325 3.300 3.294 3.284 3.275 2.320
N
N
N
CH3
O
CH3
7c
5.088 1.007 2.012 3.018 2.067 3.075 2.006 3.079 8.084 3.000 0.671
f1 (ppm)

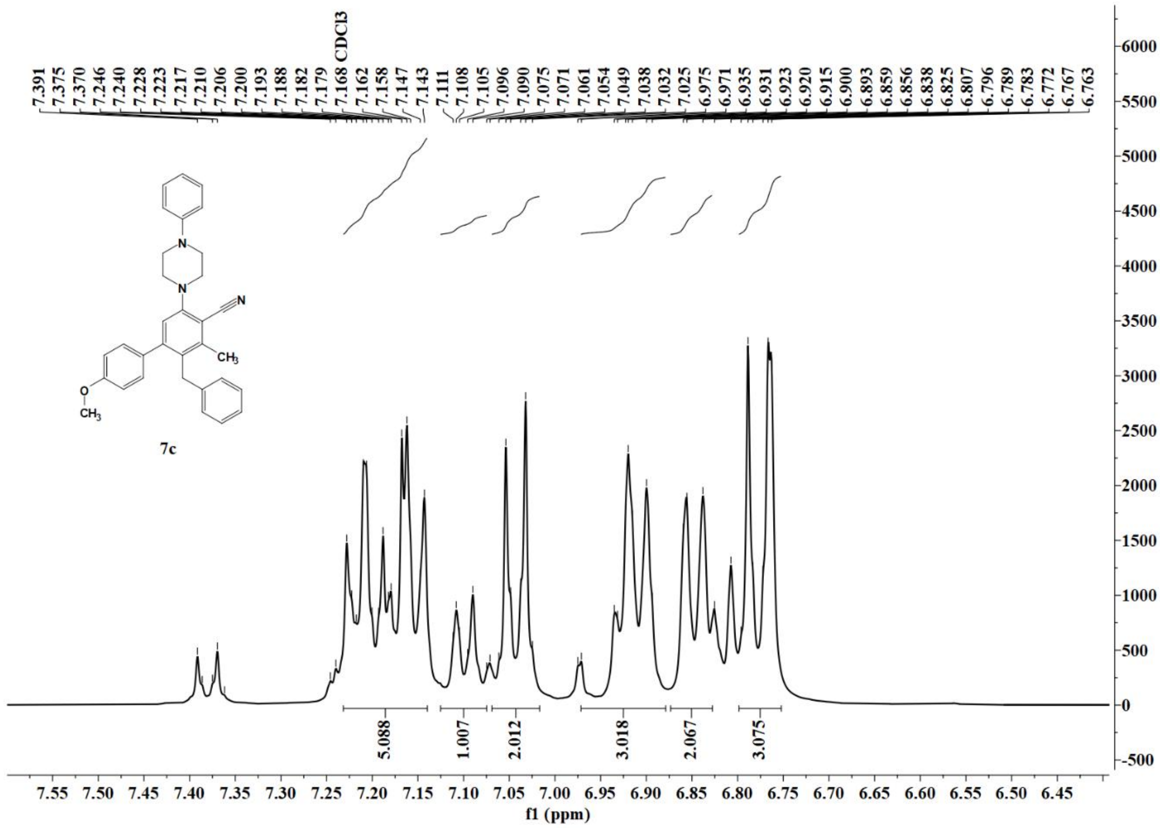
7.391 7.375 7.370 7.246 7.240 7.228 7.223 7.217 7.210 7.206 7.200 7.193 7.188 7.182 7.179 7.168 CDCl3 7.162 7.158 7.147 7.143 7.111 7.108 7.105 7.096 7.090 7.075 7.071 7.061 7.054 7.049 7.038 7.032 7.025 6.975 6.971 6.935 6.931 6.923 6.920 6.915 6.900 6.893 6.859 6.856 6.838 6.825 6.807 6.796 6.789 6.783 6.772 6.767 6.763
N
N
N
CH3
O
CH3
7c
5.088 1.007 2.012 3.018 2.067 3.075
f1 (ppm)

^{13}C NMR (400 MHz, $CDCl_3$): 2-Benzyl-4′-methoxy-3-methyl-5-(4-phenylpiperazin-1-yl)[1,1′biphenyl]-4-carbonitril*e* **7c**:

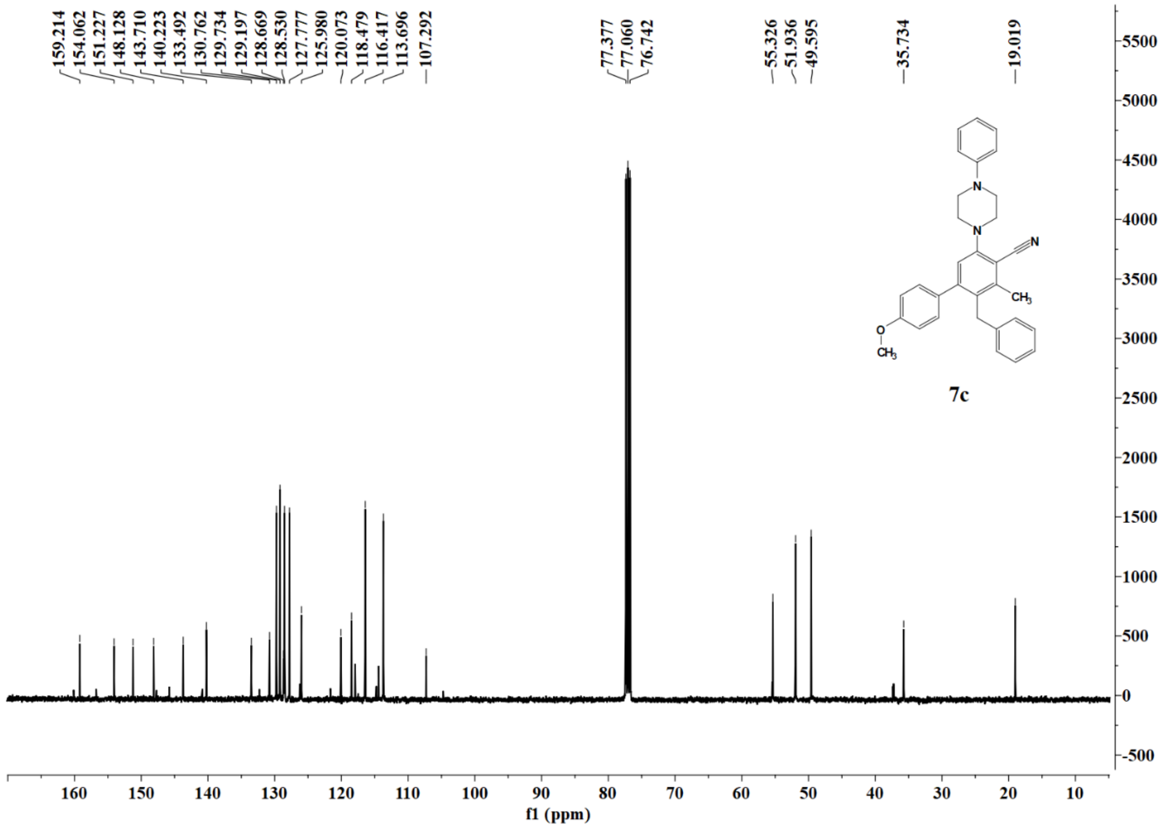

^{1}H NMR (400 MHz, $CDCl_3$): 3-Benzyl-2-methyl-4-(naphthalen-2-yl)-6-(piperidin-1-yl)benzo nitrile **7d**:

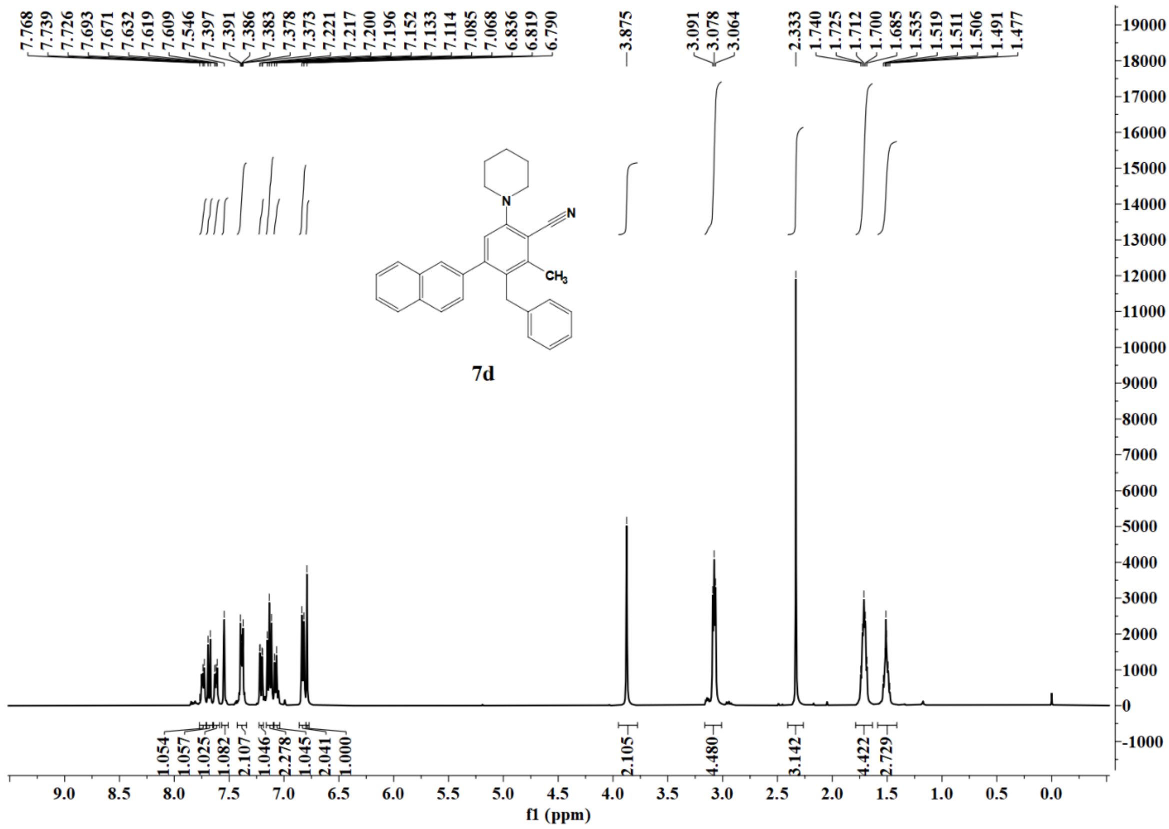

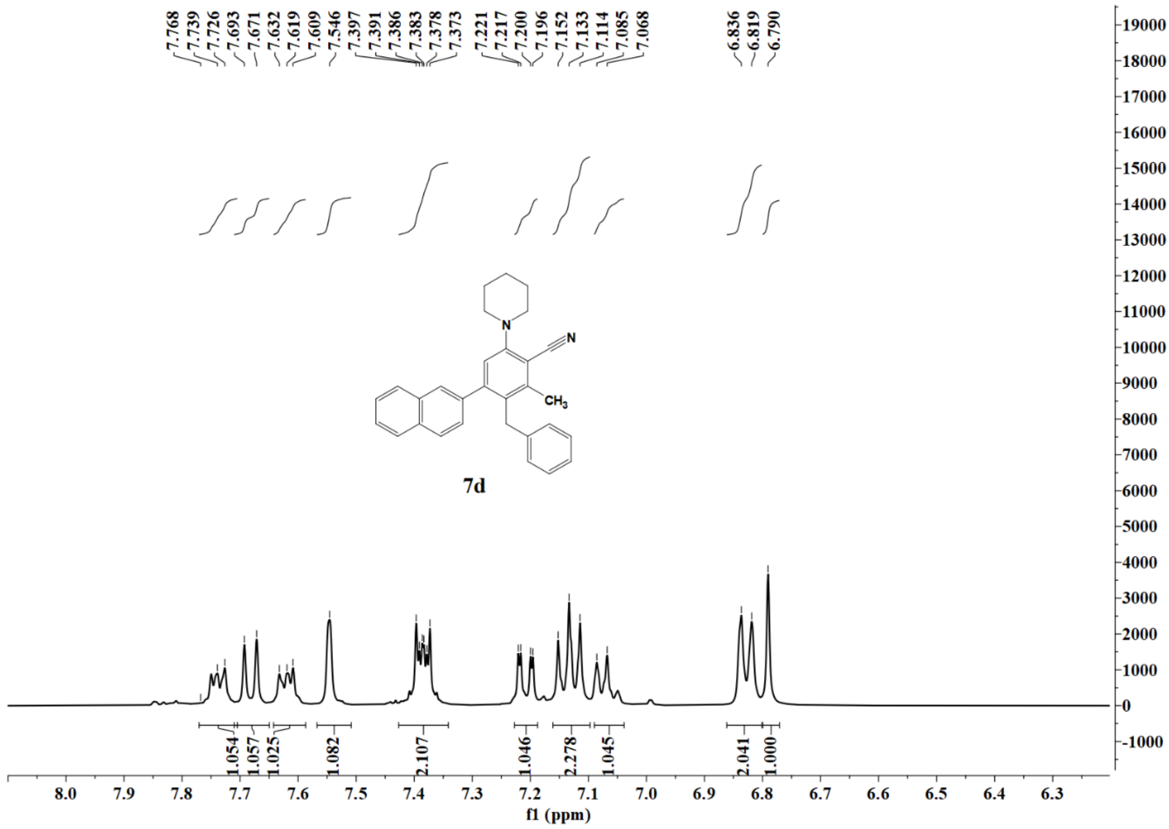

^{13}C NMR (400 MHz, $CDCl_3$): 3-Benzyl-2-methyl-4-(naphthalen-2-yl)-6-(piperidin-1-yl)benzonitrile **7d**:

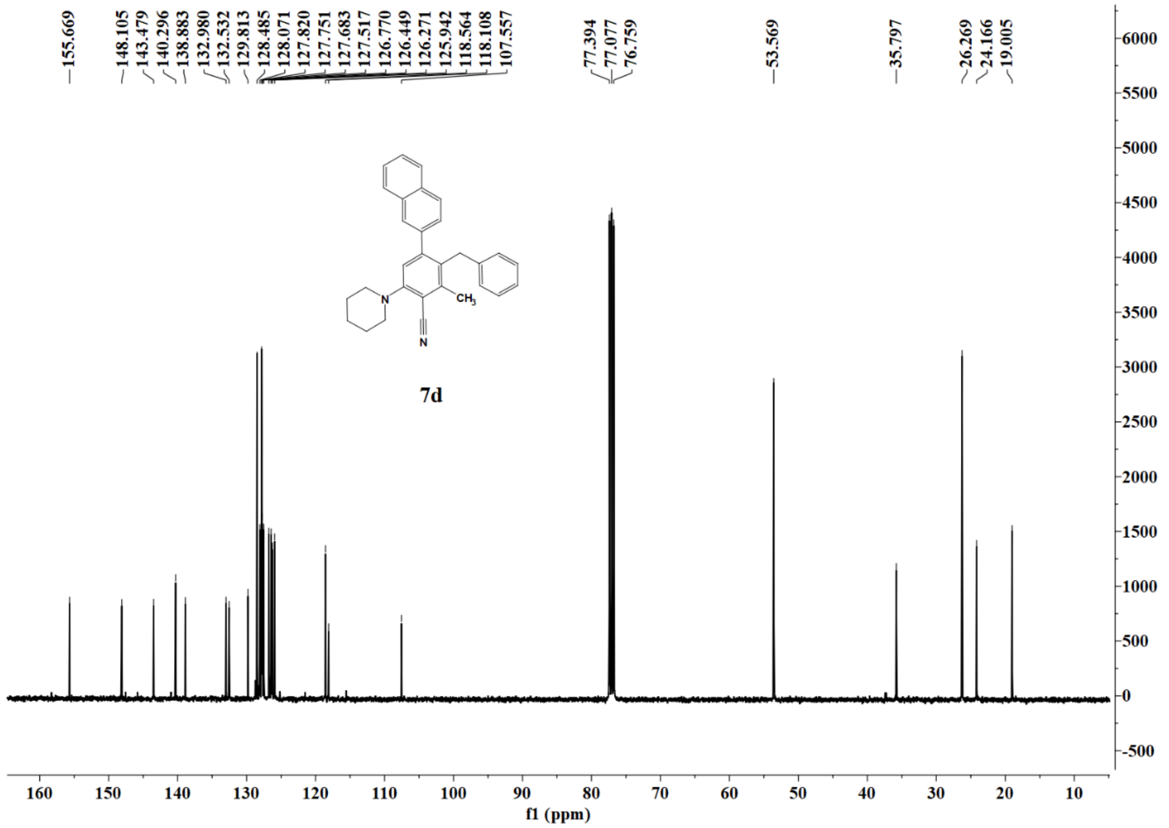

^{1}H NMR (400 MHz, $CDCl_3$): 2-benzyl-4’-bromo-3-methyl-5-(piperidin-1-yl)-[1,1’-biphenyl]-4-carbonitrile **7e**:

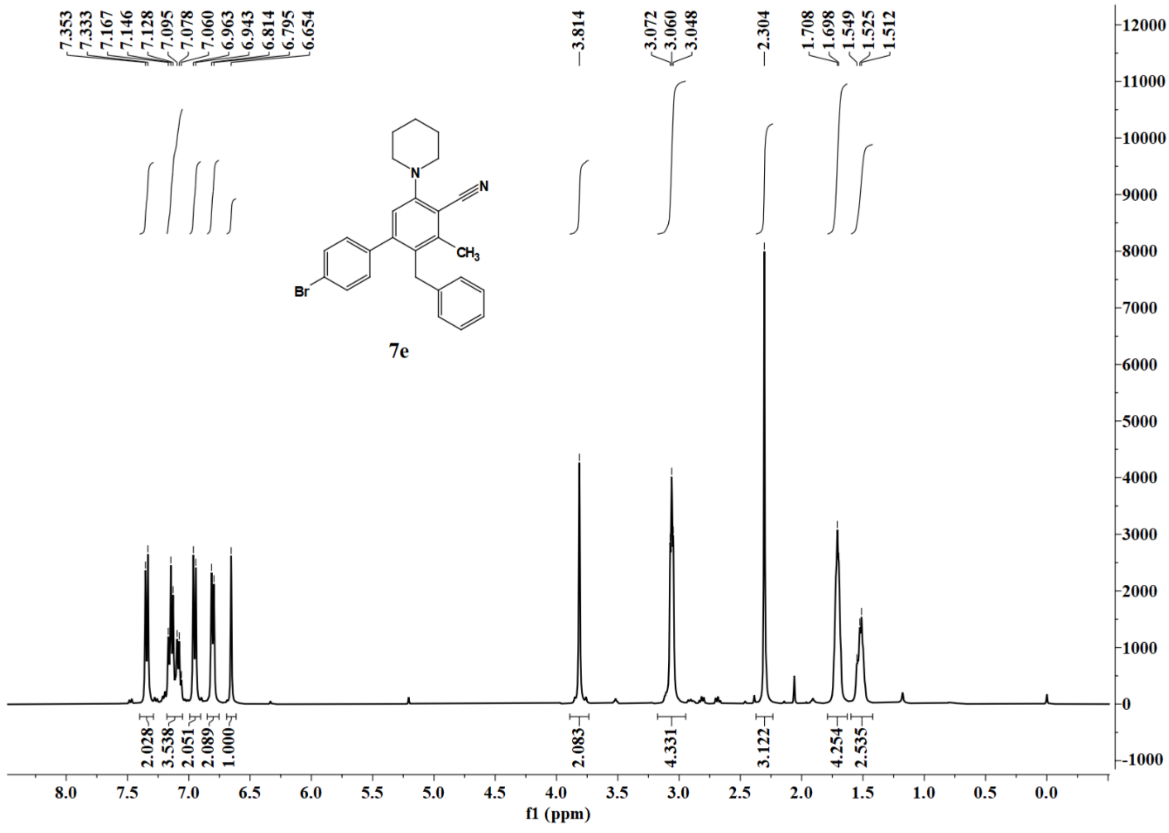

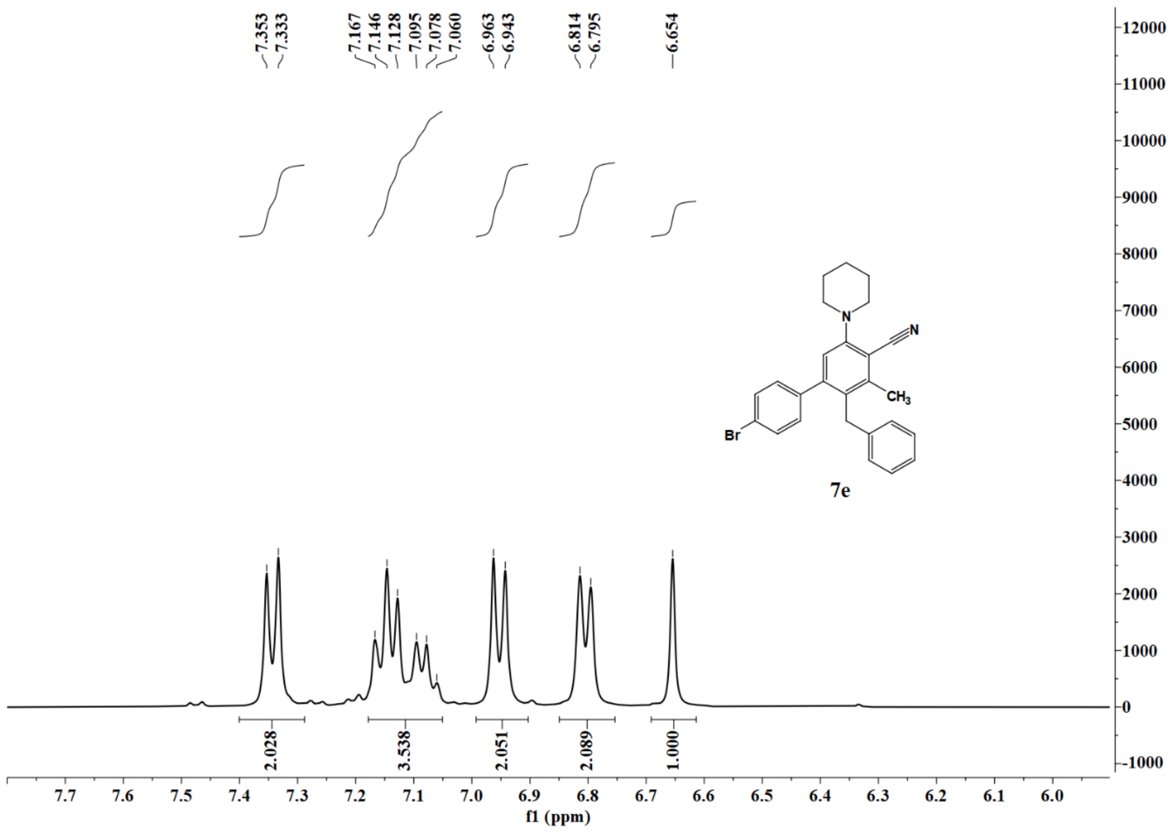

^{13}C NMR (400 MHz, $CDCl_3$): 2-benzyl-4'-bromo-3-methyl-5-(piperidin-1-yl)-[1,1'-biphenyl]-4-carbonitrile **7e**:

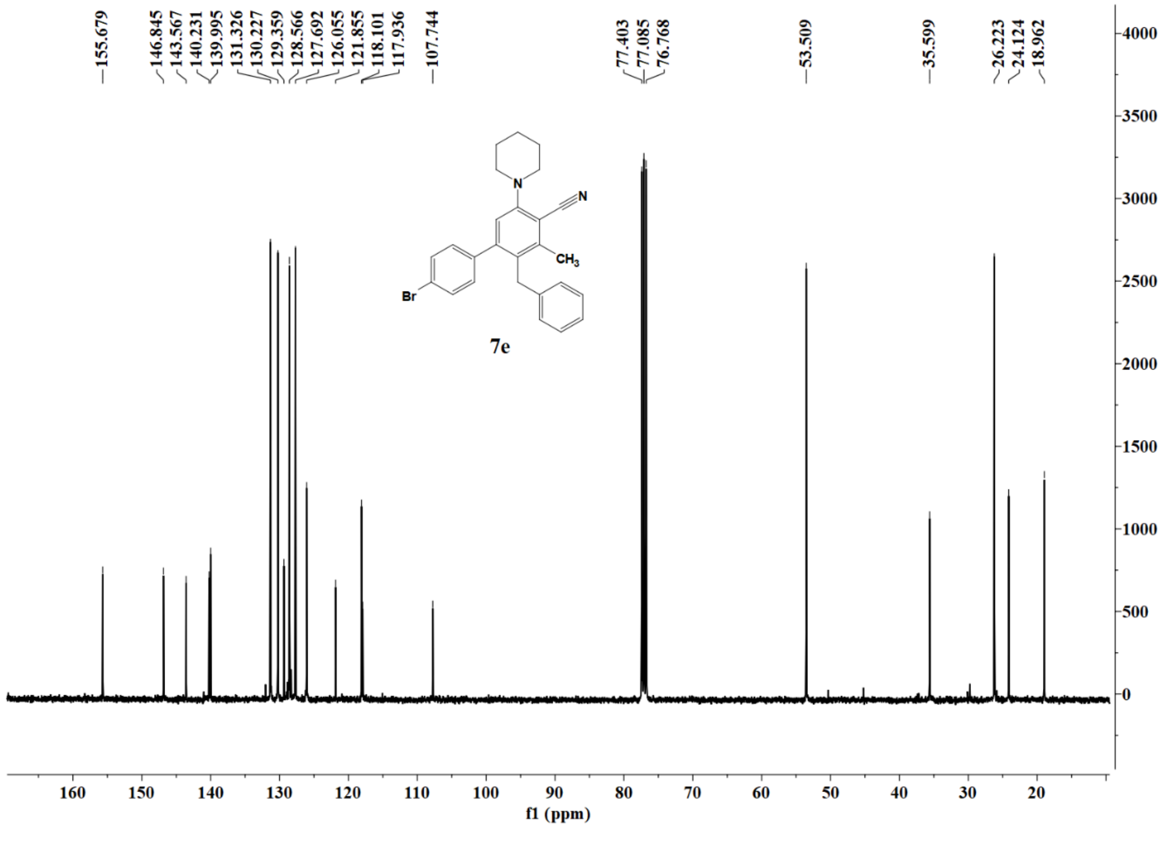

^{1}H NMR (400 MHz, $CDCl_3$): 3’-benzyl-5’-(piperidin-1-yl)-[1,1’,2’,1”-terphenyl]-4’-carbonitrile **9a**:

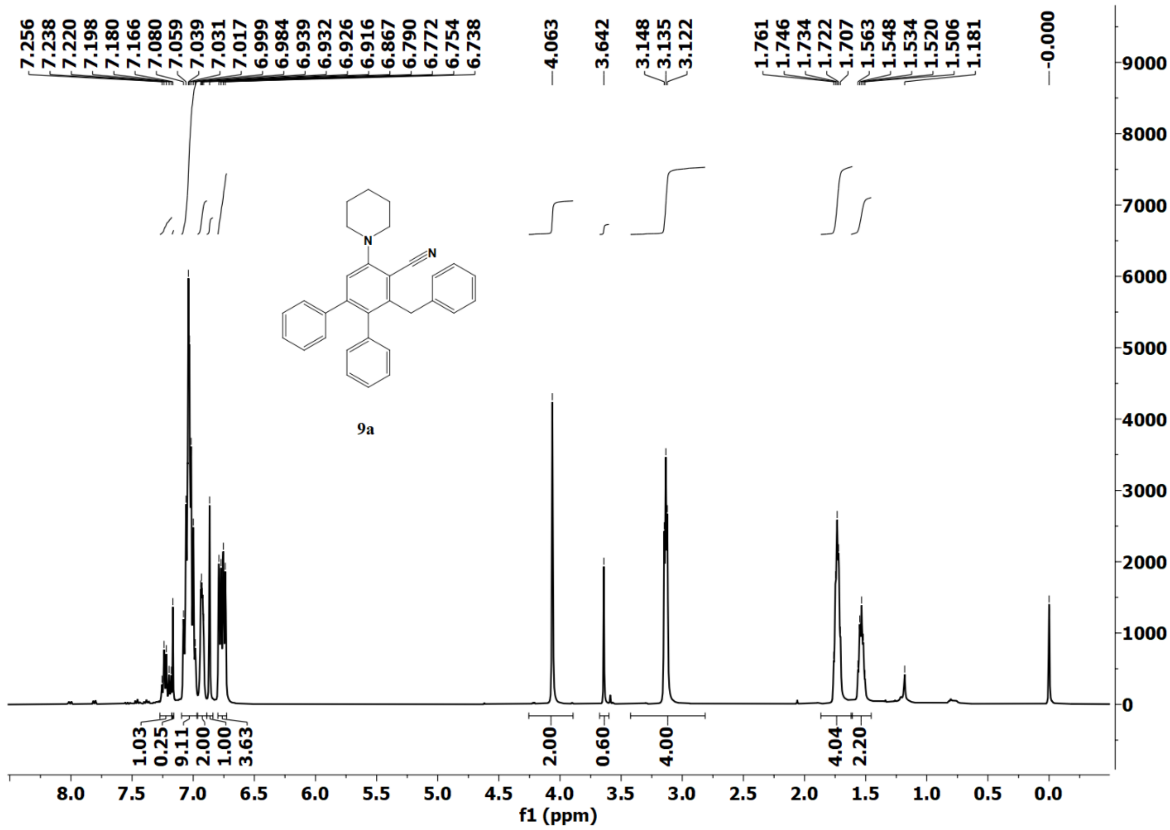

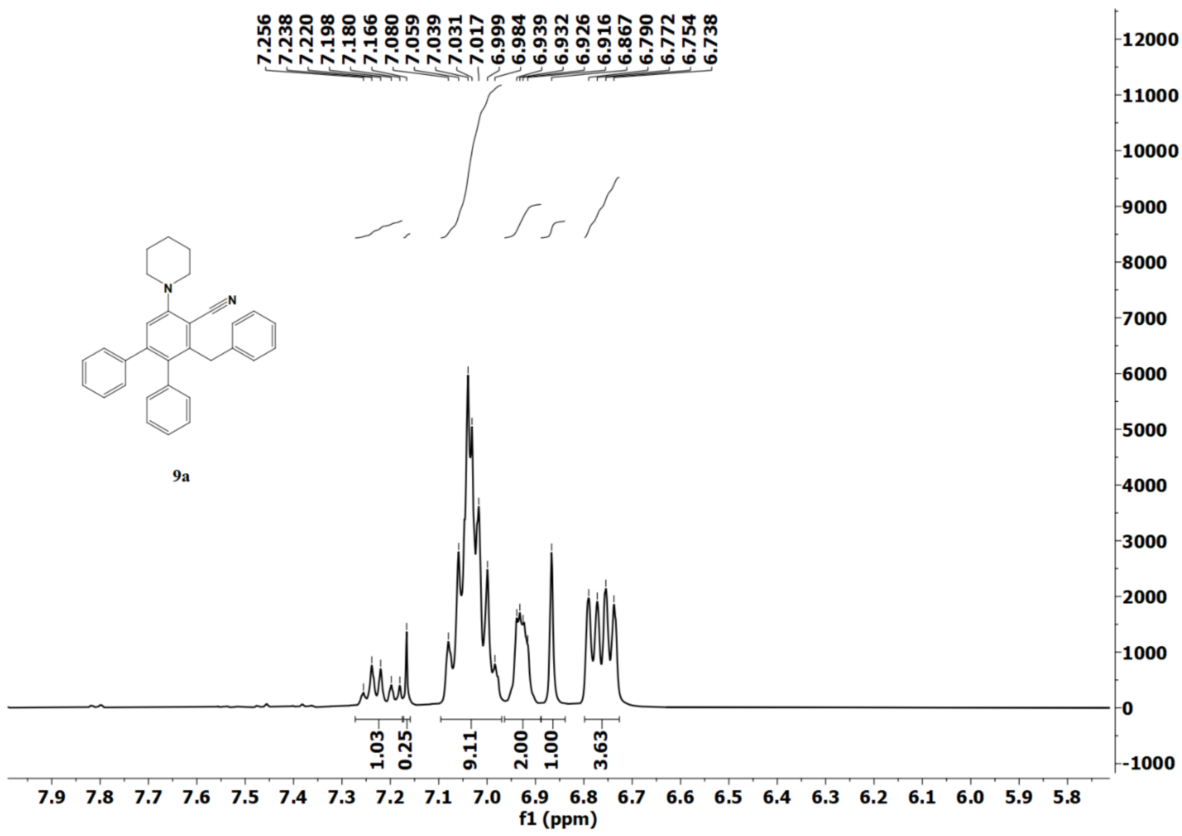

^{13}C NMR (400 MHz, $CDCl_3$): 3’-benzyl-5’-(piperidin-1-yl)-[1,1’,2’,1”-terphenyl]-4’-carbonitrile **9a**:

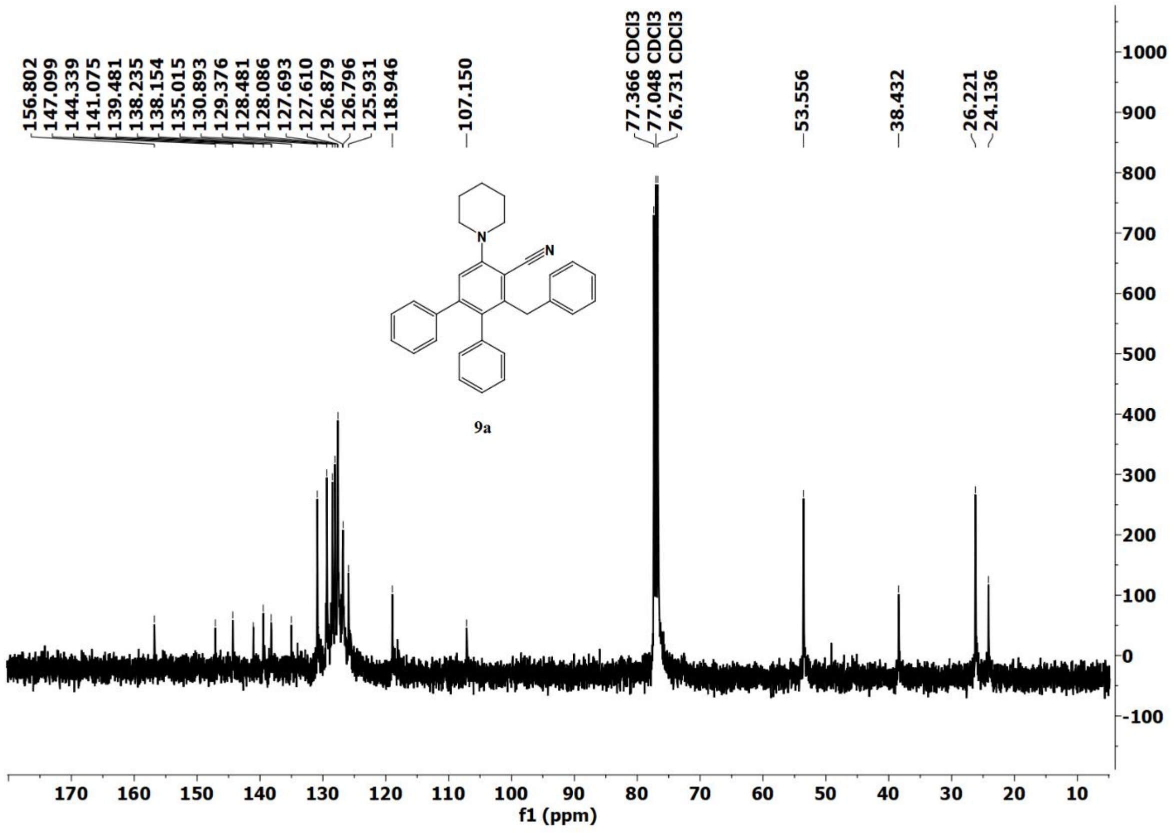

^{1}H NMR (400 MHz, $CDCl_3$): 3’-benzyl-4-methyl-5’-(piperidin-1-yl)-[1,1’,2’,1”-terphenyl]-4’-carbonitrile **9b**:

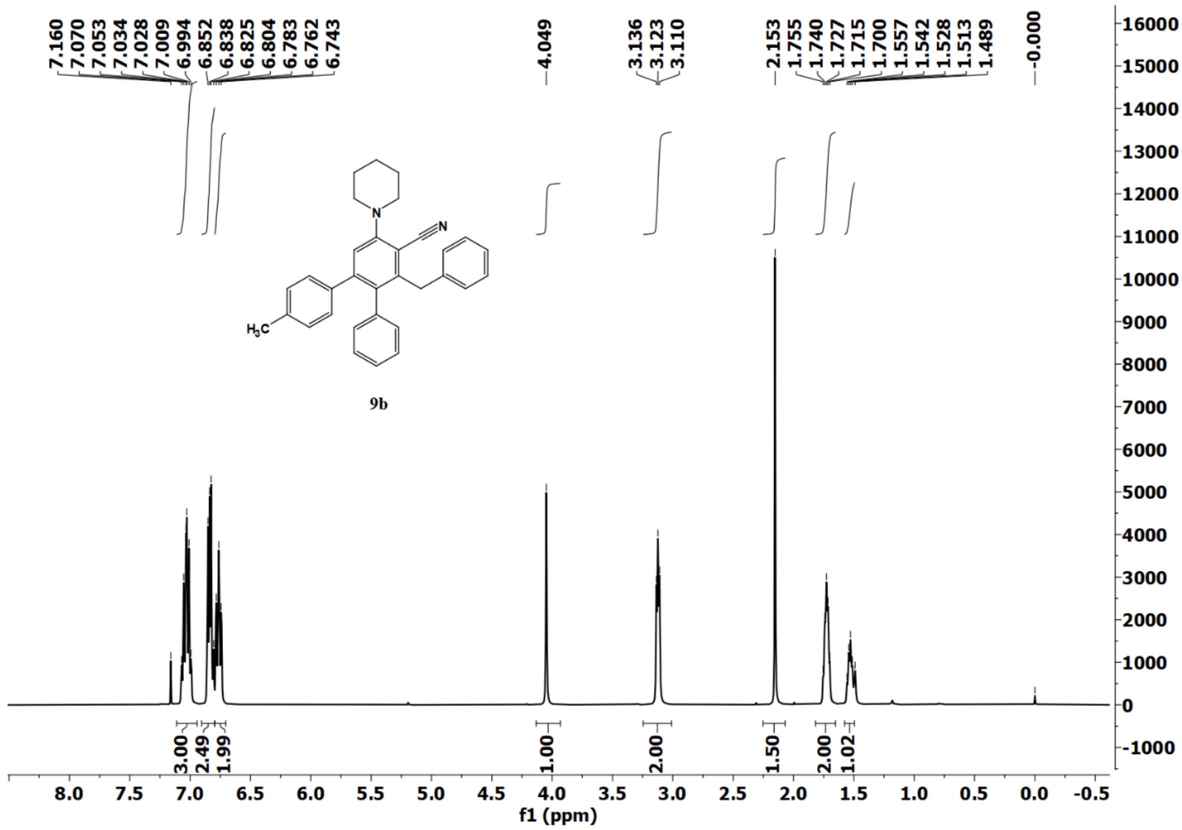

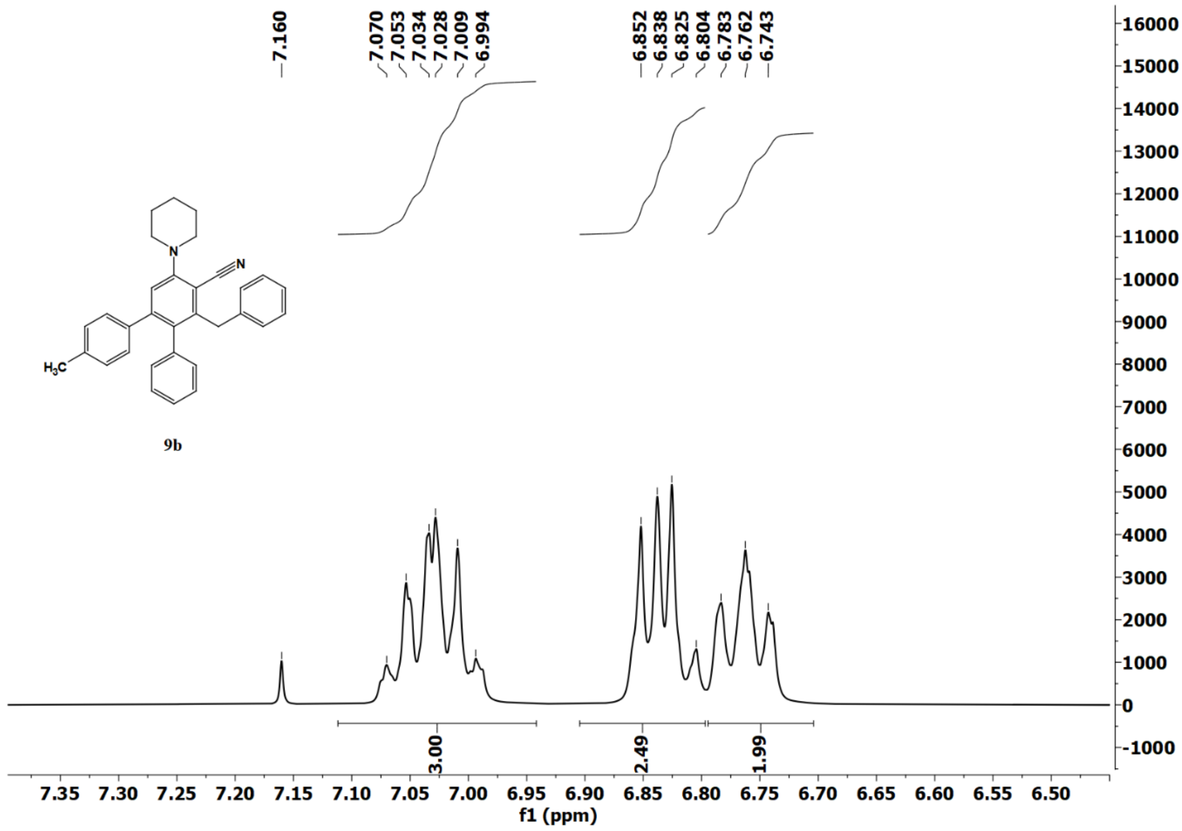

^{13}C NMR (400 MHz, $CDCl_3$): 3’-benzyl-4-methyl-5’-(piperidin-1-yl)-[1,1’,2’,1’’-terphenyl]-4’-carbonitrile 7g **9b**:

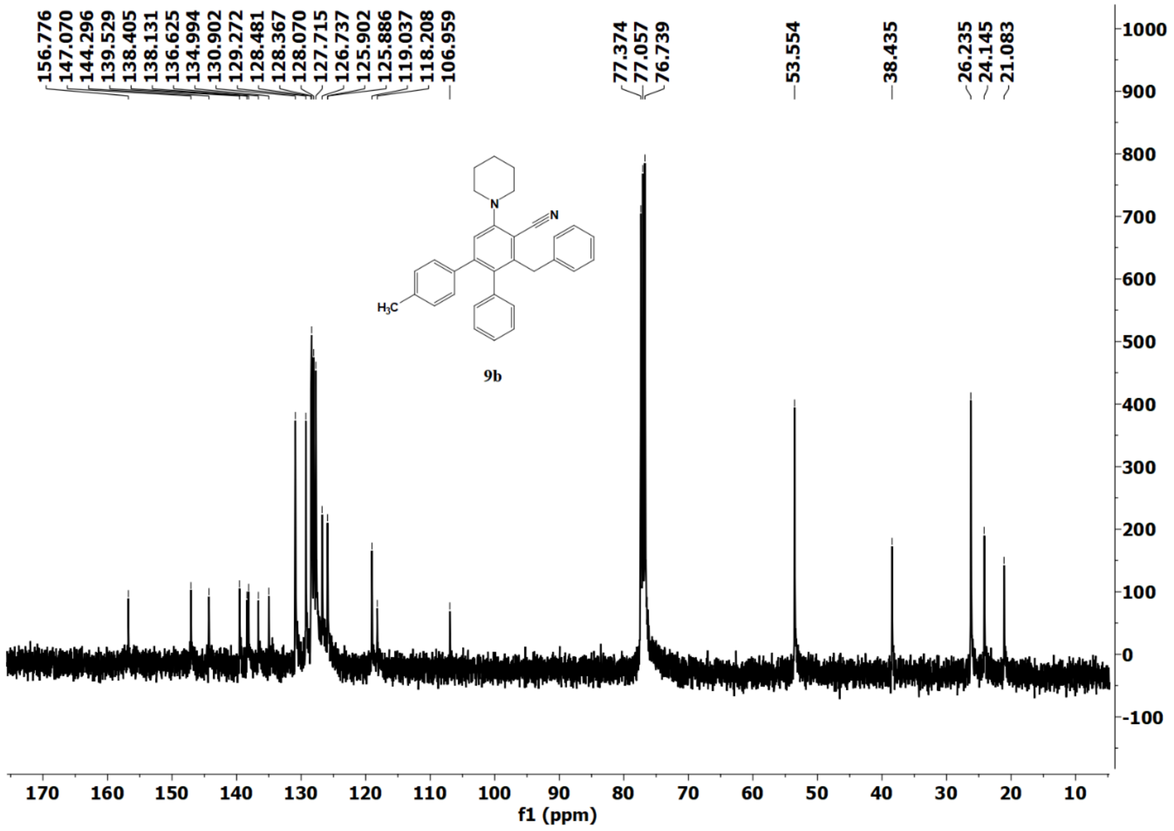

^{1}H NMR (400 MHz, $CDCl_3$): 2-benzyl-6-(naphthalen-2-yl)-4-(piperidin-1-yl)-[1,1'-biphenyl]-3-carbonitrile **9c**:

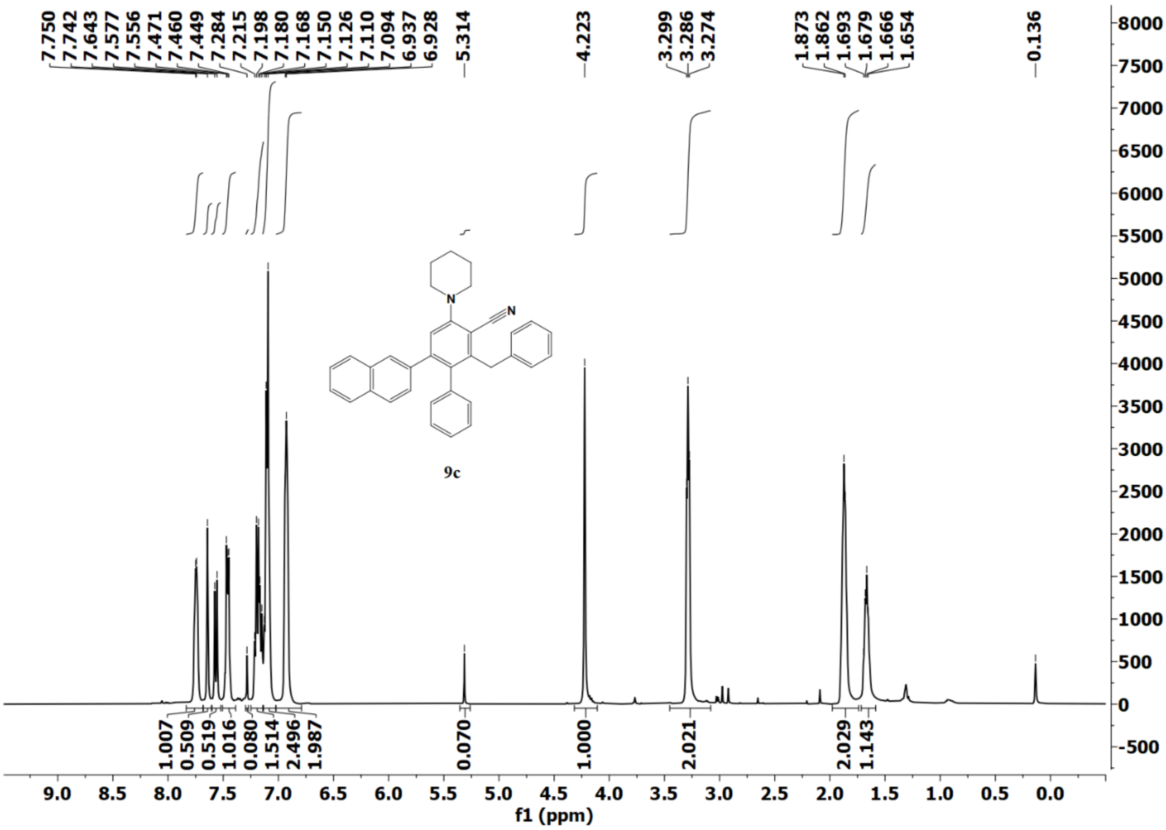

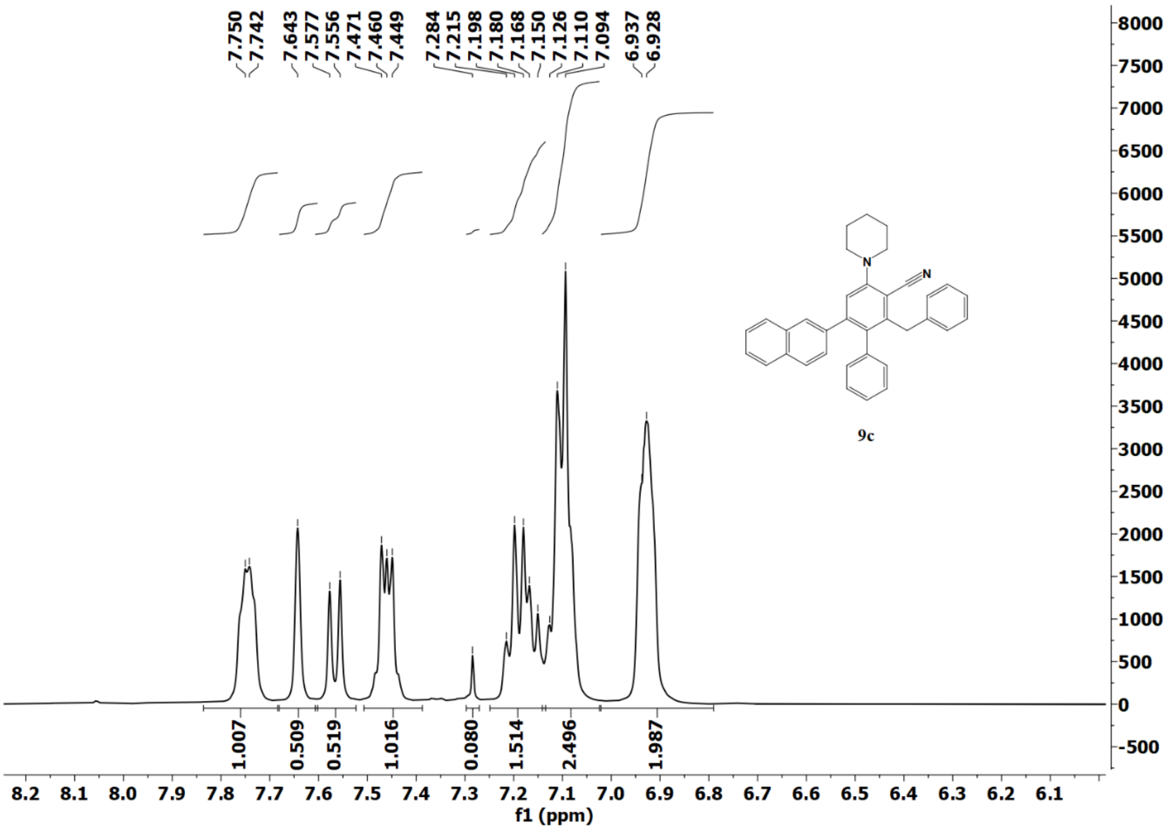

^{13}C NMR (400 MHz, $CDCl_3$): 2-benzyl-6-(naphthalen-2-yl)-4-(piperidin-1-yl)-[1,1'-biphenyl]-3-carbonitrile **9c**:

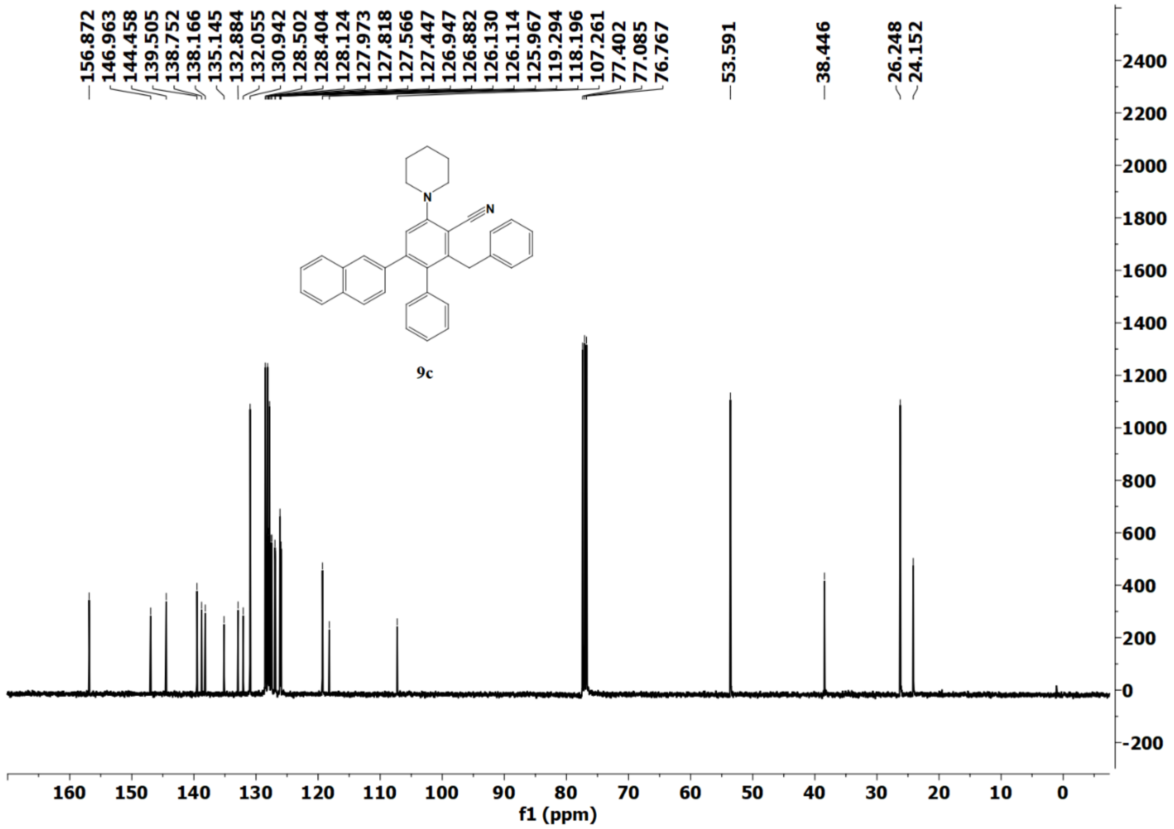

^{1}H NMR (400 MHz, $CDCl_3$): 3’-benzyl-6’-methyl-5’-(-4-phenylpiperazin-1-yl)-[1,1’,2’,1”-terphenyl]-4’-carbonitrile **9d**:

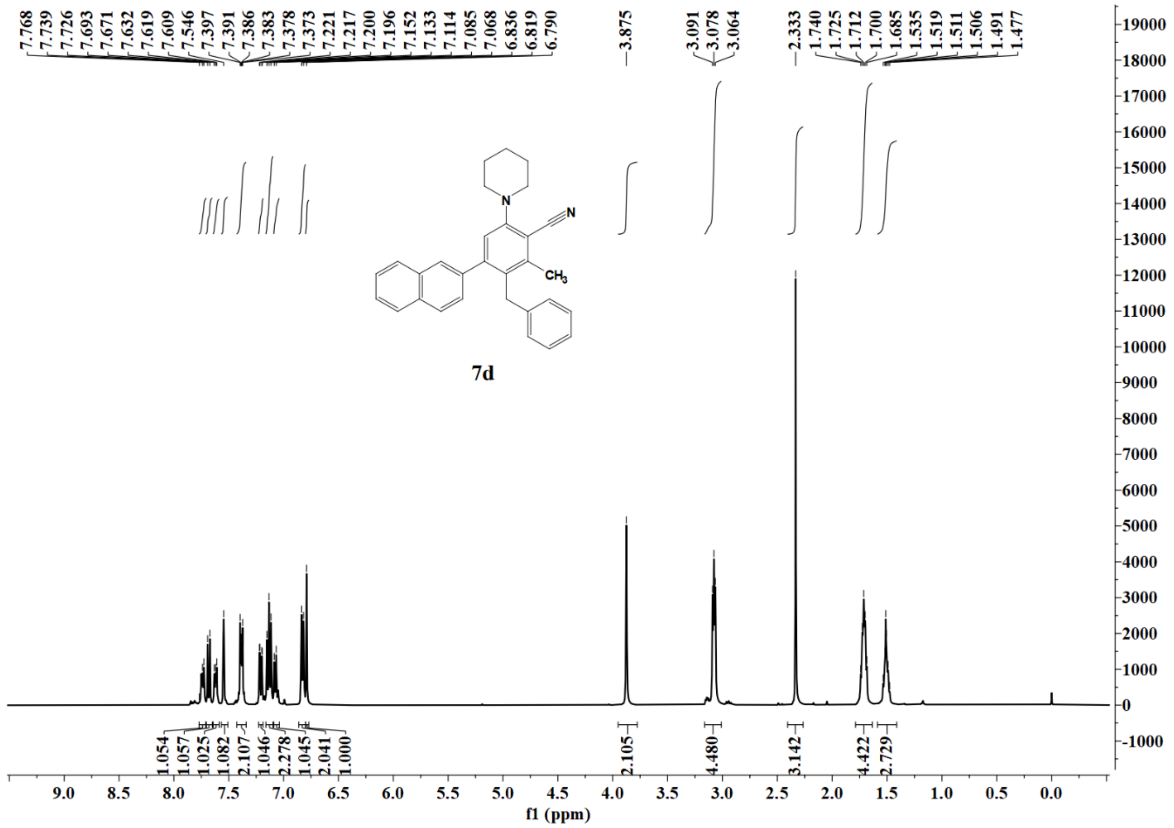

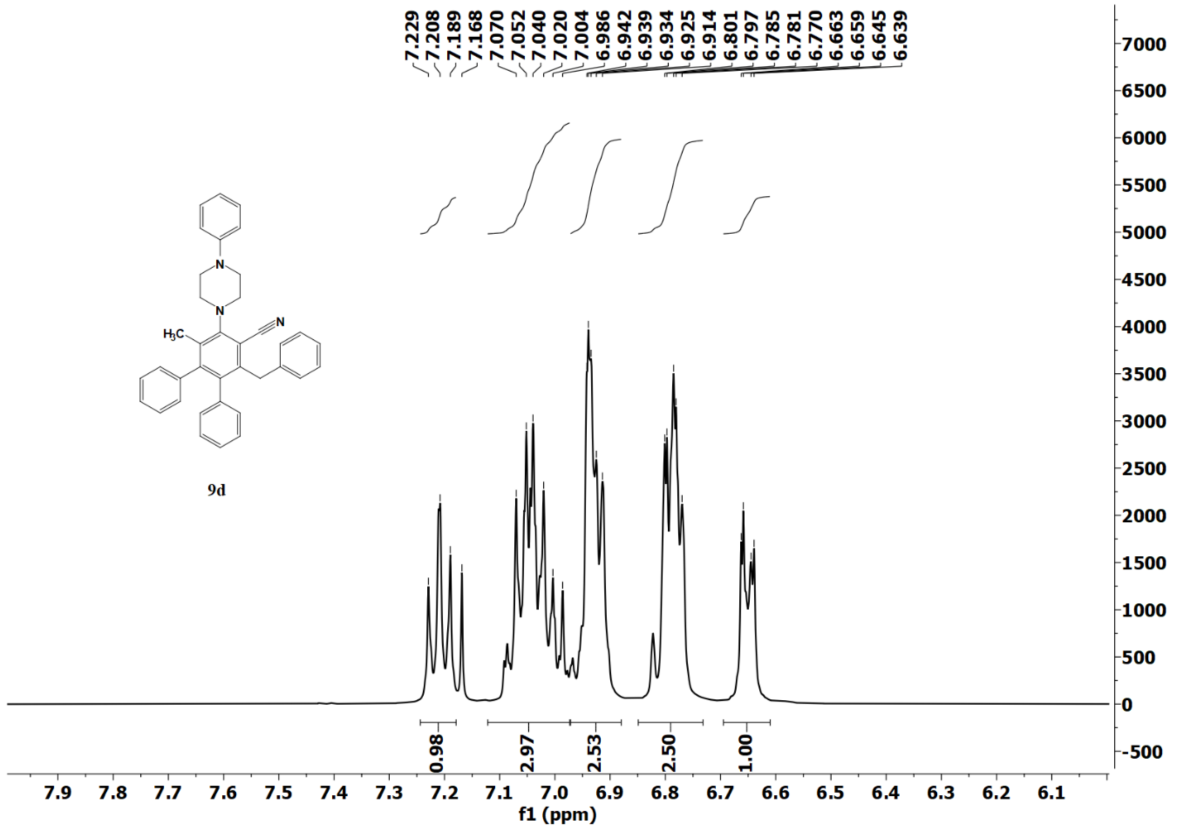

^{13}C NMR (400 MHz, $CDCl_3$): 3’-benzyl-6’-methyl-5’-(-4-phenylpiperazin-1-yl)-[1,1’,2’,1”-terphenyl]-4’-carbonitrile **9d**:

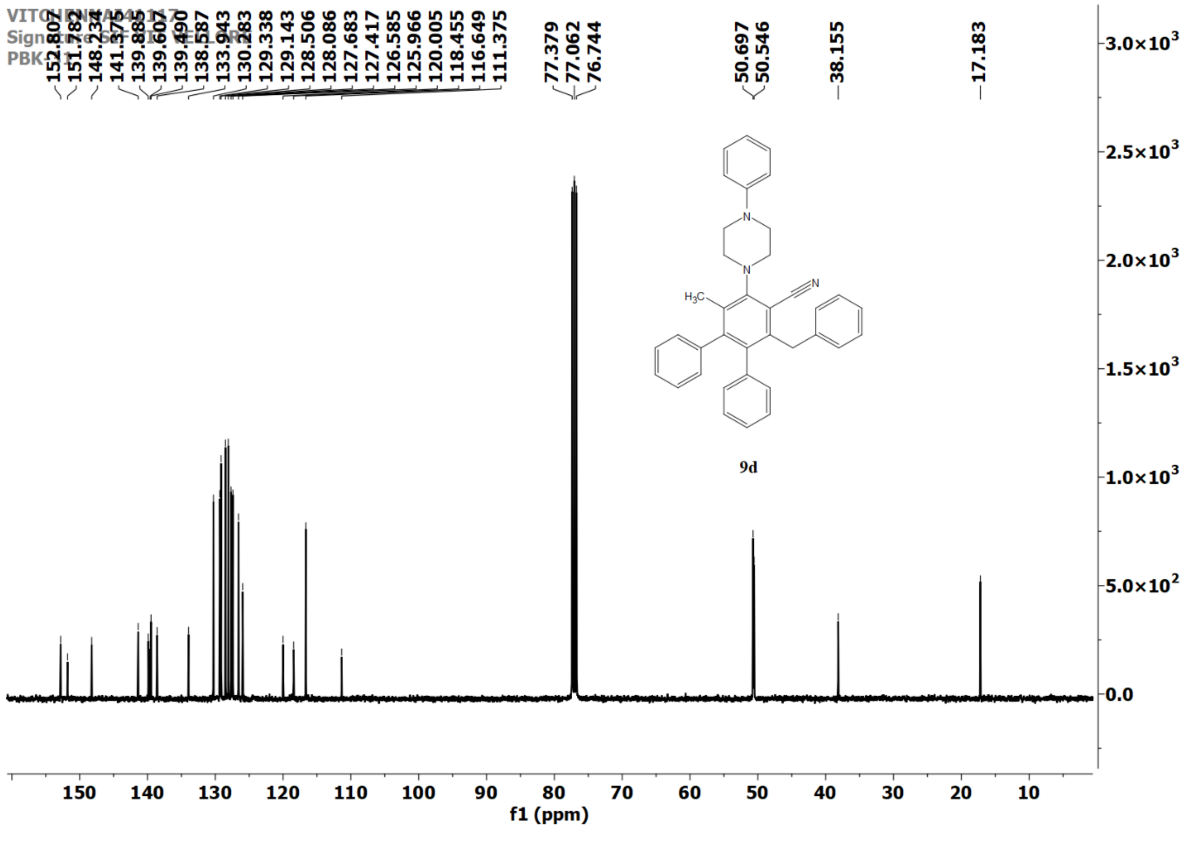

^{1}H NMR (400 MHz, $CDCl_3$): 2-benzyl-6-(naphthalen-1-yl)-4-(piperidin-1-yl)-[1,1’-biphenyl]-3-carbonitrile **9e**:

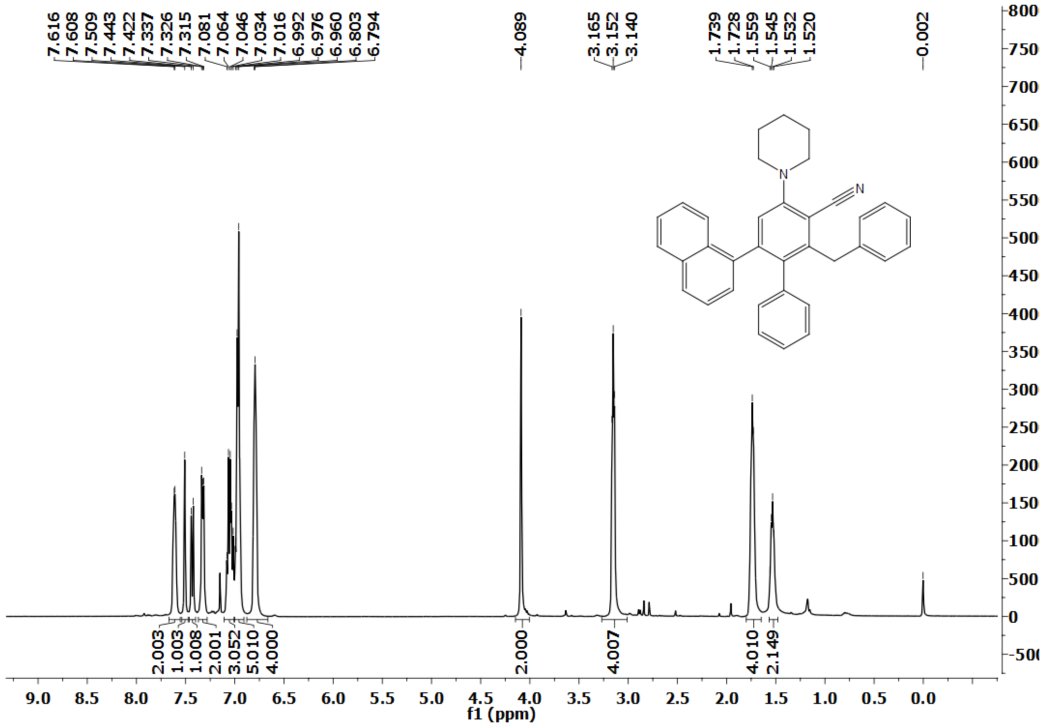

^{13}C NMR (400 MHz, $CDCl_3$): 2-benzyl-6-(naphthalen-1-yl)-4-(piperidin-1-yl)-[1,1’-biphenyl]-3-carbonitrile **9e**:

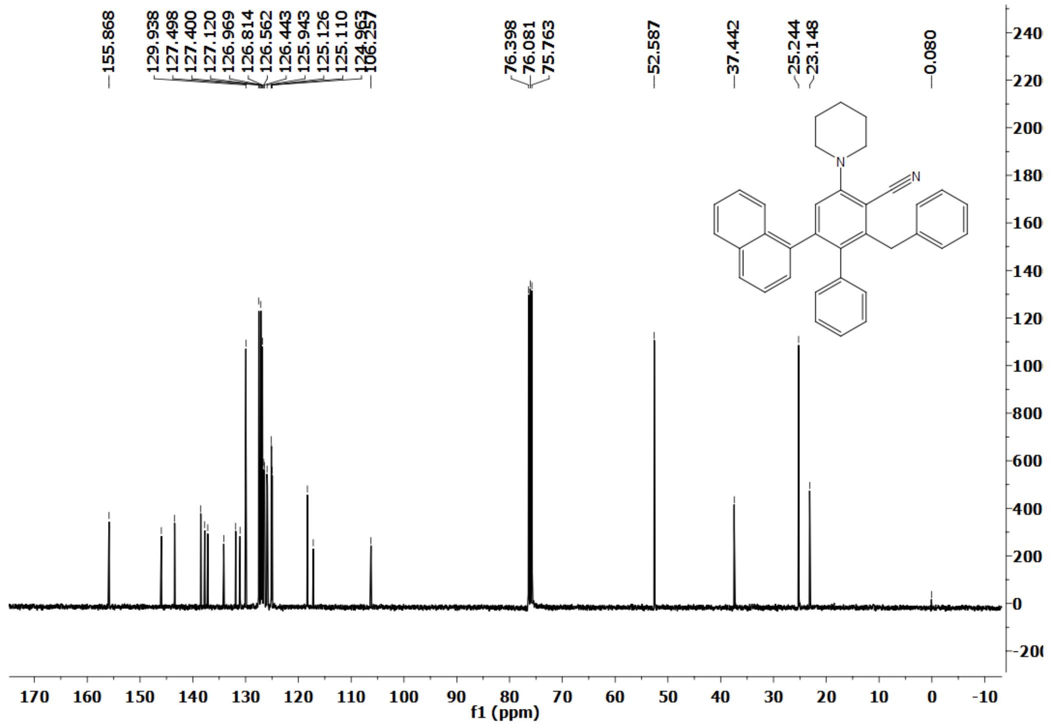

HIGH RESOLUTION MASS SPECTRA:

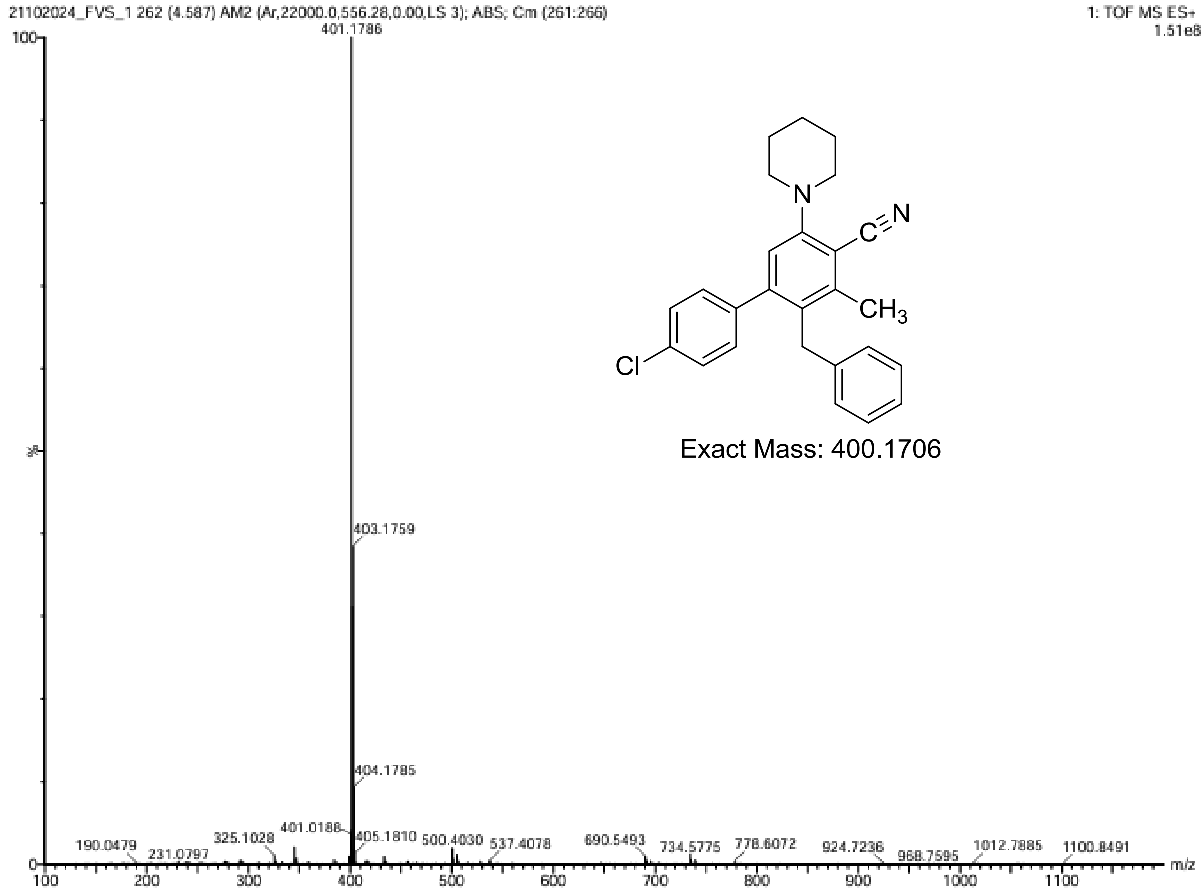

Figure S1 *2-Benzyl-4'-chloro-3-methyl-5-(piperidin-1-yl)[1,1'-biphenyl]-4 carbonitrile* (**7a)**

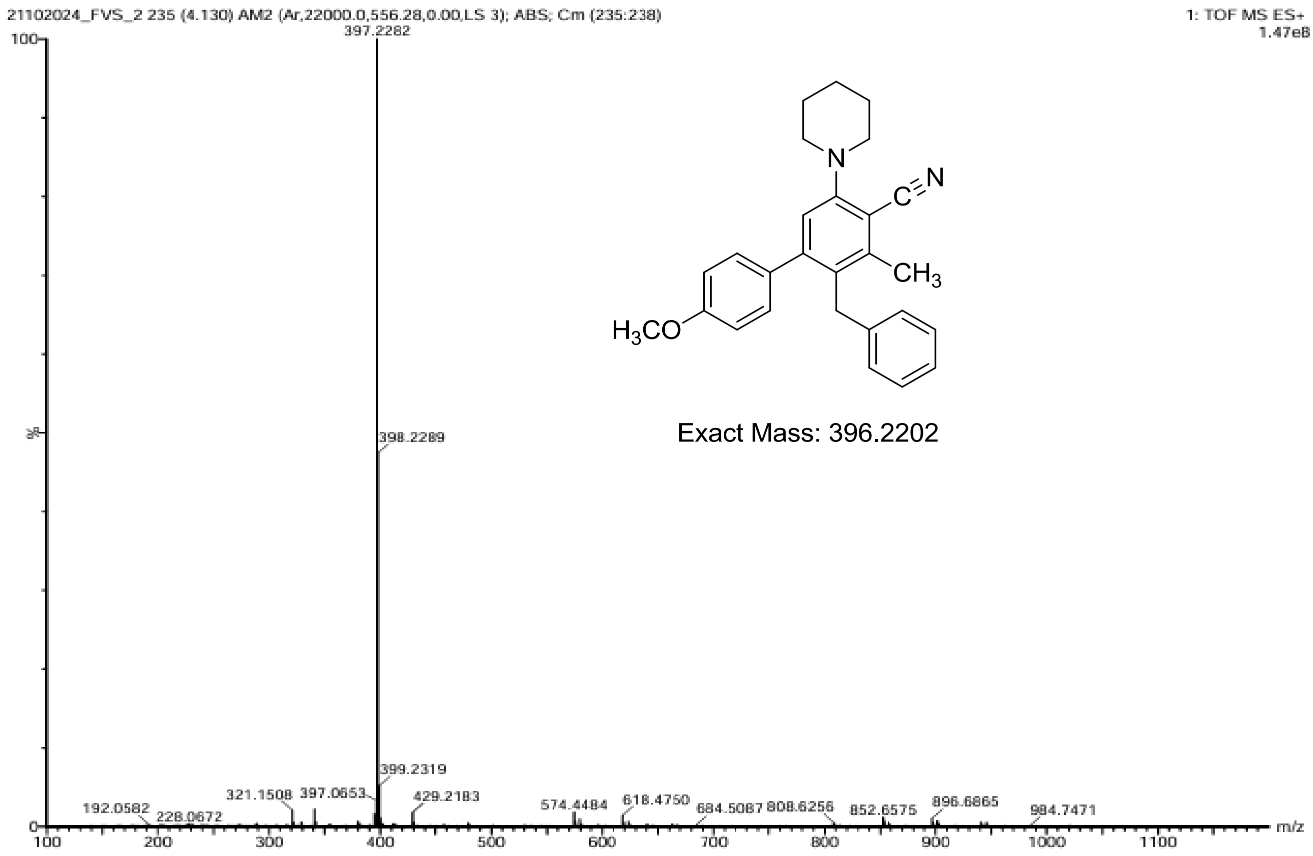

Figure S2: 2-Benzyl-4′-methoxy-3-methyl-5-(piperidin-1-yl)[1,1′-biphenyl] 4-carbonitrile **(7b)**

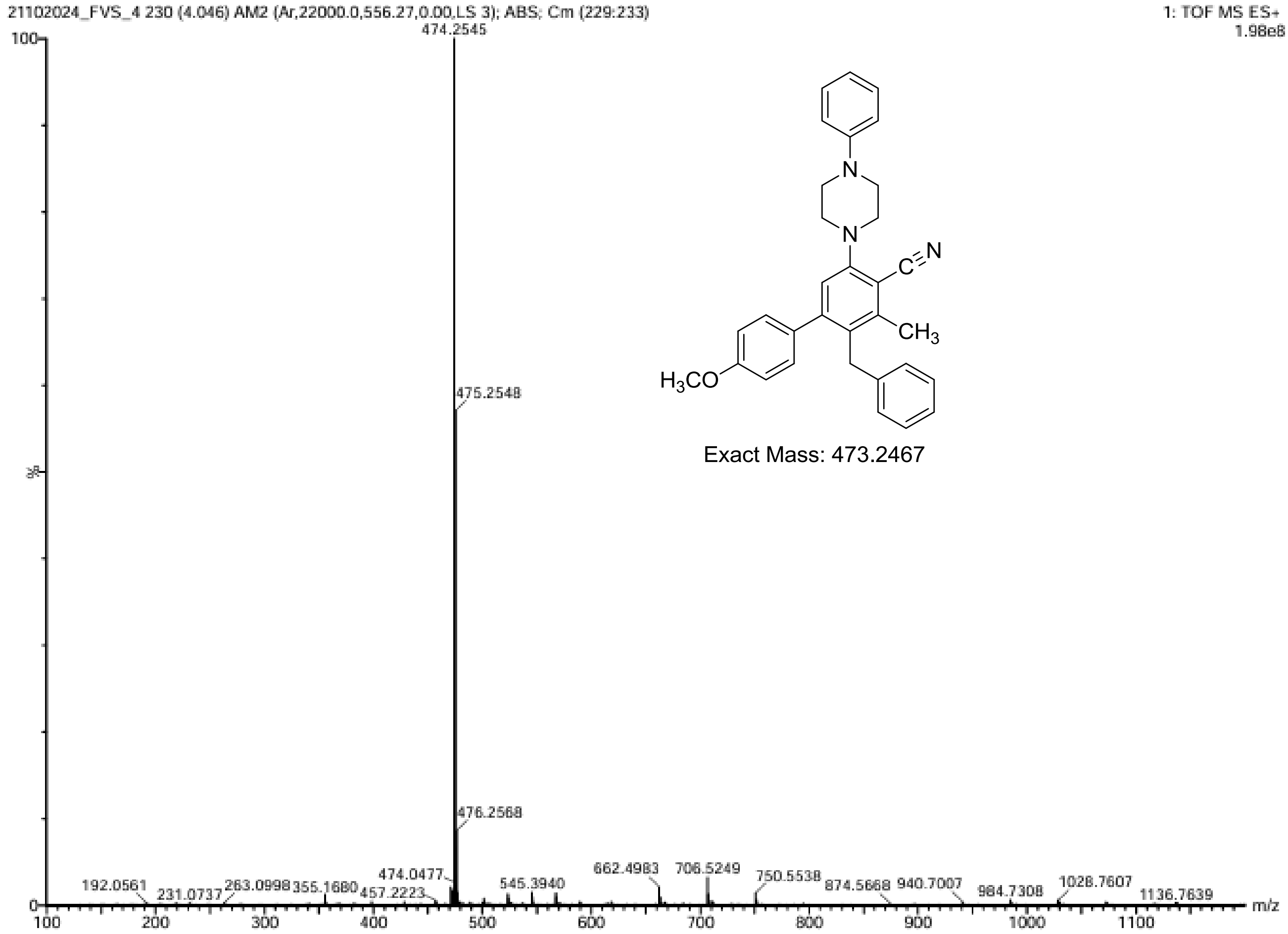

Figure S3: 2-Benzyl-4′-methoxy-3-methyl-5-(4-phenylpiperazin-1-yl)[1,1′biphenyl]-4-carbonitrile (**7c)**

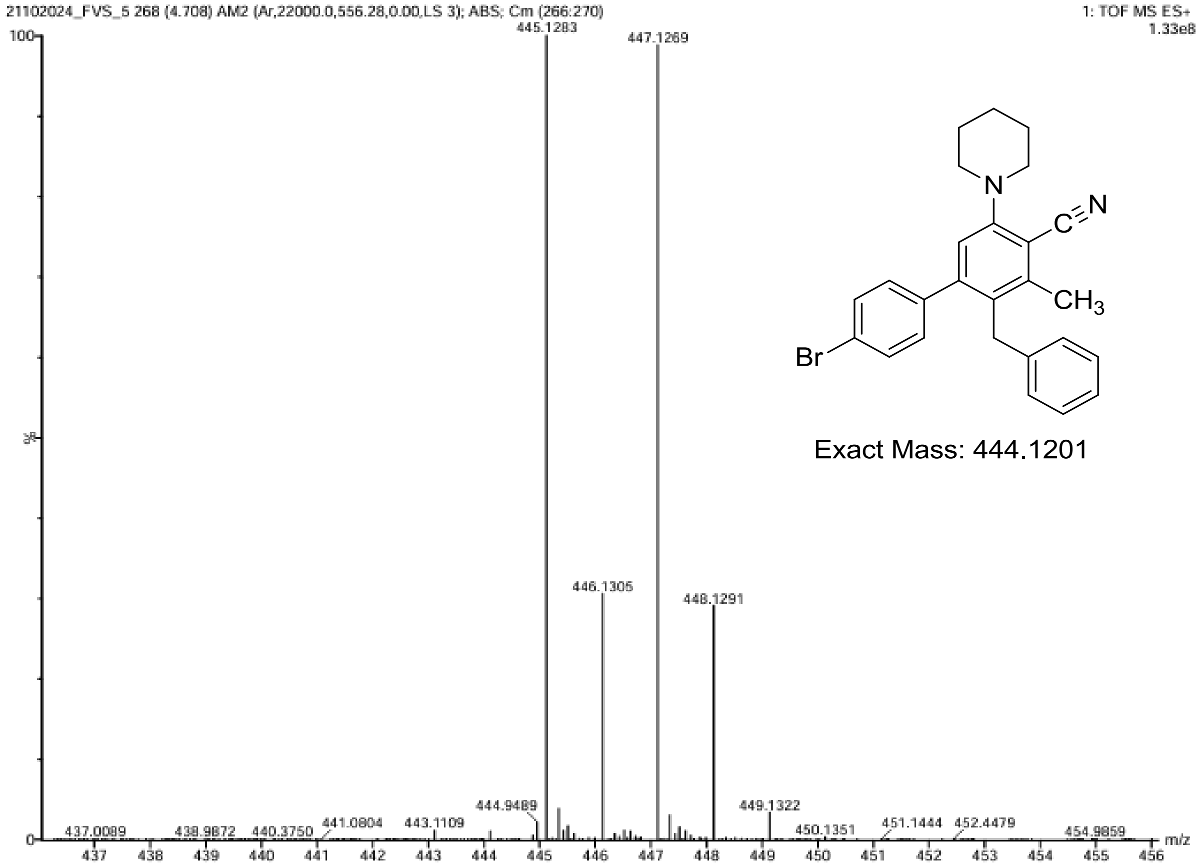

Figure S4: 2-benzyl-4’-bromo-3-methyl-5-(piperidin-1-yl)-[1,1’-biphenyl]-4-carbonitrile **(7e)**

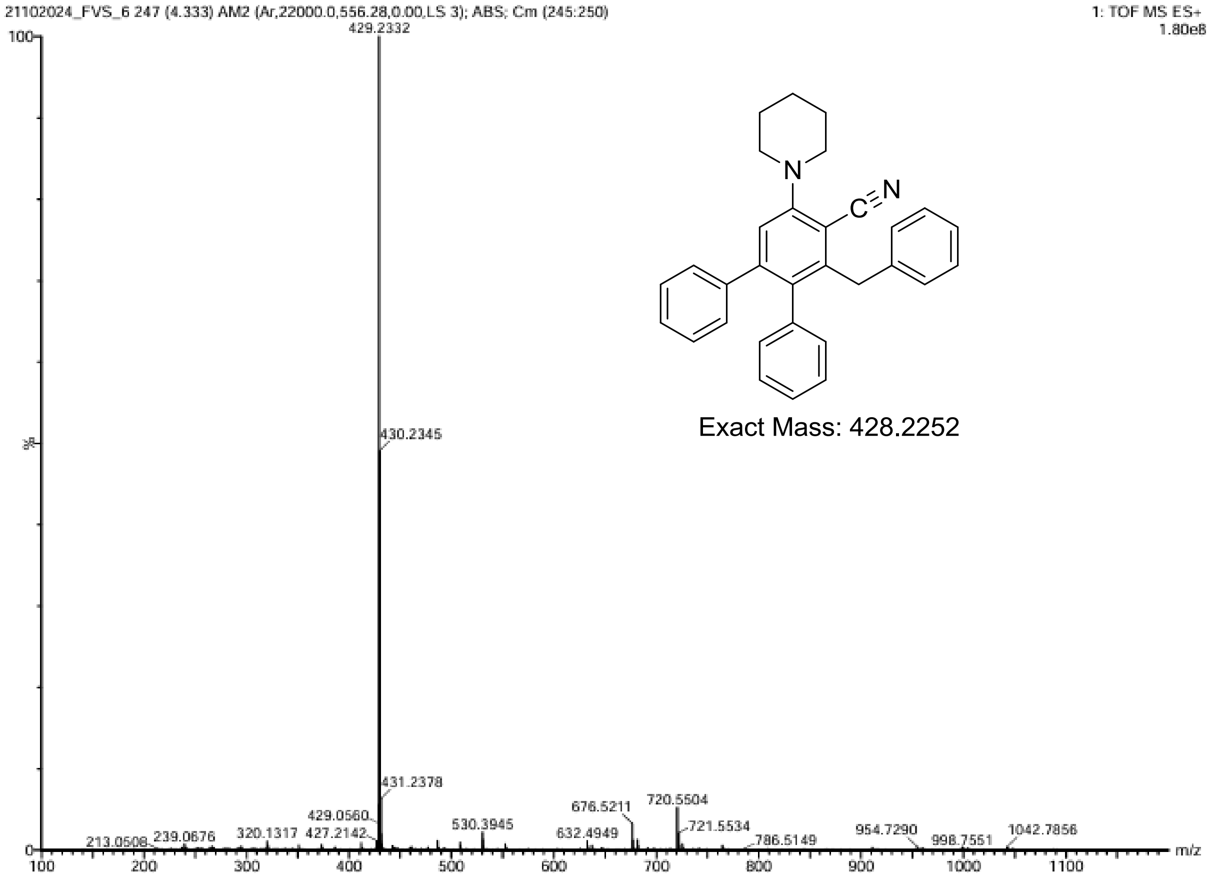

Figure S5: 3’-benzyl-5’-(piperidin-1-yl)-[1,1’,2’,1”-terphenyl]-4’-carbonitrile (**9a)**

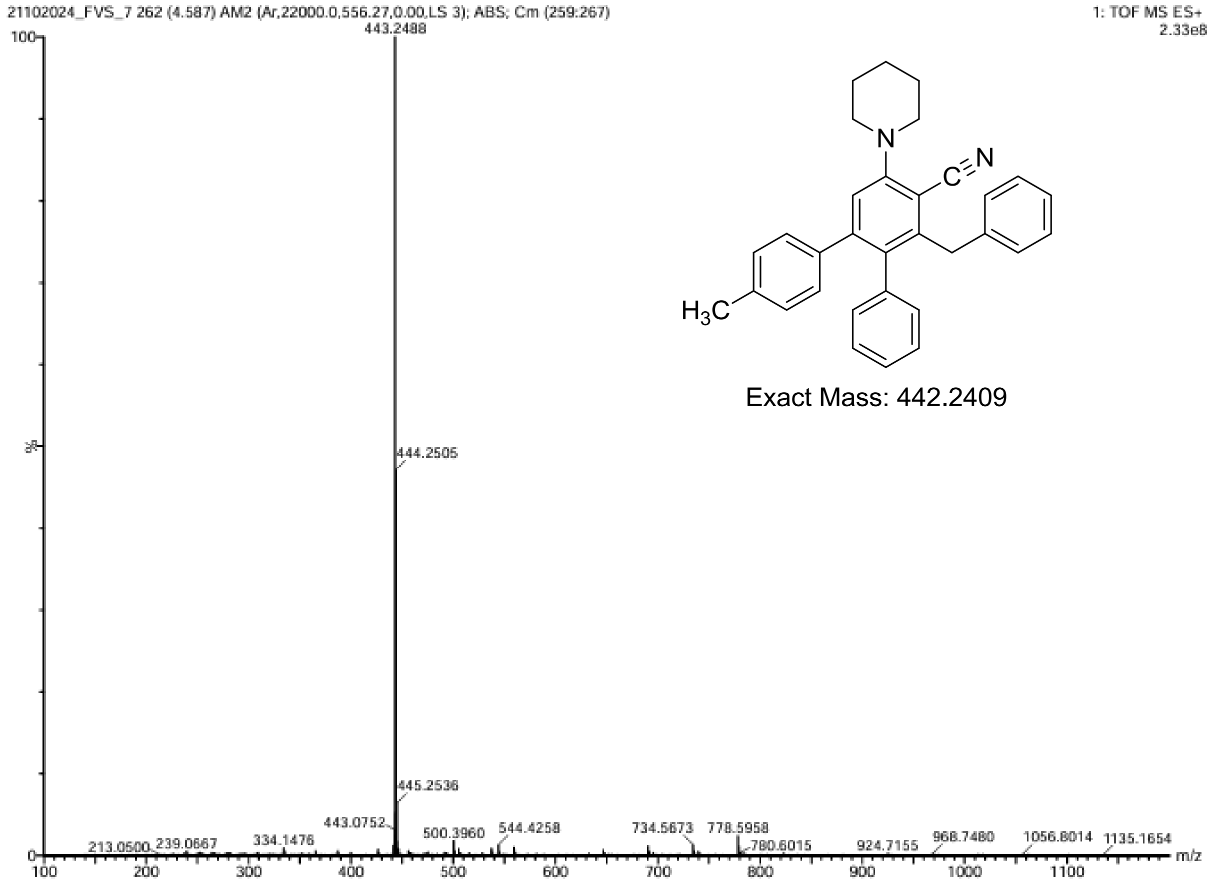

Figure S6: 3'-benzyl-4-methyl-5'-(piperidin-1-yl)-[1,1',2',1"-terphenyl]-4'-carbonitrile (**9b)**

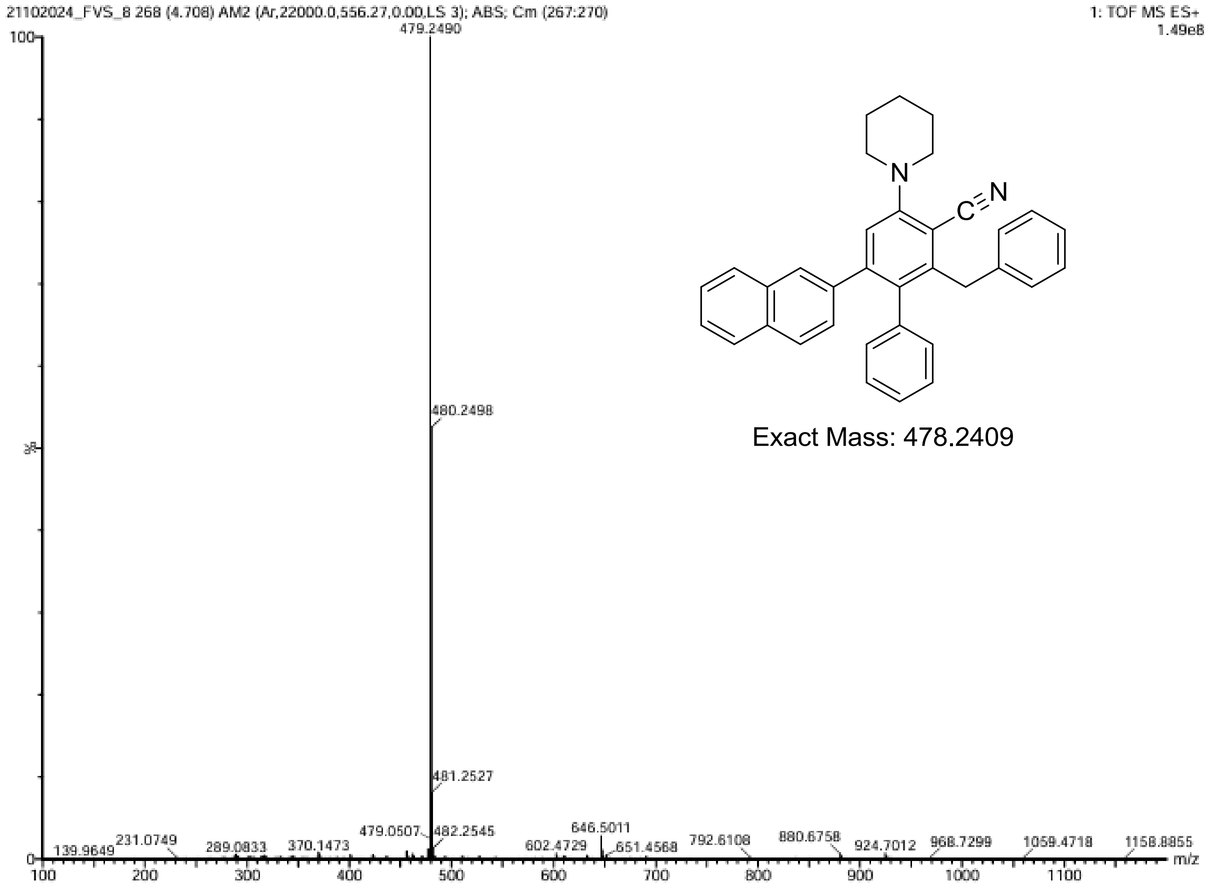

Figure S7: 2-benzyl-6-(naphthalen-2-yl)-4-(piperidin-1-yl)-[1,1'-biphenyl]-3-carbonitrile **(9c)**

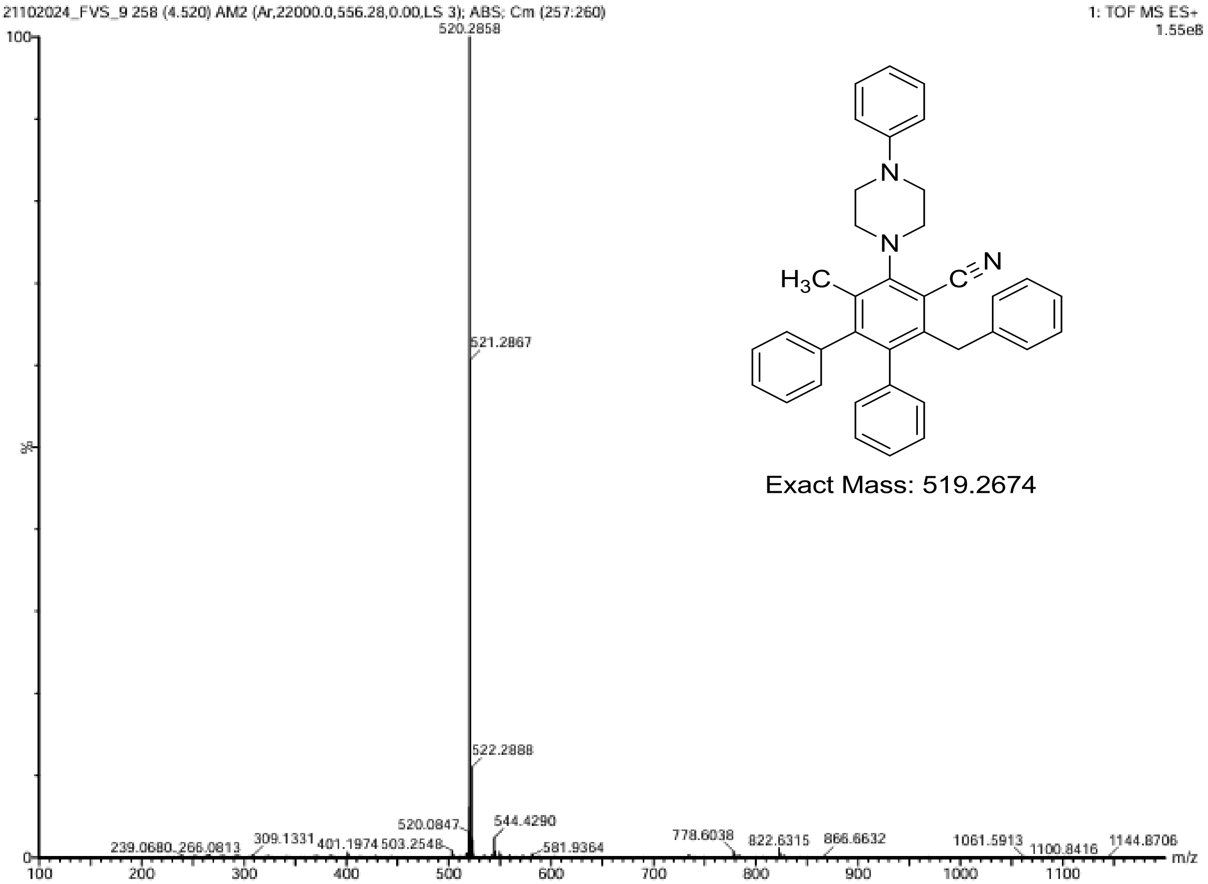

Figure S8: 3’-benzyl-6’-methyl-5’-(-4-phenylpiperazin-1-yl)-[1,1’,2’,1”-terphenyl]-4’-carbonitrile (**9d)**

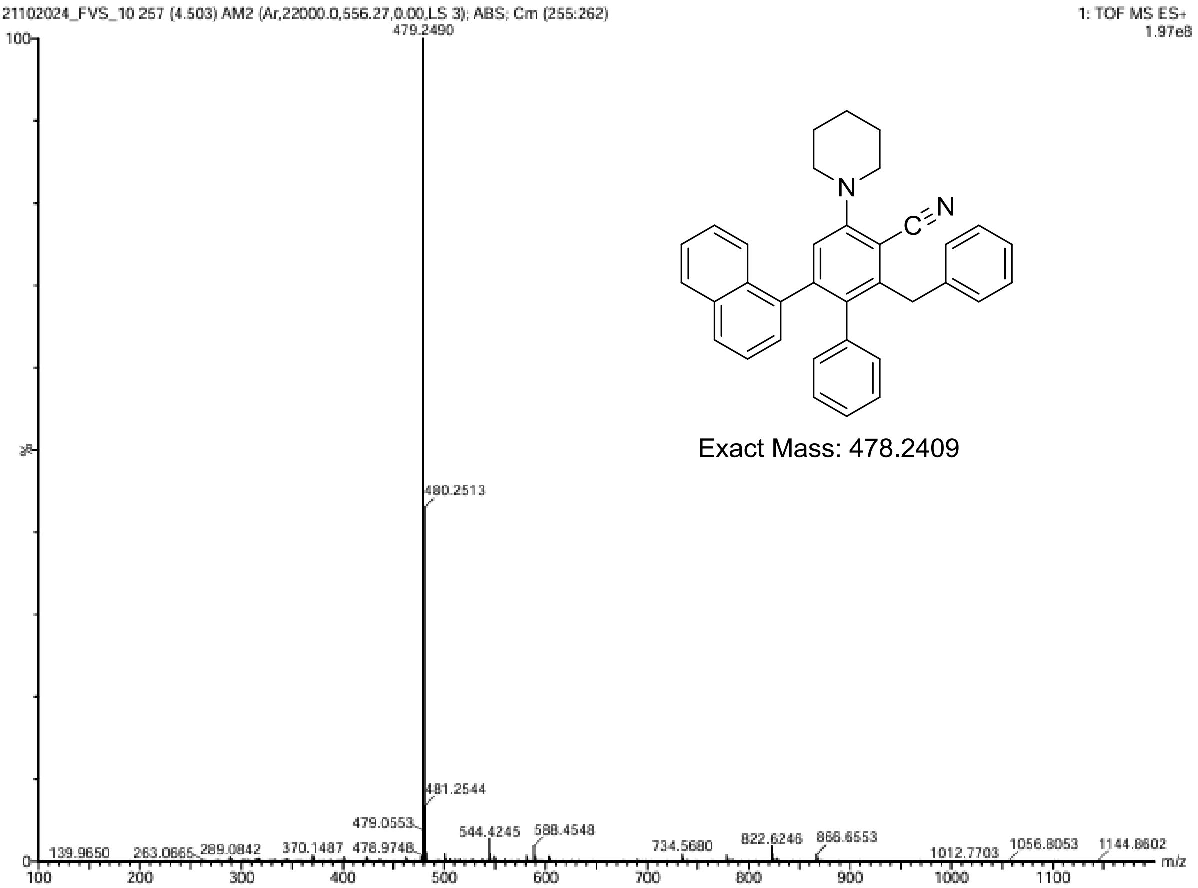

Figure S9: 2-benzyl-6-(naphthalen-1-yl)-4-(piperidin-1-yl)-[1,1'-biphenyl]-3-carbonitrile **(9e)**

Table S1. Computationally evaluated electronic structure properties in the ground and excited state of various diarylmethanes

Entry	E_0 (eV)	E_1 (eV)	μ_g (D)	Charge on heteroatoms S_0	Transition energies		
					$S_0 \rightarrow S_1$	$S_0 \rightarrow S_2$	$S_0 \rightarrow S_3$
**7a**	-42874.0196	-42873.3678	2.18	N_{13} 0.1445<br>Cl_{28} 0.2395<br>N_{29} -0.5009	348.65 nm (f=0.078)<br>H → L (95.8 %)	290.93 nm (f=0.0337)<br>H → L+1 (97.0 %)	279.23 nm (f=0.0094)<br>H-1 → L (5.76 %)<br>H → L+2 (89.41 %)
**7b**	-33490.7193	-33487.1220	5.05	N_{13} 0.4573<br>Cl_{28} -0.1523<br>N_{29} -0.1645	342.90 (f=0.0933)<br>H→L (94.6 %)	290.68 (f=0.1889)<br>H-1→L (83.3 %)<br>H→L+1 (12.0 %)	283.18 (f=0.1536)<br>H-1→L (12.0 %)<br>H→L+1 (83.1 %)
**7c**	-40215.8488	-40212.1907	5.83	N_{13} 0.4490<br>N_{16} 0.4177<br>O_{28} -0.1522<br>N_{29} -0.1686	336.89 nm (f=0.0036)<br>H-1→L (2.09 %)<br>H → L (97.2 %)	322.65 nm (f=0.0801)<br>H-1→L (90.8 %)<br>H → L (2.3 %)	289.92 nm (f=.1664)<br>H-2→L (95.0 %)
**7d**	-34555.4577	-34551.8885	4.56	N_{13} 0.4943<br>N_{28} -0.1731	344.43 nm (f=0.0636)<br>H→L (91.4 %)<br>H→L+1 (5.1 %)	311.67 (f=0.0407)<br>H→L (5.6 %)<br>H→L+1 (88.5 %)	303.19 nm (f=0.0553)<br>H-1→L (87.6 %)<br>H-1→L+1 (4.9 %)
**7e**	-100403.3248	-100399.6460	2.39	N_{13} 0.485860<br>N_{28} -0.176807<br>Br_{53} -0.164401	337.02 nm (f=0.0802<br>H→L (95.37 %)	289.62 nm (f=0.0437<br>H→L+1 (95.10 %)	275.87 nm (f=0.0074<br>H→L+2 (92.17 %)
**9a**	-35592.1559	-35588.6441	4.75	N_{26} 0.3257<br>N_{33} -0.1774	353.05 nm (f=0.1037)<br>H→L (95.63 %)	294.06 nm (f=0.1202)<br>H-1→L (3.75 %)<br>H→L+1 (90.40 %)	279.87 nm (f=0.0484)<br>H-4→L (2.37 %)<br>H-2→L (2.62 %)<br>H-1→L (89.72 %)<br>H→L+1 (2.60 %)
**9b**	-36662.4163	-36658.7492	4.93	N_{26} 0.4064<br>N_{33} -0.1654	338.10 nm (f=0.0874)<br>H→L (94.27 %)	287.16 nm (f=0.1859)<br>H-1→L (82.96 %)<br>H→L (2.23 %)<br>H→L+1 (10.44 %)	278.53 nm (f=0.1900)<br>H-5→L (2.15 %)<br>H-4→L (2.42 %)<br>H-3→L (2.12%)<br>H-2→L (9.02 %)

							H-1→L (6.05 %)<br>H→L+1 (72.42 %)
**9c**	-39774.0406	-39770.4579	4.40	N_{26} 0.4106<br>N_{33} -0.2153	346.06 nm (f=0.0857)<br>H→L (90.15 %)<br>H→L+1 (4.69 %)	313.43 nm (f=0.0686)<br>H-1→L (82.14 %)<br>H-1→L+1 (5.34 %)<br>H→L (2.79 %)<br>H→L+1 (3.44 %)	311.33 nm (f=0.0147)<br>H-3→L (2.16 %)<br>H-2→L (3.37 %)<br>H-1→L (2.89 %)3<br>H→L+1 (82.85 %)
**9d**	-43386.9922	-43383.5554	5.44	N_{26} 0.3470<br>N_{30} 0.2688<br>N_{33} -0.1550	360.75 nm (f=0.0189)<br>H-1→L (10.02 %)<br>H→L (88.68 %)	335.95 nm (f=0.0362)<br>H-1→L (86.31 %)<br>H→L (10.62 %)	303.56 nm (f=0.0236)<br>H-1→L+1 (5.95 %)<br>H→L+1 (88.33 %)<br>H→L+3 (2.54 %)
**9e**	-39773.9810	-39770.3510	4.31	N_{26} 0.3852<br>N_{33} -0.1837	341.55 nm (f=0.0869)<br>H→L (90.10 %)<br>H→L+1 (5.72 %)	316.12 nm (f=0.0582)<br>H-1→L (76.56 %)<br>H-1→L+1 (9.35 %)<br>H→L (3.65 %)<br>H→L+1 (8.18 %)	313.75 nm (f=0.0227)<br>H-1→L+1 (11.52 %)<br>H→L (4.35 %)<br>H→L+1 (82.38 %)

7a 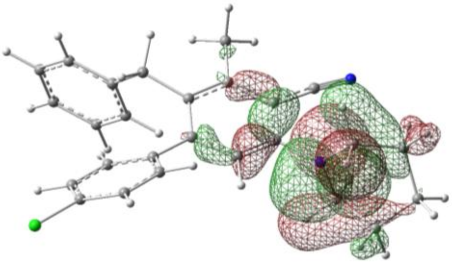 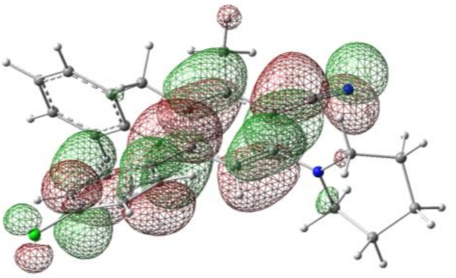 **2.99**

7b 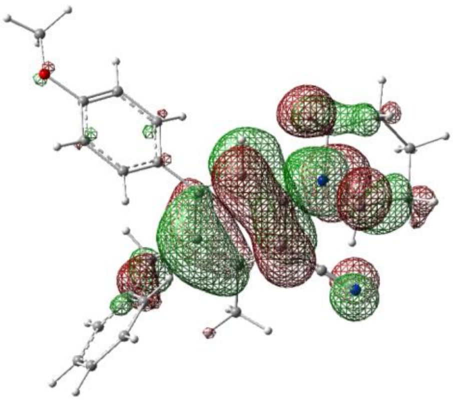 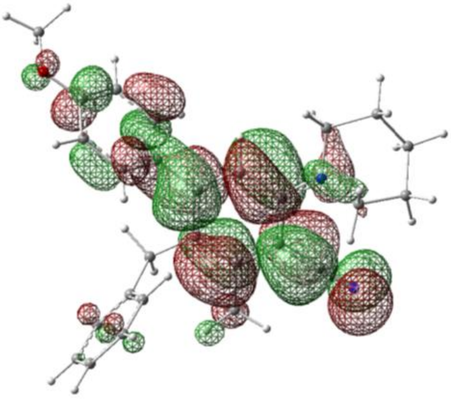 **4.11**

7c 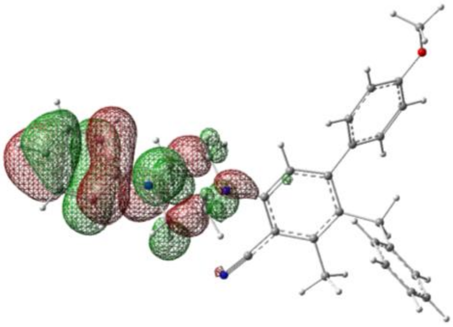 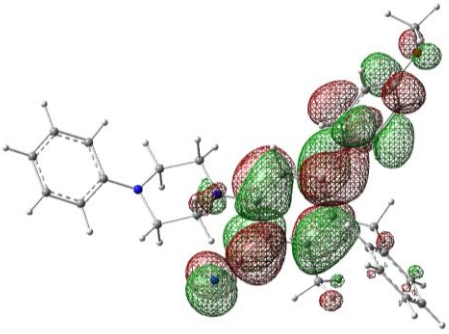 **3.06**

7d 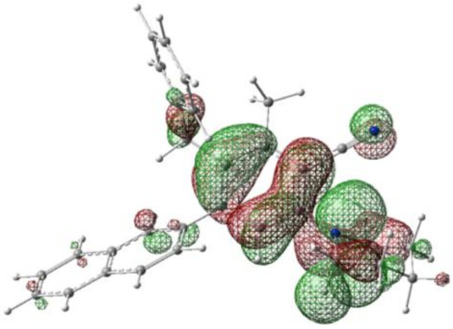 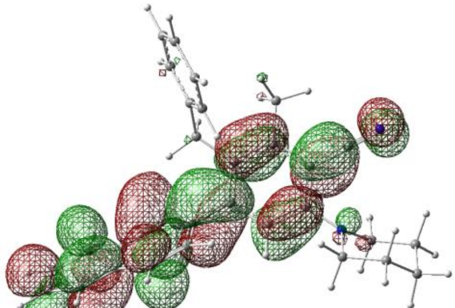 **3.46**

7e 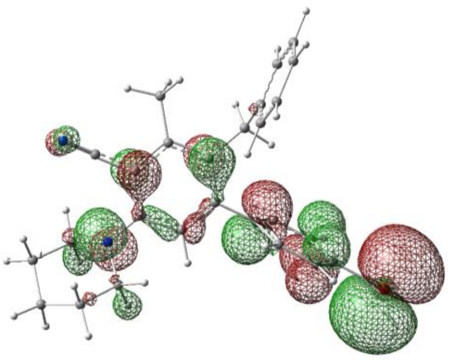 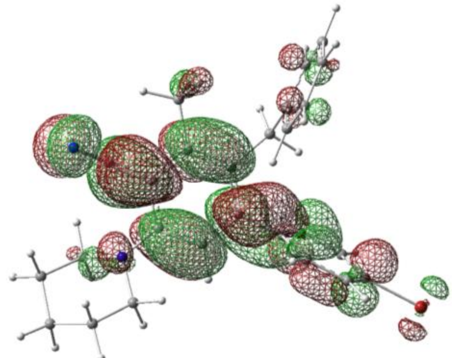 **2.88**

9a 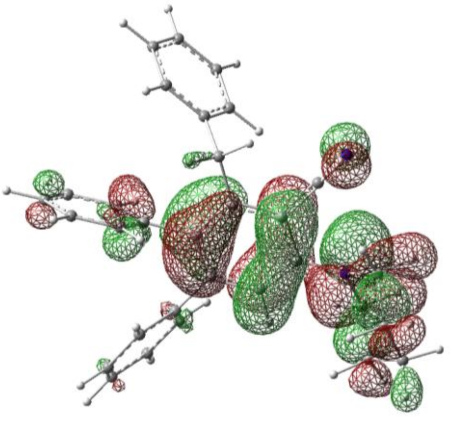 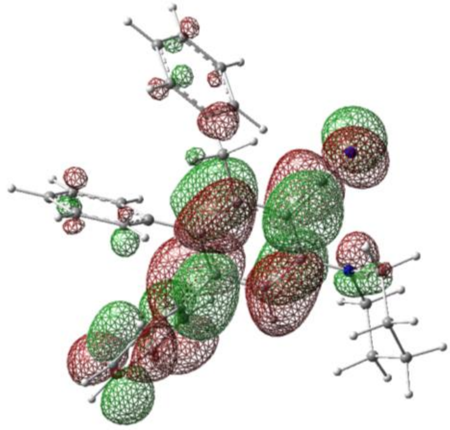 **4.12**

9b 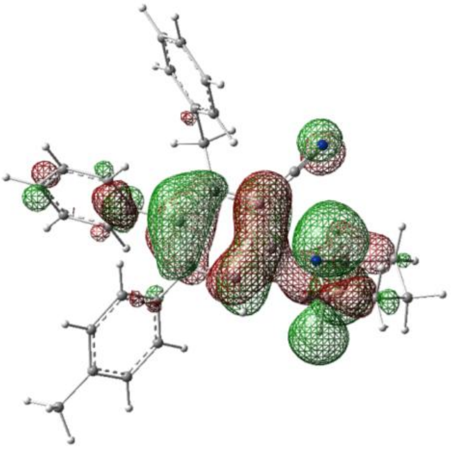 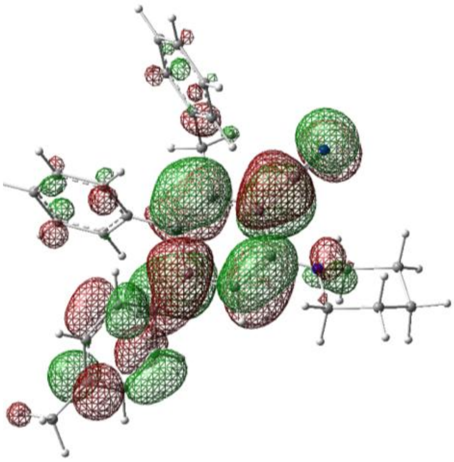 **4.32**

9c 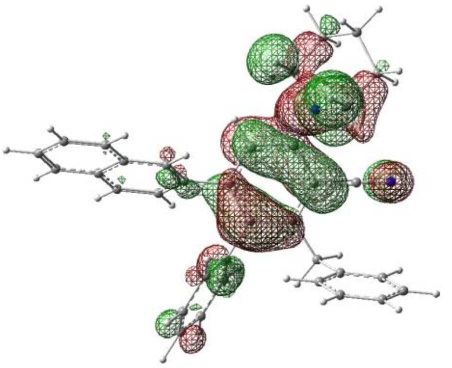 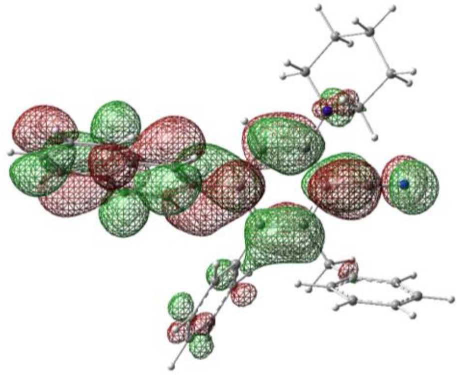 **4.16**

9d 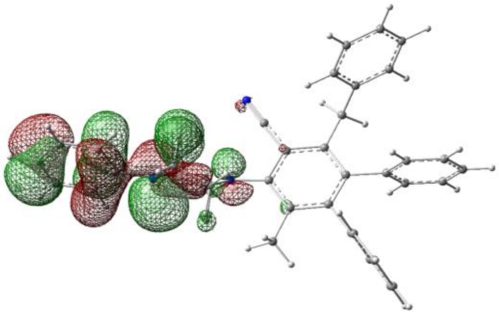 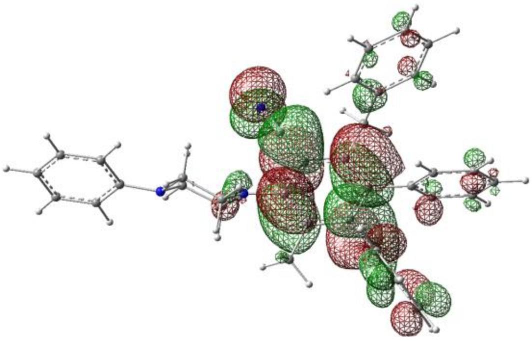 **3.93**

9e 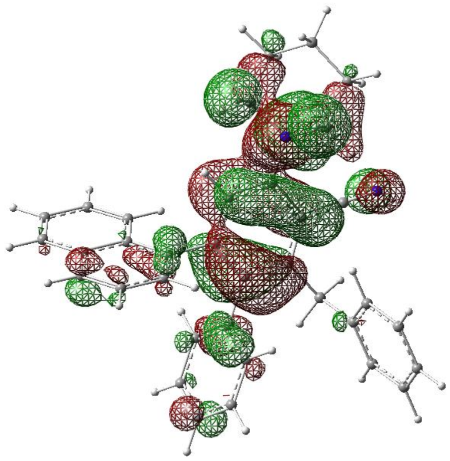 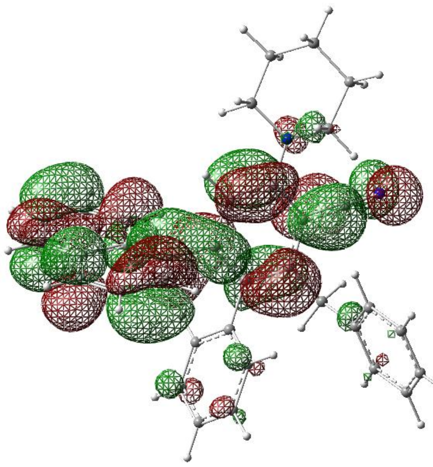 **4.25**

Figure S10. Representation of charge density distribution in the highest occupied (HOMO) and lowest unoccupied (LUMO) molecular orbitals of various DAMs (**7a-e** and **9a-e**) along with their energy gap in eV.

7a

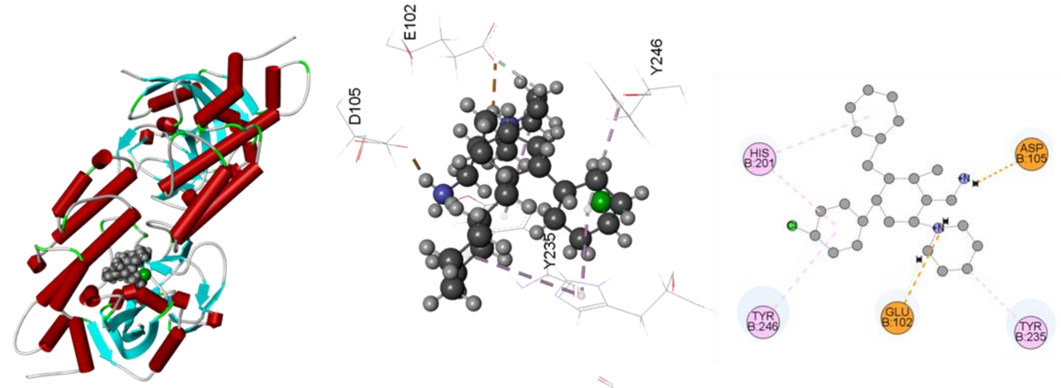
E102
D105
Y246
Y235

7b

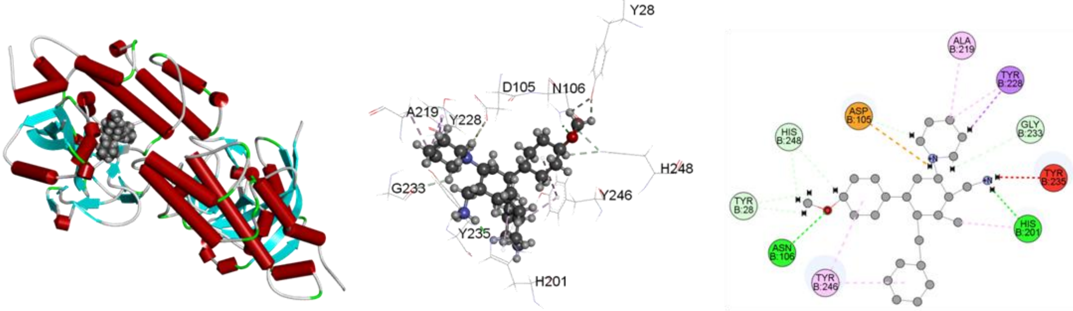
Y28
D105
N106
A219
Y228
H248
G233
Y246
Y235
H201

7c

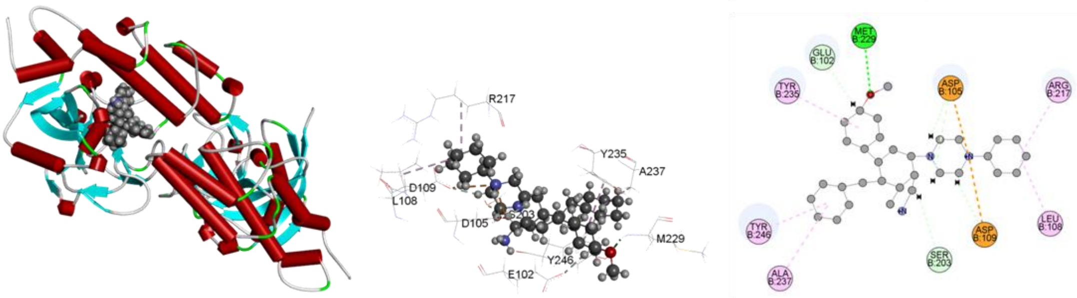
R217
Y235
A237
D109
L108
D105
M229
Y246
E102

7d

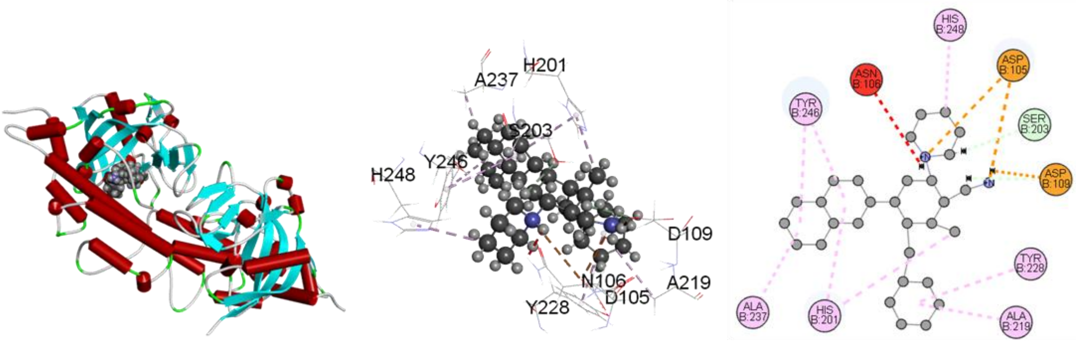
H201
A237
S203
H248
Y246
D109
N106
A219
D105
Y228

7e

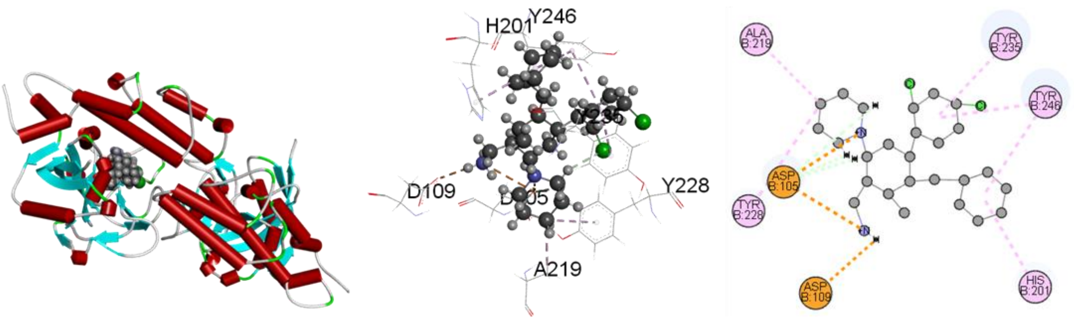
H201
Y246
Y235
D109
Y228
A219

9a

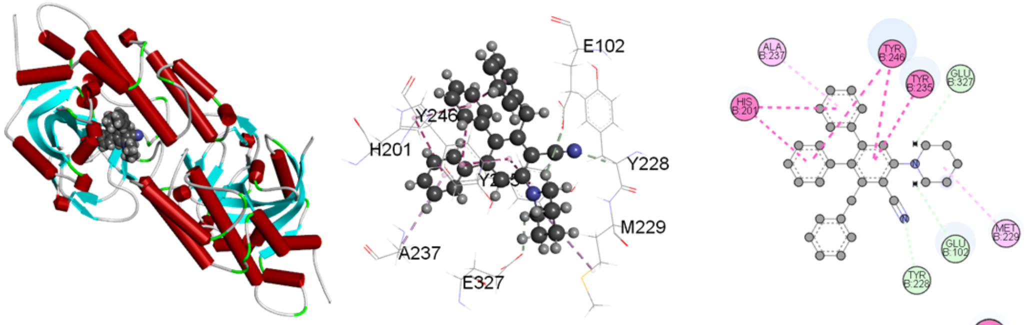
E102
Y246
H201
Y228
M229
A237
E327
ALA B:237
TYR B:246
TYR B:235
GLU B:327
HIS B:201
MET B:229
GLU B:102
TYR B:228

9b

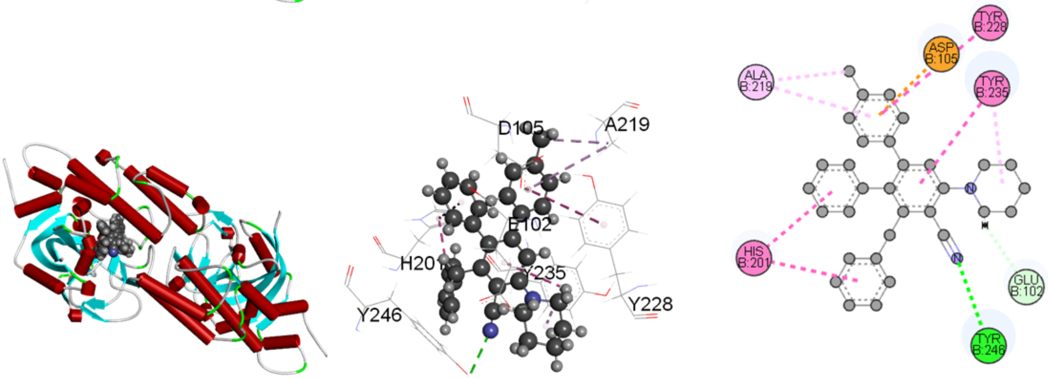
D105
A219
E102
H201
Y235
Y246
Y228
TYR B:228
ASP B:105
ALA B:219
TYR B:235
HIS B:201
GLU B:102
TYR B:246

9c

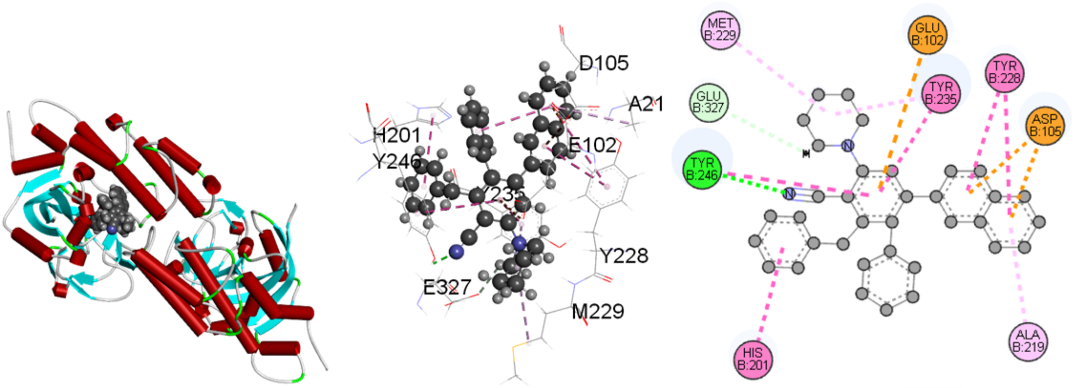
D105
H201
E102
Y228
E327
M229
MET B:229
GLU B:102
GLU B:327
TYR B:235
TYR B:228
ASP B:105
TYR B:246
HIS B:201
ALA B:219

9d

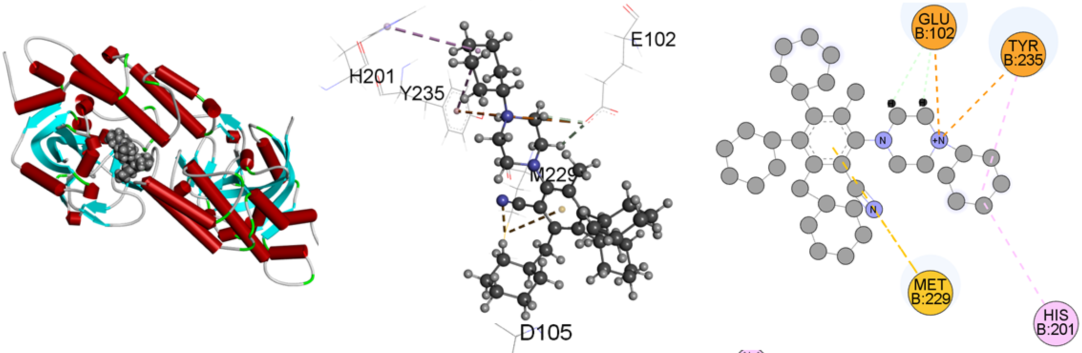
E102
H201
Y235
M229
GLU B:102
TYR B:235
MET B:229
HIS B:201

9e

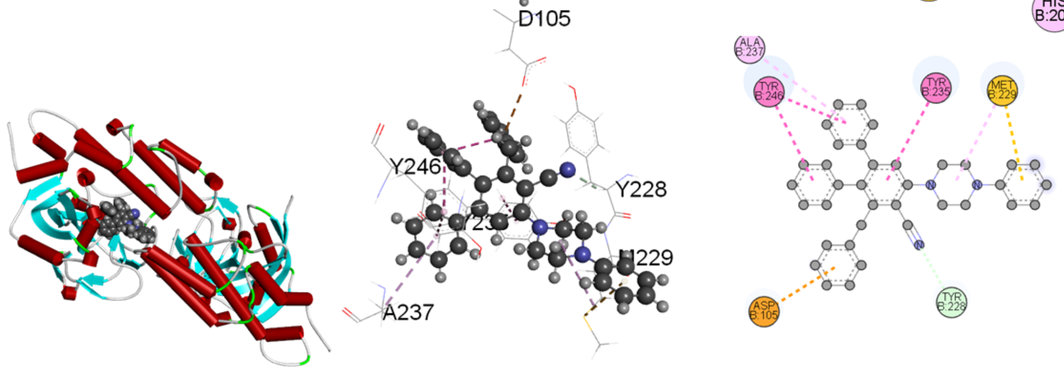
D105
Y246
Y228
A237
ALA B:237
TYR B:246
TYR B:235
MET B:229
ASP B:105
TYR B:228

Figure S11. Schematic representing the docked interactions of various diarylmethane derivatives **7a-e** and **9a-e** with PARP1 (a) 3D representation of protein – ligand complex in the binding pocket (b) 2-dimensional representation of the binding of interacting residues and various derivatives (c) 2-dimensional representation showing the type of interactions involved in the binding of PARP1 with various derivatives.

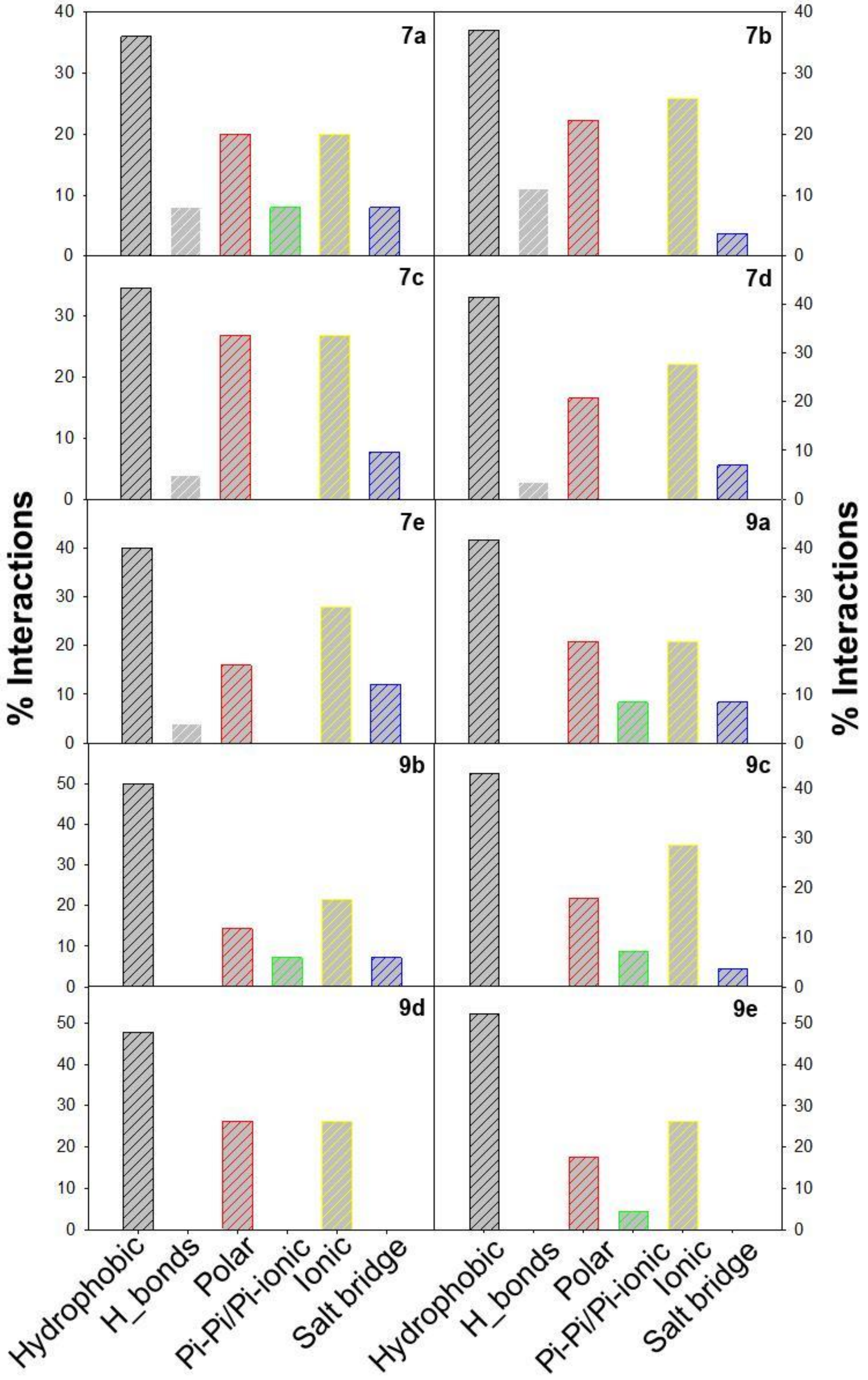

Figure S12. % Contribution of various stabilizing interactions between **7a-e** and **9a-e** and PARP1 binding pocket residues
